# Universal Amplification-Free RNA Detection by Integrating CRISPR-Cas10 with Aptameric Graphene Field-Effect Transistor

**DOI:** 10.1007/s40820-025-01730-3

**Published:** 2025-04-30

**Authors:** Mingyuan Sun, Zhenxiao Yu, Shuai Wang, Jiaoyan Qiu, Yuzhen Huang, Xiaoshuang Chen, Yunhong Zhang, Chao Wang, Xue Zhang, Yanbo Liang, Hong Liu, Qunxin She, Yu Zhang, Lin Han

**Affiliations:** 1 Institute of Marine Science and Technology, Shandong University, Qingdao, Shandong 266237 People’s Republic of China; 2 CRISPR and Archaea Biology Research Center, State Key Laboratory of Microbial Technology and Microbial Technology Institute, Shandong University, Qingdao, Shandong 266237 People’s Republic of China; 3 State Key Laboratory of Crystal Materials, Shandong University, Jinan, Shandong 250100 People’s Republic of China; 4 School of Integrated Circuits, Shandong University, Ji’nan, Shandong 250100 People’s Republic of China; 5 Shandong Engineering Research Center of Biomarker and Artificial Intelligence Application, Jinan, 250100 People’s Republic of China

**Keywords:** CRISPR, Cas10; Graphene effect, Field transistor; Biosensor; RNA/miRNA detection; Amplification, Free

## Abstract

Amplification-free, highly sensitive, and specific nucleic acid detection is crucial for health monitoring and diagnosis. The type III CRISPR-Cas10 system, which provides viral immunity through CRISPR-associated protein effectors, enables a new amplification-free nucleic acid diagnostic tool. In this study, we develop a CRISPR-graphene field-effect transistors (GFETs) biosensor by combining the type III CRISPR-Cas10 system with GFETs for direct nucleic acid detection. This biosensor exploits the target RNA-activated continuous ssDNA cleavage activity of the dCsm3 CRISPR-Cas10 effector and the high charge density of a hairpin DNA reporter on the GFET channel to achieve label-free, amplification-free, highly sensitive, and specific RNA detection. The CRISPR-GFET biosensor exhibits excellent performance in detecting medium-length RNAs and miRNAs, with detection limits at the aM level and a broad linear range of 10^−15^ to 10^−11^ M for RNAs and 10^−15^ to 10^−9^ M for miRNAs. It shows high sensitivity in throat swabs and serum samples, distinguishing between healthy individuals (N = 5) and breast cancer patients (N = 6) without the need for extraction, purification, or amplification. This platform mitigates risks associated with nucleic acid amplification and cross-contamination, making it a versatile and scalable diagnostic tool for molecular diagnostics in human health.

**Supplementary Information:**

The online version contains supplementary material available at 10.1007/s40820-025-01730-3.

## Highlights

- A collaborative biosensing system is developed for amplification-free RNA/miRNA detection by integrating Type III CRISPR-Cas10 system with a graphene field-effect transistor (GFET).
- Continuous cleavage of ssDNA by the mutant CRISPR-Cas10 effector complexes and high charge density of hairpin DNA reporters on the GFET channel enable the detection limit to reach the level of aM.
- A universal sensing detection platform is established to directly detect the medium-length RNAs and miRNAs in clinical samples with the recognition capability of single nucleic acid.

**Supplementary Information:**

The online version contains supplementary material available at 10.1007/s40820-025-01730-3.

## Graphical abstract

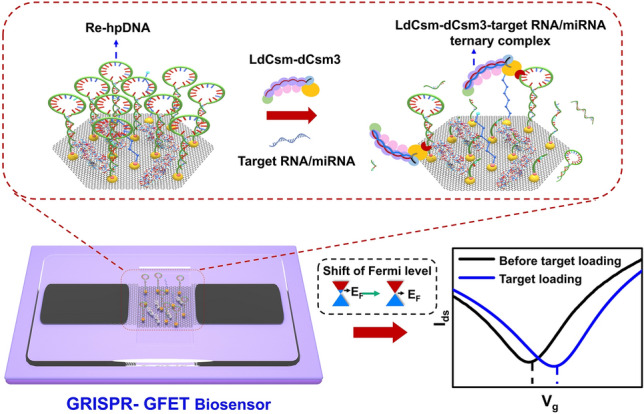

## Introduction

Nucleic acid assays are essential for infectious disease diagnosis, genetic disease screening, and early cancer detection. Their sensitivity, specificity, and detection speed directly impact diagnostic accuracy, treatment efficacy, and public health security [1–4]. Rapid and precise nucleic acid detection is especially critical in contexts such as infectious disease outbreaks and personalized medicine. In recent years, recent progress in molecular biology and nanotechnology has accelerated the development of nucleic acid detection technologies, ranging from enzymatic amplification methods such as quantitative polymerase chain reaction (qPCR) and loop-mediated isothermal amplification (LAMP) [5–7] to enzyme-free amplification strategies like hybridization chain reaction and catalytic hairpin assembly [8–10], more recently, amplification-free direct detection methods [11–13]. Amplification-free strategies, though avoiding amplification-related contamination, are still challenging to detect ultra-low nucleic acid concentration [14, 15]. Thus, there is an urgent need for nucleic acid detection methods that integrate high sensitivity, efficiency, low cost, and operational simplicity to enhance their practical application in molecular diagnostics.

Recently, clustered regularly interspaced short palindromic repeats (CRISPR) and CRISPR-associated (Cas) systems have attracted significant academic and commercial interest for the development of CRISPR-based molecular diagnostics, owing to their exceptional nucleic acid-targeting capabilities [16–19]. In a typical type II CRISPR system, Cas9 proteins cleave double-stranded DNA (dsDNA) under the guidance of CRISPR RNA (crRNA). However, their strict dependence on protospacer adjacent motif recognition limits their applicability in nucleic acid detection [20, 21]. In contrast, type V (Cas12) and type VI (Cas13) CRISPR systems facilitate signal amplification through a unique trans-cleavage mechanism, wherein activation by the target DNA or RNA induces nonspecific cleavage of surrounding DNA or RNA. This process operates in a multiple flip-flop mode, enabling rapid sensor signal generation [22, 23]. However, it also leads to the degradation of the target DNA or RNA, causing the inactivation of Cas12a and Cas13a, thereby preventing sustained trans-cleavage activity [24]. Unlike single Cas protein effectors, the type III CRISPR-Cas10 system features a target RNA-dependent immune mechanism mediated by a multi-subunit effector complex [25–27], offering improved stability at room temperature [28]. More importantly, similar to the type VI system, it specifically recognizes RNA while simultaneously activating the HD deoxyribonuclease (DNase) domain within the Cas10 subunit, facilitating the trans-cleavage of ssDNA [29]. This property has recently shown great potential for nucleic acid detection. Recent advancements have integrated CRISPR systems with electrochemical and optical sensors to develop various amplification-free nucleic acid detection strategies (Table S1) [30–34]. While these CRISPR-based sensors exhibit high sensitivity and specificity, several challenges remain. For instance, electrochemical and optical detection methods often require complex device designs, multi-step reactions, or specific signaling molecules, limiting their practicality. Additionally, conventional optical detection devices are typically bulky and expensive, making them unsuitable for point-of-care (POC) and on-site testing applications.

Field-effect transistors (FETs) are expected to provide more opportunities for the detection of biomolecules due to their richness in analyzable signals, low cost, low power consumption, small size, and good compatibility with integrated circuits [35–39]. In recent years, graphene field-effect transistor (GFET) biosensors have attracted much attention for rapid analysis of nucleic acid information and high-precision detection of various diseases by virtue of the advantages of graphene’s ultrathin sensing layer, high charge carrier mobility, and good biocompatibility [40–43]. However, conventional GFET-based biosensors for nucleic acid detection rarely achieve a limit of detection (LOD) of 10^−16^ M in large amounts of buffer or dilute biological fluids [15, 41, 44, 45]. Numerous efforts have been explored to improve the sensitivity and LOD of GFET-based biosensors for nucleic acid detection, including the development of probes with different structures (e.g., Y-shaped DNA dual-probe [46]), the design of molecular electromechanical systems (combining self-assembled stiff tetrahedral double-stranded DNA structure with flexible single-stranded DNA cantilever [47]), and the combination of GFET with other advanced biotechnologies, such as LAMP [48, 49]. Although these methods achieve highly sensitive nucleic acid detection, they are designed for the detection of only one type of nucleic acid sample and lack platform versatility. Therefore, there is an urgent need to develop a simple, rapid, sensitive, and versatile nucleic acid detection platform to overcome the limitations of existing strategies.

**Fig. 1:**
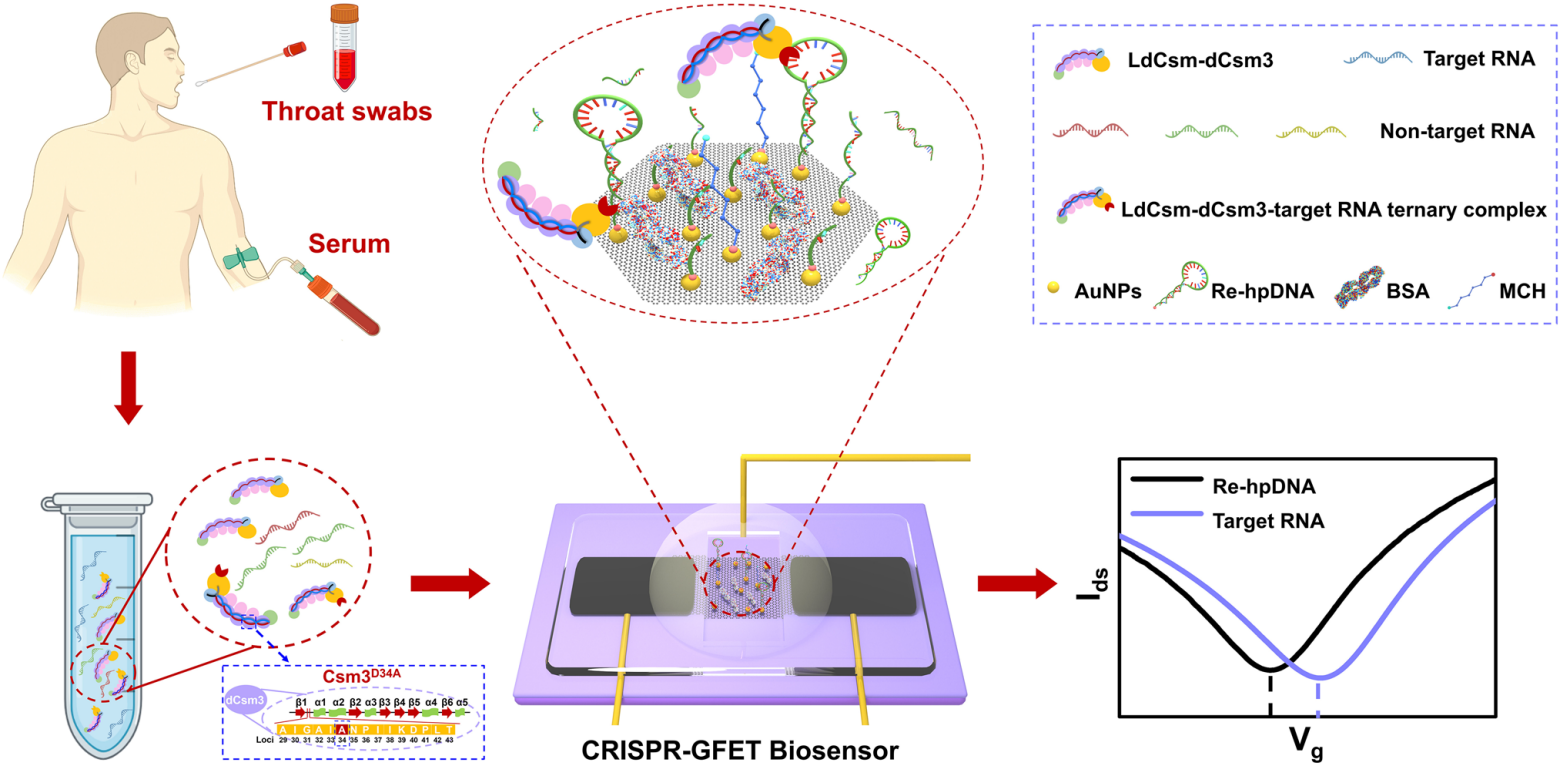
Schematic diagram of the sampling, detection, and working principle of CRISPR-GFET for amplification-free and ultrasensitive detection of RNA samples. A thiolated hairpin DNA reporter (re-hpDNA) is immobilized on the surface of the GFET via Au–S bonds to form the CRISPR-GFET chip. When serum or throat swab samples are mixed with the CRISPR-Cas10 effector complex (LdCsm-dCsm3), the presence of the target RNA activates its Cas10 DNase, leading to massive cleavage and detachment of the immobilized re-hpDNA from the graphene surface, which results in a rightward shift of the Dirac point. Importantly, the dCsm3 (Csm3^D34A^) mutation of the LdCsm effector complex prevents it from degrading the target RNA, thereby maintaining Cas10 DNase activity for continued cleavage of the DNA reporter

Here, we report a cooperative system based on CRISPR-Cas10 with GFETs (CRISPR-GFET), which creates a generalized platform for the direct detection of RNA and miRNA (Fig. 1). On the one hand, based on a novel cleavage sensing mechanism, we introduce a mutation (Csm3^D34A^) in the type III-A CRISPR-Cas10 effector complex to prevent the degradation of target RNA, thus maintaining the activity of Cas10 DNase to continuously cleave the ssDNA reporter, and avoiding a decrease in detection sensitivity due to a decrease in the concentration of target RNA. On the other hand, utilizing high charge density hairpin DNA on GFET channel as a reporter enhances the detection signal. The CRISPR-GFET is able to achieve label-free, amplification-free, highly sensitive, and specific RNA detection. In addition, due to the programmability of CRISPR-Cas10 system, the platform is capable of detecting not only medium-length RNAs, but also miRNAs, with detection limits as low as 214 and 427 aM, respectively. The CRISPR-GFET sensor exhibits excellent immunity to interference in both throat swabs and serum samples, with recoveries ranging from 81.68% to 98.81% in throat swabs, showing good accuracy. In addition, from the perspective of clinical feasibility, we demonstrate that the biosensor can effectively differentiate between healthy individuals and breast cancer patients without the need for extraction, purification, and amplification processes, thereby shortening the detection time and avoiding the risk of false positives caused by nucleic acid amplification and cross-contamination. The platform transcends the limitations of conventional receptor-modified field-effect transistors and is expected to be a versatile and scalable diagnostic toolbox with great potential in molecular diagnostic applications.

## Experimental Section

### Construction of LdCsm Effector Complexes

*Lactobacillus delbrueckii* subsp. *bulgaricus* type III-A (Ld) Csm effector complexes were prepared according to our previous report [25]. Briefly, we first constructed artificial mini-CRISPR plasmid carrying multiple copies of Ld repeat and spacer. Specifically, fusion PCR amplification was performed using primers (RNA-F/R, miR-155-F/R, miRNA-A-5U-F/R) to generate multiple copies of Ld repeat + spacer, yielding the artificial mini-CRISPR fragments. Mini-CRISPR fragments of approximately 1 kb were recovered from agarose gels. Then, the pUCE plasmid was linearized with *Eco*RI and *Sal*I and then amplified with primers pUCE-repeat-F/R. In this way, 5’ half of Ld repeat was added to one end of the pUCE fragment (cut by *Sal*I), whereas the 3’ half of Ld repeat was added to the other end of the pUCE fragment (cut by *Eco*RI). The artificial mini-CRISPR fragment was then ligated to the pUCE fragment containing Ld repeat segments at both ends by Gibson assembly to obtain a pUCE-S-RNA/pUCE-S-miRNA-155/pUCE-S-miRNA-155-A-5U plasmid that can be used to specifically express crRNA targeting RNA/miRNA-155.

The LdCsm effector complex was then purified from *E. coli*. Strains expressing LdCsm effectors were generated by transforming plasmids pUCE-X, pET30a-Csm2, and p15AIE-Cas-dCsm3 into *E. coli* BL21(DE3), where the mutant p15AIE-Cas-dCsm3 plasmid prepared by the splicing overlapping extension PCR protocol previously reported [25, 50]. Single colony was inoculated into 20 mL of LB medium containing ampicillin, kanamycin, and chloramphenicol and grown overnight at 37 °C and 220 rpm. Afterward, 10 mL of the overnight culture was added to 1 L of TB medium and grown under the same growth conditions until the mid-log phase was reached (OD_600_ = 0.8). Subsequently, 0.3 mM IPTG was added and the culture was incubated at 25 °C, 180 rpm for 16 h to induce LdCsm production. Cells were collected by centrifugation at 5000 rpm for 5 min and then resuspended in 50 mL of buffer A. The cell suspension was processed by French press and cell debris was removed by centrifugation at 10,000 rpm for 1 h at 4 °C. Then, on a HisTrap affinity column, the LdCsm complex was collected and it was eluted with buffer B. The product was further purified by Superdex 200 gel filtration using chromatography buffer. Detailed purification process refers to reference [28].

### Fabrication of CRISPR-GFET Biosensors

The production of GFET devices was performed according to our previous reports [51]. Briefly, the GFET was constructed by first inducing two parallel highly conductive laser-induced graphene (LIG) films on PI using a laser and exfoliating them from the PI substrate using PDMS with a cell in the center to form LIG/PDMS electrodes, and then aligning and bonding the LIG/PDMS electrodes to graphene/substrate. Subsequently, AuNPs were spontaneously deposited on the graphene surface, where AuNPs were synthesized by hydrothermal method. Specifically, AuNPs were synthesized by heating a 0.01% aqueous solution of chloroauric acid (HAuCl_4_) to boiling, then adding 1% sodium citrate, stirring, and heating for about 3 min. The resulting AuNPs-modified GFET chips were incubated overnight with 5 µM of TCEP pretreated thiolated DNA reporter (reDNA) including linear DNA reporter (re-lDNA) and hairpin DNA reporter (re-hpDNA). After washing, the reDNA-modified devices underwent a blocking process with 2 mM MCH as well as 1% BSA. Then, the final CRISPR-GFET biosensor was obtained and could be used for subsequent experiments.

The preparation process of CRISPR chips for electrochemical validation and solid-phase fluorescence experiments is similar to that of CRISPR-GFET biosensors, but instead of GFETs, they use graphene electrodes and slides as substrate, respectively.

### CRISPR-GFET for Detection of Medium-Length RNA and miRNA

First, the CRISPR reaction mixture was prepared by combining 1 µL of RNA/miRNA sample, 1 µL of 10 × cleavage buffer, and 1 µL of effector complex with 7 µL of DEPC-treated water. This resulted in a CRISPR reaction system with a final concentration of 1 × cleavage buffer, 20 nM effector complex, and varying RNA/miRNA concentrations, with a dilution factor of 0.1 for the RNA/miRNA sample. The CRISPR-GFET chip was then incubated with this mixture at 37 °C for 45 min. Following incubation, the solution cell was rinsed five times with 500 µL of DEPC-treated water to remove reDNA fragments generated by cleavage on the AuNP surface and to eliminate nonspecifically adsorbed residues on the graphene surface, thereby minimizing false-positive signals. In order to explore the sensitivity, specificity, interference resistance, and practical application of biosensors, the experiments were performed using different RNA/miRNA samples, including different concentrations of target RNA (RNA, miRNA-155), noncomplementary RNA/miRNA (RNA-1, RNA-2, RNA-3, miRNA-4484, miRNA-4732, miRNA-126), base mismatch miRNAs (miRNA-155-4, miRNA-155-16, miRNA-155-3-4, miRNA-155-7-8, miRNA-155-1~3, miRNA-155-4~6, miRNA-155-1~4, miRNA-155-5~8) and serum samples from healthy individuals and breast cancer patients. In addition, the anti-interference ability was validated using pharyngeal swab solution and serum from healthy individuals with the addition of RNA and miRNA-155, respectively.

Details on the experimental materials and methods, including “Blood sample collection,” “Cleavage of fluorescent DNA reporter by LdCsm and LdCsm-dCsm3,” “Gel electrophoresis for evaluating in vitro hybridization feasibility,” “RT-PCR for RNA detection,” as well as experimental characterization and measurement, are provided in the Supporting Information.

## Results

### Design and Efficiency of the CRISPR-Cas10 System for RNA Detection

**Fig. 2:**
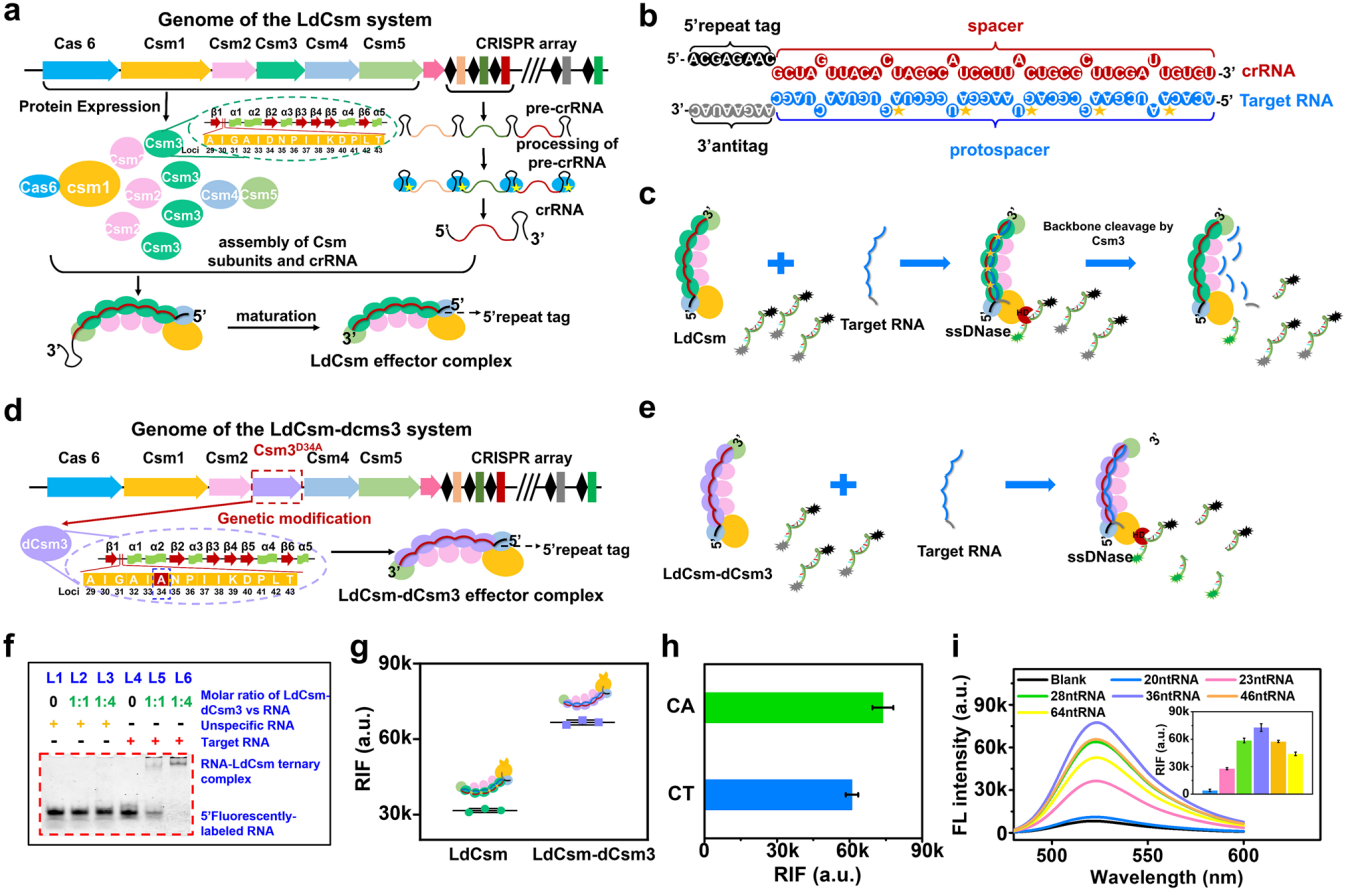
Design and RNA detection efficiency of the CRISPR-Cas10 system. Schematic of the preparation and structure of **a** LdCsm and **d** LdCsm-dCsm3 effector RNP complex. The green and purple dashed ellipse boxes are the structures of Csm3 and dCsm3, respectively. **b** Base complementary pairing between target RNA and crRNA in the LdCsm/LdCsm-dCsm3 RNP complex. Schematic diagram of **c** LdCsm and **e** LdCsm-dCsm3 specific recognition of target RNA and trans-cleavage of fluorescence-quenched ssDNA reporter. **f** Non-denaturing polyacrylamide gel electrophoretic analysis of the hybridization between target RNA and LdCsm-dCsm3 RNP. “+” indicates presence and “−” indicates absence. **g** Comparison of relative fluorescence intensity (RFI) released by cleavage of FQ-fCA by activated LdCsm and LdCsm-dCsm3 RNP. **h** Comparison of RFI released by cleavage of FQ-fCT and FQ-fCA by activated LdCsm-dCsm3 RNP. **i** Fluorescence spectra and comparison of RFI released by cleavage of FQ-fCA upon binding of LdCsm-dCsm3 to target RNAs of different lengths. **g**, **h**, and **i** were assessed by the fluorescence spectrograms in Figs. S1, S2, and S3, respectively

The type III-A Cas gene of *L. delbrueckii* (Ld Cas module) was cloned into a plasmid expression vector, while a mini-CRISPR array containing spacer designed with the target gene was cloned into another plasmid vector. After both were transformed into *E. coli* cells, the plasmid vector expression produced Cas protein and pre-crRNA transcripts and eventually formed the LdCsm ribonucleoprotein (RNP) complex (Fig. 2a). The effector complex’s crRNA recognizes the particular protospacer RNA, to form the target RNA-LdCsm ternary complex (Fig. 2b), which induces a conformational change and activates the Cas10 HD domain, leading to collateral single-stranded DNA (ssDNA) cleavage (Fig. 2c) [25]. The above process is shut down by the enzymatic destruction of the target RNA by the nuclease carried in the large backbone subunit (Csm3), making the process of trans-cleavage of ssDNA unsustainable for long periods of time. Mutation of the 34th amino acid of Csm3 from aspartic acid to alanine was confirmed to shut down the target RNA cleavage activity, generating the LdCsm-dCsm3 effector complex (Fig. 2d) [25]. In theory, this effector complex allows the trans-cleavage process to not be terminated due to RNA cleavage, permitting continuous shearing of ssDNA (Fig. 2e).

To achieve ssDNA cleavage, it is first necessary to ensure that the effector complexes are able to specifically recognize and bind to target RNAs. Therefore, we utilized non-denaturing polyacrylamide gel electrophoresis to assess the binding of LdCsm-dCsm3 complexes to target RNAs. As shown in Fig. 2f, all lanes exhibited clear fluorescent bands, and the migration rate of the target RNA was significantly reduced compared to that of the nontarget RNA. This reduction was attributed to the hybridization of the target RNA with the LdCsm-dCsm3 complex, forming a stable ternary complex that affected RNA migration behavior. Additionally, when the concentration of LdCsm-dCsm3 was increased from 50 to 200 nM, target RNA was almost completely bound to LdCsm-dCsm3 complex. These results validate that the LdCsm-dCsm3 complex is highly specific and can stably hybridize with target RNA.

Further, to assess the cleavage activity of LdCsm and LdCsm-dCsm3 complexes on ssDNA, 5’-FAM- and 3’-BHQ-labeled full CA (FQ-fCA) reporters were introduced in the cleavage reaction. Fluorescence spectroscopy results showed that Cas 10 HD-nucleases could efficiently cleave FQ-ssDNA and release fluorescent signals when LdCsm and LdCsm-dCsm3 specifically recognized the target RNA (Fig. S1). In addition, the activated LdCsm-dCsm3 RNP released a significantly higher relative fluorescence intensity (RIF) than the activated LdCsm RNP (Fig. 2g), indicating that the dCsm3 mutation of LdCsm RNP effector achieved ssDNA circular shearing and functioned as signal self-amplification.

Although the ssDNA reporter plays a role in RNA detection based on the LdCsm-dCsm3 system, the nucleotide cleavage preference of the LdCsm-dCsm3 system remains to be revealed. Previous studies have recorded the primary cleavage sites of the LdCsm system on ssDNA reporters with different sequences, suggesting that the preferred cleavage sites may include CT and CA [28]. However, this speculation has not been systematically validated. Therefore, an additional DNA of 5’-FAM- and 3’-BHQ markers full CT (FQ-fCT) was introduced together with FQ-fCA as reporters to investigate the cleavage preference of the LdCsm-dCsm3 system. As shown in Fig. 2h, the fluorescence signals released by cleavage of FQ-fCA reporter were significantly higher than that of FQ-fCT reporters, indicating that the activated LdCsm-dCsm3 RNP cleaved the FQ-fCA reporter more efficiently and that di-ribonucleotide cleavage of CA by the LdCsm-dCsm3 RNP was preferential.

Previous studies have shown that the target RNA must meet two conditions in order to activate the Cas10 DNase activity of LdCsm-dCsm3 effector complex: (i) it must carry a sequence complementary to the LdCsm-dCsm3 crRNA spacer region (named protospacer); (ii) its 3’-protospacer flanking sequence (3’-antitag) must not match the LdCsm-dCsm3-crRNA’s 5’-repeat tag [25]. To explore the effect of target RNA length on the extent of LdCsm-dCsm3 RNP activation, we used RNAs with mismatched 5’-repeats tag and different lengths of the protospacers (64 nt RNA, 46 nt RNA, 36 nt RNA, 28 nt RNA, 23 nt RNA, 20 nt RNA, their matches with crRNA are shown in Fig. S3) to activate LdCsm-dCsm3 effector complex and assessed the activity by RFI from cleaved FQ-fCA reporters. As shown in Fig. 2i, the fluorescence signal generated by the cleavage reporter first increased and then decreased with increase in length of the target RNA bases over the length range of 20–64 nt. The strongest RFI was generated by the activation of the LdCsm-dCsm3 RNP by 36 nt RNA, while for RNAs less than 28 nt in length, the RFI was significantly diminished (inset of Fig. 2i). These results suggest that in the presence of 5’-repeat tag mismatches, target RNAs larger than 20 nt all activate the LdCsm-dCsm3 effector complex to varying degrees, with the highest activation occurring at 36 nt. Further reduction or addition of nucleotides would hinder the activation of LdCsm-dCsm3 effector complex, and the activation effect of RNAs smaller than 28 nt would be strongly hindered.

In a word, the CRISPR-Cas10 system can recognize target RNAs of varying lengths and activate the Cas10 HD domain to different extents. Activated Cas10 undergoes “collateral cleavage,” efficiently cleaving nearby ssDNA molecules with a preference for cleaving CA dinucleotides. Additionally, the LdCsm-dCsm3 effector complex, due to a mutation in the 34th amino acid of Csm3, prevents degradation of the target RNA, allowing for signal amplification through continuous ssDNA cleavage. The LdCsm-dCsm3 effector complex is impressive in the programmability for nucleic acid recognition, the ssDNA cleavage and the sequential cleavage processes, making it promising for RNA detection applications.

### Construction, Verification, and Optimization of CRISPR-GFET Biosensor

**Fig. 3:**
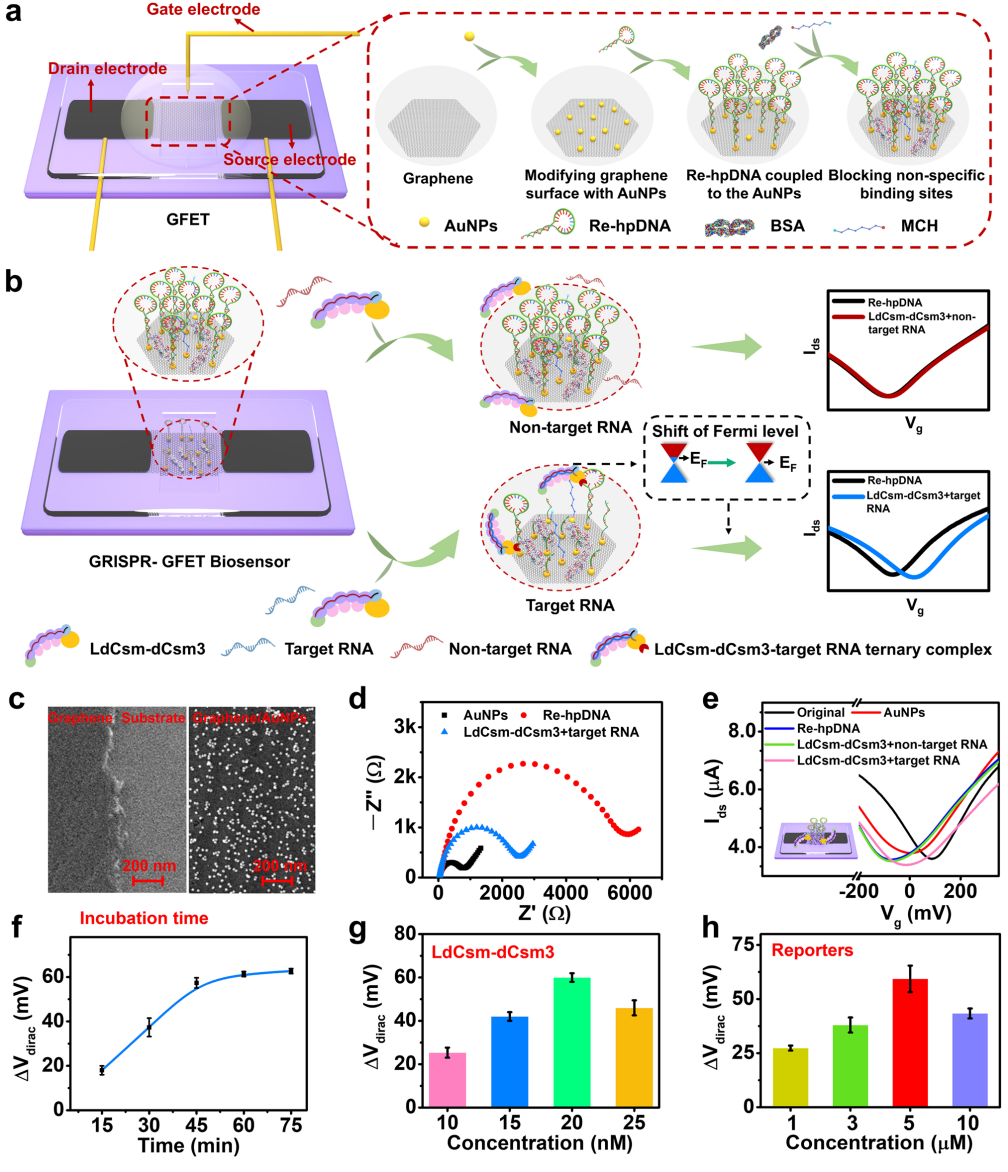
Construction, characterization, verification, and optimization of a CRISPR-GFET biosensor. Schematic diagram of **a** fabrication process and **b** working mechanism of the CRISPR-GFET biosensor. **c** SEM images after deposition of AuNPs on the graphene surface. **d** EIS of the graphene working electrode after AuNP deposition, re-hpDNA modification, and incubation with LdCsm-dCsm3 mixture containing the target RNA. **e** Stepwise transfer curves of the CRISPR-GFET biosensor before and after AuNP deposition, re-hpDNA modification, and incubation with LdCsm-dCsm3 mixture containing nontarget RNA and target RNA. Optimization of the biosensor for **f** reaction time, the concentration of **g** LdCsm-dCsm3 effect complexes and **h** re-hpDNA, assessed by the transfer curves in Figs. S7, S8 and S9, respectively

GFETs are excellent biosensing platforms that provide rich analytical signals, high sensitivity, and low cost by combining the advantages of graphene and field-effect transistors. In this study, we successfully fabricated van der Waals-contacted GFETs by leveraging the unique properties of LIG, including a simple fabrication process, excellent electrical conductivity, the ability to form van der Waals contacts with 2D materials, and abundant oxygen-containing functional groups [52]. Specifically, the device was fabricated using PDMS/LIG transfer electrodes and a one-step, straightforward pasting method. Raman spectroscopy, optical imaging, and *I*–*V* measurements demonstrated that the LIG electrodes exhibited excellent conductivity and uniformity (Fig. S4a, b). By aligning PDMS/LIG electrodes and graphene/substrate microscopically, we successfully obtained van der Waals-contacted GFETs, with optical images shown in Fig. S4c. The PDMS/LIG transfer electrode enabled the formation of a clean van der Waals contact between the electrode and the semiconductor layer. Additionally, the oxygen-containing functional groups in the LIG contribute to the doping of graphene, reducing the contact resistance between the electrode and graphene [51]. As a result, the device demonstrated excellent electrical properties, with hole and electron mobilities (*μ*_h_ and *μ*_e_) of 3584 and 3132 cm^2^ V^−1^ s^−1^, respectively (Fig. S4d). By combining this GFET with the CRISPR-Cas10 system, we developed a CRISPR-GFET biosensor for RNA detection. Its specific construction steps and sensing mechanism are shown in Fig. 3a, b. The AuNP-modified GFET chip was first prepared and the sulfhydryl hairpin DNA reporter (re-hpDNA) was immobilized through Au–S bonding on the AuNP surface, and then the nonspecific sites were blocked with BSA and MCH to construct the CRISPR-GFET substrate chip. In this process, the negative charge enriched in the DNA phosphate carbon backbone shifts the graphene Fermi energy level upward through charge transfer, i.e., graphene achieves *n*-type doping, resulting in a negative shift of the GFET Dirac point. Since nontargeted molecules do not have predesigned targeting sites for CRISPR, they cannot activate the LdCsm-dCsm3 RNP to cleave the immobilized reporter and do not cause Dirac point shift. In contrast, the presence of target RNA activates the LdCsm-dCsm3 RNP, making the immobilized hairpin reporter is bulk cleaved and left the graphene surface. During the above process, the electron doping of graphene by the reporter decays sharply and the graphene Fermi energy shifts downward, which in turn induces a backward of the Dirac point (i.e., the positive shift corresponds to the *p*-type doping of graphene). In summary, the Dirac point displacement (Δ*V*_dirac_, the shift in *V*_dirac_ after incubation with activated LdCsm-dCsm3 RNP relative to the *V*_dirac_ of DNA reporters) induced by the target RNA-activated LdCsm-dCsm3 RNP cleavage reporter is an indicator of the detection of the target RNA by the CRISPR-GFET cooperative system.

Figures 3c and S5a show the scanning electron microscopy (SEM) and energy-dispersive X-ray spectroscopy (EDS) analysis, respectively. The results demonstrated that high-density AuNPs with a diameter of about 10 nm were formed on the graphene surface. In addition, XPS detected Au 4*f* characteristic peaks at Au 4*f*_7/2_ (83.7 eV) and Au 4*f*_5/2_ (~ 87.4 eV), further confirming the successful modification of AuNPs (Fig. S5c). These AuNPs possess large and specific surface, which provides more space for the cleavage activity of activated LdCsm-dCsm3 RNP, thus reducing its possible spatial resistance on the solid-phase chip surface [53]. The increase in fluorescence intensity after immobilization of FAM-labeled thiolated linear DNA reporter (re-FAM-lDNA) in the fluorescence images and the heat map confirms the feasibility of the immobilized reDNA method by Au–S bonding (Fig. S5b). Subsequently, the immobilization of re-hpDNA was further investigated using electrochemical impedance spectroscopy (EIS). The semicircle diameter of the EIS curve is equal to the charge transfer resistance (*R*_ct_). As shown in Fig. 3d, the immobilization of the hpDNA reporter onto the AuNP surface led to electrostatic repulsion between the negatively charged re-hpDNA backbone and the electroactive species [Fe(CN)_6_]^3−^^/^^4−^, hindering its diffusion to the electrode surface. This resulted in reduced electron transfer and a significant increase in *R*_ct_, further confirming successful re-hpDNA immobilization. Furthermore, the detection of characteristic S 2*p* (162.2 eV), P 2*p* (134.2 eV), and N 1*s* (400.0 eV) signals in the XPS spectra provided additional evidence for effective re-hpDNA immobilization via Au–S bonds (Fig. S5d–f) [53]. The above results demonstrate the successful construction of a CRISPR-GFET substrate chip using gold nanoparticles as a medium to immobilize re-hpDNA.

Unlike RNA liquid-phase assays based on CRISPR systems, the cleavage reaction of the CRISPR-GFET sensor occurs at the solid–liquid interface. As shown in Fig. S5b, in the presence of RNA, the Cas10 HD nuclease activity of the LdCsm-dCsm3 complex was activated, leading to cleavage of re-FAM-lDNA and a significant decrease in fluorescence on the chip, demonstrating the feasibility of the cleavage at the solid–liquid interface. Additionally, EIS showed a notable decrease in *R*_ct_ after incubation with activated LdCsm-dCsm3 RNP on the graphene working electrode modified with re-hpDNA (Fig. 3d), further confirming the feasibility of cleaving hpDNA reporters by LdCsm-dCsm3 RNP from the solid substrate surface. The construction and feasibility of the CRISPR-GFET biosensor was further demonstrated by the *I*_d_–*V*_g_ transfer curve (Fig. 3e). Specifically, due to n-doping after AuNP deposition, *V*_dirac_ shifts in the negative direction (red line). And the negatively charged phosphorus–carbon backbone of re-hpDNA further causes *V*_dirac_ to shift leftward (blue line). In the absence of target RNA, the inactivated Cas10 in LdCsm–dCsm3 effector complex is unable to cleave re-hpDNA, resulting in almost no *V*_dirac_ shift (green line). However, when LdCsm-dCsm3 effector complex binds to the target RNA, the Cas10 HD domain is activated, leading to the cleavage of immobilized re-hpDNA and causing *V*_dirac_ to shift positively (pink line).

It is worth noting that during the operation of the CRISPR-GFET biosensor described above, the re-hpDNA on the surface of the CRISPR-GFET biosensor is shielded by ions due to the presence of moving ions in the PBS test solution [54], which is known as the Debye shielding effect affecting the GFET biosensor sensitivity [54, 55]. To circumvent the Debye screening effect, the ionic strength of the test solution can be reduced to increase the Debye length (λ_D_). As shown in Fig. S6d, the electrical response of GFET to the re-hpDNA gradually increases with decrease in PBS concentration, and the growth of its response signal slowed down after 20 µM PBS (λ_D_∼15.5 nm, as calculated from Eq. S1). In addition, the concentration of PBS inevitably affects the electrical properties of the device (Fig. S6e) and may cause false positives [54], so we chose 20 μM PBS for our experiments.

CRISPR reaction conditions, such as temperature, concentration of metal ions, incubation time, concentration of LdCsm-dCsm3 effector complex, and concentration of DNA reporter, may affect the performance of CRISPR-GFET in nucleic acid detection. Among them, temperature, metal ion concentration creates the reaction environment for CRISPR, similar to the liquid-phase reaction. Previous experiments and reports suggest that the optimal temperature is 37 °C [28]. While CRISPR-Cas10 was activated by 10 mM MgCl_2_, the addition of 50 mM KCl further increased the DNA cleavage activity [25]. Therefore, we chosed a reaction temperature of 37 °C and used a cleavage buffer containing 10 mM MgCl_2_ and 50 mM KCl, and optimized the detection of RNA by varying three operating conditions: incubation time, LdCsm-dCsm3 concentration, and DNA reporter concentration of the CRISPR-GFET platform.

We first investigated the incubation time of the assay, as increasing the incubation time would allow for more shearing events, producing a greater signal response. As displayed in Fig. 3f, the shift of *V*_dirac_ gradually increased as the incubation time was increased to 75 min, and after 45 min, the rate of increase in* V*_dirac_ shift slowed down as the incubation time increased. From the point of view of future point-of-care applications, a highly sensitive and rapid detection is required. Considering that within 45 min for the CRISPR-GFET platform provides sufficient Δ*V*_dirac_ and is both time-efficient, the 45 min incubation time was used for further experimental setup.

The LdCsm-dCsm3 effector complex directly controls biosensing efficiency by controlling two key elements: the capture of the target RNA and the trans-cleavage of the DNA reporter. For low concentrations of LdCsm-dCsm3 RNP, cleavage of the re-hpDNA will be limited by the LdCsm-dCsm3 RNP available for the reaction. However, for high concentrations of LdCsm-dCsm3 RNP, its large size reduces the binding rate to the target RNA as well as the diffusion rate to the graphene surface, ultimately reducing the target RNA-induced trans-cleavage events and thus hindering the analytical performance. Therefore, we varied the concentration of the LdCsm-dCsm3 effector complex from 10 to 25 nM to explore the optimal concentration of the LdCsm-dCsm3 effector complex. As the effector complex concentration increased, the response signal (Δ*V*_dirac_) of CRISPR-GFET first increased and then decreased, and the response reached a maximum at 20 nM, indicating that the optimal LdCsm-dCsm3 concentration for the CRISPR-GFET collaborative system is 20 nM (Fig. 3g).

The concentration of re-hpDNA immobilized on GFET determines the upper limit of trans-cleavage of activated LdCsm-dCsm3 RNP. As the coverage of re-hpDNA increases, it leads to a larger detection range and higher sensitivity. However, excessively high re-hpDNA coverage may result in spatial site blocking, preventing LdCsm-dCsm3 RNP from approaching the reporter fixed on the graphene surface, thus inhibiting the cleavage of immobilized re-hpDNA. To balance the two, we immobilized different concentrations of re-hpDNA on GFET to explore the optimal conditions for the CRISPR-GFET biosensor. As shown in Fig. 3h, the response signal (*V*_dirac_) of the biosensor to the target RNA increased and then decreased in the concentration range from 1 to 10 µM, and the optimal concentration of re-hpDNA was 5 µM.

### Sensing Performance of the CRISPR-GFET Biosensor

**Fig. 4:**
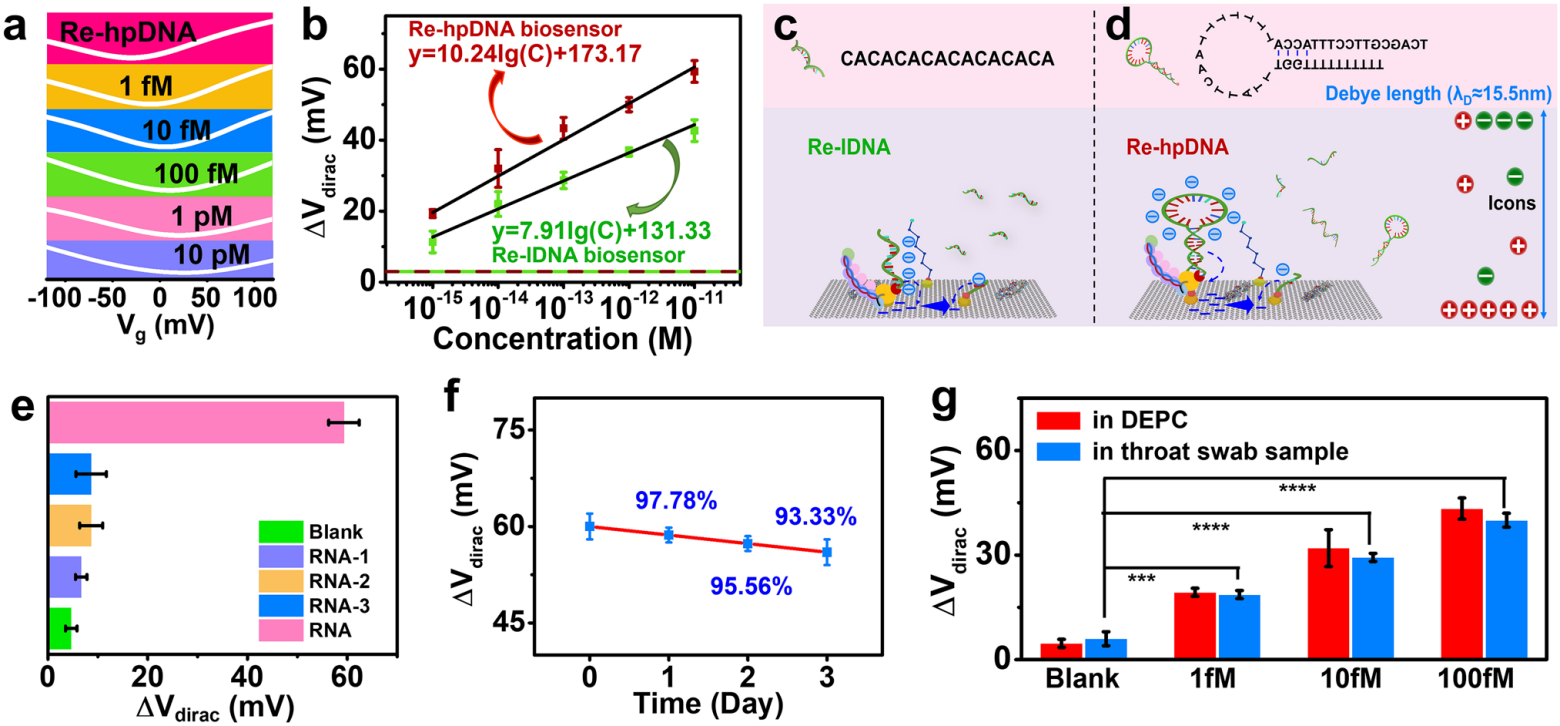
Sensing performance of CRISPR-GFET biosensor. **a** Transfer curves of the hairpin CRISPR-GFET biosensor after incubation with LdCsm-dCsm3 mixture containing different concentrations of RNA. **b** Calibration curves between the Δ*V*_Dirac_ and different levels of RNA for the re-hpDNA biosensor and the re-lDNA biosensor, where the red and green dashed lines are the triple standard deviation of the blank samples of the re-hpDNA and re-lDNA biosensors, respectively. Sensing mechanism of **c** re-lDNA biosensor and **d** re-hpDNA biosensor, where the upper panel shows the structures of re-lDNA and re-hpDNA, respectively. **e** Sensing signal of CRISPR-GFET biosensor for nonspecific RNA and target RNA. **f** Stability of CRISPR-GFET biosensor. **g** Comparison of the electrical response of the CRISPR-GFET biosensor to target RNA in DEPC and throat swabs. **b**, **e**, **f**, and **g** were assessed by the transfer curves in Figs. S10, S11 and S12, S13, S15, S17 as well as S10, S11 and S19, respectively

RNA detection was achieved using CRISPR-GFET under optimized assay conditions (37 °C, 45 min incubation time, 20 nM LdCsm-dCsm3). Specifically, the LdCsm-dCsm3 mixture consisting of the LdCsm-dCsm3 effector complex, cleavage buffer, RNase inhibitor, and different concentrations (1 fM-10 pM) of target RNA was sequentially introduced into the device. As shown in Fig. 4a, the *V*_dirac_ showed a gradual positive shift with increase in target RNA concentrations. The signal values corresponding to target RNAs ranging from 1 fM to 10 pM were 19, 32, 43, 50, and 59 mV. The calibration curve in Fig. 4b exhibited good linearity between the target RNA concentration and the logarithm of the response signal with a regression equation of Δ*V*_dirac_ = 10.24 lgC + 173.17 and a correlation coefficient value (*R*^2^) of 0.9930. According to LOD = *3S*_*b*_*/S*, the LOD was as low as 214 aM [56]. Currently, most FET biosensors based on CRISPR systems are constructed with linear DNA reporters (re-lDNA) [40, 57], so we also constructed a CRISPR-GFET biosensor using a re-lDNA and compared it with a re-hpDNA biosensor. The green line in Fig. 4b clearly demonstrates that the re-lDNA biosensor also successfully detected the RNA with a LOD of 274 aM, and the linear equation is expressed as Δ*V*_dirac_ = 7.91 lg*C* + 131.33 with *R*^2^ equal to 0.9888. Its sensitivity was only 77% of that of the re-hpDNA biosensor.

According to the electric double layer (EDL) theory, the Dirac point displacement equation of graphene is $$\Delta {V}_{dirac} =\frac{e\Delta n}{{C}_{T}}$$, where *e*, *Δn*, and *C*_*T*_ represent the elementary charge, graphene carrier density variation, and total gate capacitance, respectively [58]. The *C*_*T*_ of the CRISPR-GFET biosensor is almost constant, while *Δn* is proportional to the change in charge density of the charged material on the graphene surface. As shown in in Fig. S14, the re-hpDNA immobilized in GFET produces a Dirac point shift of approximately 1.53 times that of re-lDNA, indicating that the charge density of re-hpDNA is higher than that of re-lDNA, and implying a larger charge allowed to activate the LdCsm-dCsm3 RNP shear. The optimal cleavage site for the LdCsm-dCsm3-target RNA ternary complex is the CA dinucleotide. Based on the structural maps of re-lDNA and re-hpDNA, with the CA as the primary cleavage site, re-hpDNA loses more of its phosphoribosylated carbon skeleton than re-lDNA (Fig. 4c, d). However, it is worth noting that since the LdCsm-dCsm3-target RNA ternary complex allows indiscriminate cleavage of DNA, cleavage of other sites in addition to the CA site is inevitably accompanied. Even so, through time optimization, essentially complete cleavage of the reporter on the CRISPR-GFET sensing platform can be achieved, i.e., the re-hpDNA biosensor produces a larger *Δn* in the recognition RNA, which leads to a greater positive shift (Fig. 4b). In summary, the designed CRISPR-GFET biosensor has better sensing performance than the conventional re-lDNA CRISPR-GFET biosensor due to the high charge density of re-hpDNA and the novel cleavage sensing mechanism. Furthermore, the CRISPR-GFET-based nucleic acid detection method offers detection time and detection limit that are comparable to or even superior to existing CRISPR-based amplification-free techniques, such as colorimetry [59], electrical [21, 60], SERS [61], fluorescence [28, 62], electrochemiluminescence [63, 64], and electrochemistry [30, 32, 33, 65] methods (Table S1). Additionally, the use of low-cost LIG fabricated via laser direct writing as the electrode not only simplifies the manufacturing process of the CRISPR-GFET biosensor, but also reduces its cost, with each device costing only $1.16 (Table S1). A significant portion of this cost is due to the substrate, and by employing lower-cost alternatives such as polyimide (PI), the overall cost can be further reduced. In conclusion, the CRISPR-GFET biosensor combines a low detection limit, simple fabrication processes, and low cost.

Next, we explored the specificity of our designed CRISPR-GFET biosensor. LdCsm-dCsm3 mixtures without RNA (blank) as well as containing 10 pM of nontarget RNA (RNA-1, RNA-2, RNA-3) and target RNA were incubated on the biosensor, respectively. Figure 4e summarizes the results of three parallel experiments. The Δ*V*_dirac_ values were 5, 7, 9, 9, and 59 mV when interacting with blank, nontarget RNA-1, RNA-2, RNA-3, and target RNA, respectively, where the electrical response of target RNA was six times higher than that of nontarget RNA. This result indicates that our CRISPR-GFET biosensor is able to distinguish between target and nontarget RNAs and has good specificity.

The repeatability and long-term stability of the sensor are critical for ensuring reliable RNA detection. Repeatability reflects the consistency of fabrication and directly impacts the comparability of experimental data, while long-term stability determines the sensor’s practical applicability and operational lifespan. To evaluate reproducibility, six parallel sensors were fabricated and tested for their electrical responses to RNA at concentrations of 1 and 10 pM. The relative standard deviations (RSDs) of the electrical responses were 4.48% and 3.33%, respectively (Fig. S16), demonstrating high consistency and excellent reproducibility across different sensor batches. The long-term stability of the sensor was further evaluated by measuring its response to 10 pM RNA after storage at 4 °C for 0–3 days. As shown in Fig. 4f, the sensors retained approximately 97.78%, 95.55%, and 93.33% of their initial signal after 1, 2, and 3 days of storage, respectively. While a slight decline in signal retention was observed over time, the maximum deviation remained below 7%, confirming the sensor’s outstanding long-term stability.

PCR is widely regarded as the gold standard for RNA detection. To evaluate the accuracy of the CRISPR-GFET biosensor, we performed reverse transcription quantitative PCR (RT-qPCR) on RNA samples and compared the results with those obtained from the CRISPR-GFET. Specifically, we used RT-qPCR to analyze the amplification profiles of various concentrations of RNA (single assay time: 140 min) and generated the corresponding standard curves. Additionally, a mixed solution containing 5 pM of target RNA and 100 pM of nontarget RNA (RNA-1, RNA-2, RNA-3) was analyzed using both RT-qPCR and the CRISPR-GFET biosensor. As shown in Fig. S18, the cycle threshold (Ct) values of the target RNAs exhibited a strong, linear correlation with their concentrations. The Ct values and Dirac point shifts of the mixed solutions were 26.18 and 57.33 mV, respectively, corresponding to target RNA concentrations of 4.329 and 4.870 pM. The recoveries for RT-qPCR and CRISPR-GFET biosensor were 86.58% and 97.40%, with RSDs of 7.60% and 4.03%, respectively (Table S2). Overall, CRISPR-GFET demonstrated better recovery of RNA in the mixed solution compared to RT-qPCR, showing good accuracy. Additionally, the CRISPR-GFET was easier to operate, significantly reduced detection time, and outperformed other CRISPR-based amplification detection methods (Table S1), while also minimizing the risk of false positives caused by sample contamination and nonspecific amplification, thereby enhancing the reliability of the assay.

Finally, to ensure practicality, we evaluated the resistance of the sensor to interference in the biological environment. Briefly, different concentrations of target RNA (1, 10, and 100 fM) was added to negative throat swabs solution and resulting artificial samples were tested by CRISPR-GFET biosensor following the same protocol employed in RNA solutions. We can see that the background value increases and the response signal decrease slightly in the complex throat swab matrix environment (Fig. 4g). This is owing to the presence of multiple components including RNA, DNA, and protein in throat swabs. On one side, the partial matching of some nontarget RNA with the LdCsm-dCsm3 effector complex may activates the RNP to some extent, leading to an increase in the background value; on the other side, too much RNA or DNA interferes with the binding of the LdCsm-dCsm3 effector complex to the target RNA, leading to a decrease in the target response signal. Although the response signal of the CRISPR-GFET sensor was attenuated in the throat swab matrix, it still responded adequately to the target RNA sample, generating a significant signal difference from the background signal (Fig. 4g), indicating that the biosensor has a good immunity to throat swab solutions. The RNA concentration in the spiked samples was back-calibrated using the standard curve in Fig. 4b, and the recoveries and their deviations from the actual spiked concentrations were analyzed. As shown in Table S3, the recoveries of the detected RNA concentrations were 81.68%–98.81%, and the RSD from the spiked RNA concentrations were 3.94%–6.19%, indicating that the CRISPR-GFET biosensor is capable of detecting RNA in simulated throat swab samples with high accuracy and reliability.

### CRISPR-GFET Biosensor for miRNA-155 Detection

To further demonstrate the ability of CRISPR-GFET as a universal platform, this designed biosensor was also used to detect microRNA (miRNA). Breast cancer poses a serious threat to women’s lives and health, as it is the second most frequently diagnosed malignancy among women worldwide and has the second highest mortality rate among cancers. In the long run, the mortality rate of breast cancer can be greatly reduced by early diagnosis of malignant tumors. MiRNAs-155 and their expression levels are associated with breast cancer [66, 67]. Therefore, here we explored the role played by CRISPR-GFET in detecting miRNAs using miRNAs-155 as the target molecule.

**Fig. 5:**
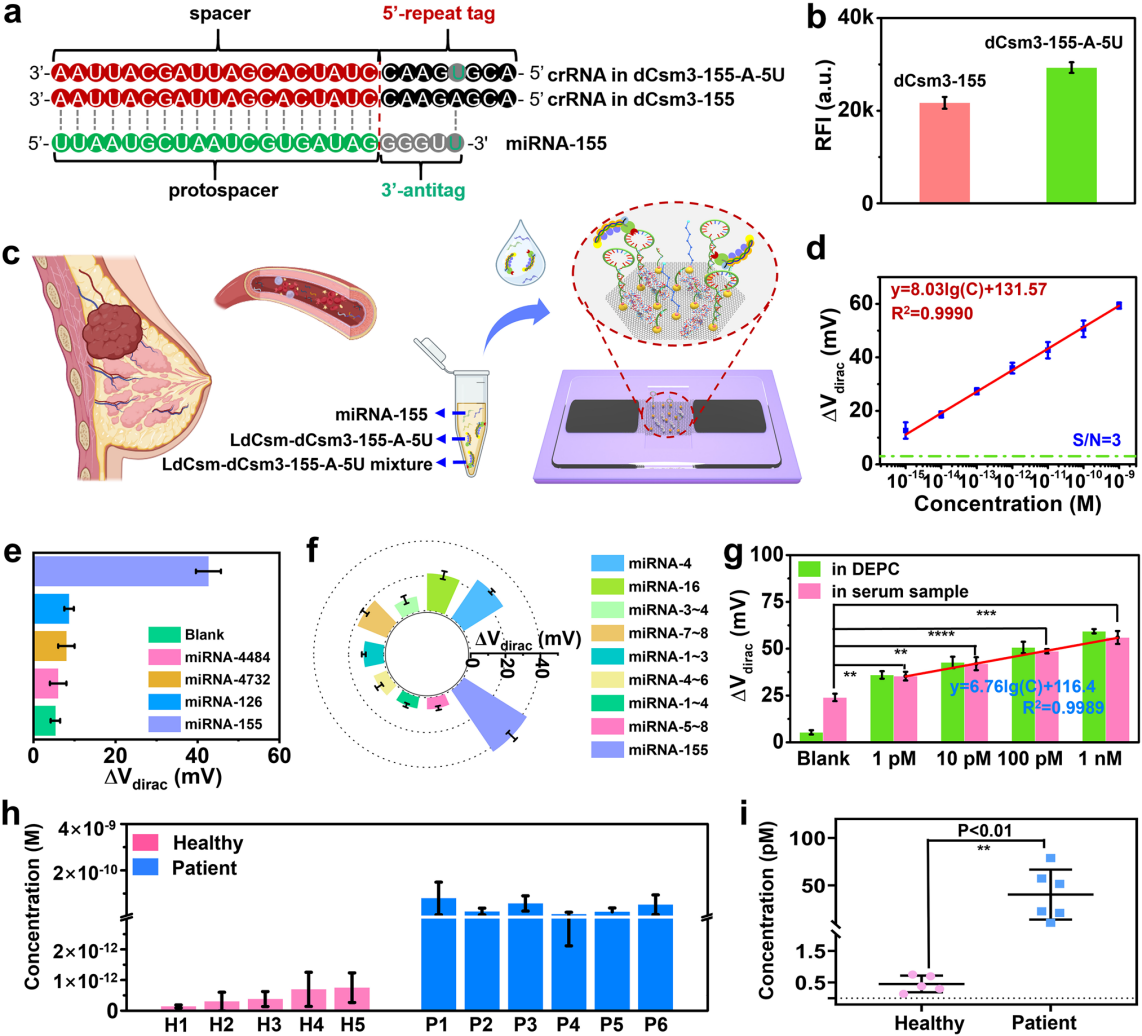
MiRNA-155 detection by the CRISPR-GFET biosensor. **a** Schematic of the crRNAs designed for miRNA-155 detection. Mutated bases in the repeat tags are shown in dark blue. **b** Comparison of the RFI of LdCsm-dCsm3-155 and LdCsm-dCsm3-155-A-5U cleaved FQ-fCA, which was evaluated according to the fluorescence spectra in Fig. S20. **c** Schematic diagram of breast cancer detection. **d** Calibration curves between the Δ*V*_*dirac*_ and different levels of miRNA for the CRISPR-GFET biosensor, where the green dashed lines are the triple standard deviation of the blank samples. **e** Sensing signal of CRISPR-GFET biosensor for nonspecific miRNA and miRNA-155. **f** Sensing signal of CRISPR-GFET biosensor for single-base mismatch miRNAs (miRNA-155-4, miRNA-155-16), two-base mismatch miRNAs (miRNA-155-3-4, miRNA-155-7-8), three-base mismatch miRNAs (miRNA-155-1~3, miRNA-155-4~6), four-base mismatch miRNAs (miRNA-155-1~4, miRNA-155-5~8), and miRNA-155. **g** Comparison of the electrical response of the CRISPR-GFET biosensor to miRNA-155 in DEPC and serum, containing calibration curves between Δ*V*_*dirac*_ of the CRISPR-GFET biosensor and different concentrations of miRNA-155 in serum. **h** The concentration of miRNA-155 in serum samples from healthy individuals and breast cancer patients. **i** Statistical comparisons of concentrations in healthy individuals and breast cancer patients, as assessed by the concentrations in **g**. **d**, **e**, **f**, **g**, and **h** were c the transfer curves in Figs. S21–S23, S24, S26, S27, and S28, as well as S29–S31, respectively

Unlike the medium-length RNA, for the 24 nt miRNA, its 3’ terminal sequences must be directly used as 3’-antitags because the whole miRNA is too short to provide any alternative 3’-antitag, exhibiting a single-base matching between its 3’-antitag and the 5’ repeat tag of the corresponding crRNA (Fig. 5a). Therefore, we chose to introduce a single-base substitution repeat sequence in the LdCsm CRISPR array to generate an exact mismatch between the 5’ repeat tag of crRNA and the 3’-antitag of miRNA-155. Specifically, we introduced a point mutation (A → T) in pUCE-miRNA-A-5U and then generated the LdCsm-dCsm3-155-A-5U complex with the pUCE-miRNA-A-5U plasmid, and the resulting crRNA carrying a 5’ repeat tag completely mismatching with the 3’-antitag of miRNA-155 (Fig. 5a). The specific recognition of miRNA-155 by the LdCsm-dCsm3-155-A-5U complex was first confirmed by the fluorescent signal generated by cleavage of FQ-fCA (Fig. S20). The LdCsm-dCsm3-155-A-5U complex cleaved the reporter ge nerating significantly higher RFI than the LdCsm-dCsm3-155 complex (Fig. 5b), showing that its cleavage efficiency was significantly better than that of LdCsm-dCsm3-155 complex. Therefore, we constructed a CRISPR-GFET-based miRNA biosensor by utilizing the LdCsm-dCsm3-155-A-5U complex, and demonstrated its promising application in early breast cancer screening (Fig. 5c).

First, the sensitivity of the CRISPR-GFET biosensor to miRNA-155 was tested. As shown in Fig. S23c, the Dirac point showed a gradual positive shift with increase in miRNA-155 concentration in the concentration range of 1 fM to 1 nM, corresponding to signal levels of 13, 19, 27, 36, 43, 51, and 59 mV, respectively. A regression equation is obtained as Δ*V*_dirac_ = 8.03lgC + 131.57 (*R*^2^ = 0.9990) with LoD of 427 aM (Fig. 5d).

CRISPR mixtures without miRNA and containing miRNA-4484, miRNA-4732, miRNA-126, and miRNA-155 were introduced into CRISPR-GFET biosensor to observe their specificity for the target miRNAs. Blank, 10 pM miRNA-4484, miRNA-4732, miRNA-126, and miRNA-155 had Δ*V*_dirac_ of 5, 6, 8, 9, and 43 mV, respectively (Fig. 5e). It is clear that the electrical signal generated by incubation with the target miRNA is significantly higher than that of the nonspecific miRNA and blank, indicating a satisfactory specificity of our CRISPR-GFET biosensor. Subsequently, we further examined the sensitivity of the CRISPR-GFET biosensor to base mismatches in the target sequences. These base mismatches included single-base mismatch miRNAs (miRNA-155-4, miRNA-155-16), two-base mismatch miRNAs (miRNA-155-3-4, miRNA-155-7-8), three-base mismatch miRNAs (miRNA-155-1~3, miRNA-155-4~6), and four-base mismatch miRNAs (miRNA-155-1~4, miRNA-155-5~8). These mismatches were located at different positions of the target sequences, and the specific mismatched sequences are shown in Fig. S25 and Table S3. The electrical responses of the CRISPR-GFET biosensor to 10 pM miRNA-155-4, miRNA-155-16, miRNA-155-3-4, miRNA-155-7-8, miRNA-155-1~3, miRNA-155-4~6 miRNA-155–1~4, miRNA-155–5~8 were 27, 21, 9, 18, 11, 8, 7, 7, and 43 mV, respectively (Fig. 5f). Three- and four-base mismatched sequences can be clearly distinguished from fully complementary sequences. However, the biosensor's sensitivity to single- and two-base mismatches was not as high, possibly because these mismatched miRNAs also activate the LdCsm-dCsm3-155-A-5U complex to some extent during matching with crRNAs, thereby leading to reporter cleavage. It is also noteworthy that although both are two-base mismatches, the 3–4 and 7–8 nt mismatch sites differ in the degree of activation of the complex. This difference may be due to the fact that mismatches within the 1–8 nt region of type III systems strongly influence the cleavage or interference activity of DNA in vivo [68–70], where the 3–4 nt position is closer to the center of the core recognition region, making the complexes more sensitive to mismatches at this position. Despite the LdCsm-dCsm3-155-A-5U complex’s insensitivity to single- and two-base mismatches, synthesized mismatched crRNAs are still expected to facilitate the detection of these mismatches [22].

MiRNA-155, an upregulated miRNA, is significantly increased in the serum of breast cancer patients [71, 72]. To make our biosensor more relevant for practical applications, we added miRNA-155 to healthy human serum to assess the ability of the biosensor to interfere with serum samples. As shown in Fig. 5g, in the serum matrix, the background signal of the CRISPR-GFET sensor increases, while the response signal of the target miRNA decreases, and there is a significant signal difference between them. We attribute the signal change of background to the presence of small amounts of miRNA-155 in healthy human serum samples [73], which activates RNP and thus leads to an increased signal. While in serum spiked samples, the interference effect of excess nonspecific RNA dominated, resulting in a decreased target response signal. The above results indicate that the developed CRISPR-GFET sensor has good anti-interference properties for serum samples.

Encouraged by the above anti-interference results, we evaluated the performance of CRISPR-GFET biosensor in potential clinical samples, using samples from healthy individuals and breast cancer patients as control and experimental groups, respectively. More detailed expression levels of miRNA biomarkers in healthy and patient sera and differences between healthy individuals and breast cancer patients are shown in Fig. S32. In order to assess the concentration of miRNA-155 in serum relatively accurately, we used high concentration (1 pM, 10 pM, 100 pM, 1 nM miRNA-155) serum spiked samples to fit a standard curve with their generated electrical signals (Δ*V*_dirac_), which was Δ*V*_dirac_ = 6.67lgC + 116.4 with R^2^ = 0.9989 (Fig. 5g). According to the above standard curve, the miRNA-155 expression levels in healthy individuals ranged from 0.1 to 0.8 pM, while in breast cancer patients, all values were significantly increased to 10–80 pM (Fig. 5h). The results indicated that the developed biosensor could distinguish between breast cancer patients and healthy individuals (**p < 0.01, Fig. 5i). In addition, its elimination of the need for extraction, purification, and amplification greatly simplifies the process of detection. Therefore, the potential of the developed biosensor as a clinical diagnostic tool for breast cancer was demonstrated.

## Discussions and Conclusions

In conclusion, by combining the CRISPR-Cas10 system with GFET, an amplification-free CRISPR-GFET RNA detection platform was developed. Under optimized conditions, the biosensor exhibited excellent sensitivity and specificity for both medium-length RNA and miRNA, with detection limits as low as 214 and 427 aM, respectively. In addition, the platform demonstrated excellent immunity to interference in throat swabs and serum samples, with recoveries ranging from 81.68% to 98.81% in throat swabs, confirming its accuracy. The ability to completely differentiate between healthy individuals and breast cancer patients without the need for extraction, purification, or amplification enhanced the acceptability of the method for clinical applications.

Compared to existing RNA detection methods based on the CRISPR-Cas13 system, the LdCsm-based detection system offers several significant advantages. Firstly, using DNA instead of RNA as the reporter enhances the stability of the detection system [31]. Secondly, the LdCsm-dCsm3 nuclease does not degrade the target RNA, enabling the self-amplification of the reaction system. Additionally, nonspecific RNA has minimal impact on LdCsm RNA detection, thereby improving the specificity of the detection platform [28]. Furthermore, the LdCsm effector complex is much more stable at room temperature compared to Cas13 enzymes, which increases the system’s lifespan and reduces reliance on cold-chain logistics [28]. However, previously developed CRISPR-Cas10 nucleic acid detection tools largely relied on cOA signal pathways activated by target RNA, rather than the DNase activity of the LdCsm system [74, 75, 76]. Although these cOA-based systems can achieve sensitive RNA detection, they are complex and predominantly rely on RNA reporter cleavage, leading to reduced stability.

In this work, we have integrated the CRISPR-Cas10 system with a GFET, fully utilizing the sustained self-amplification effect, target-inducible deoxyribonuclease activity of LdCsm-dCsm3 effector complex, the unique aptamer reporter, and the high sensitivity and rapid response capabilities of GFETs. This integration significantly addresses the sensitivity limitations of the LdCsm system, providing a detection platform that is simpler, more stable and has stronger anti-interference capabilities. Through the rational design of crRNA spacing sequences, the CRISPR-GFET platform is poised to become a universal RNA detection tool, applicable across a wide range of scenarios, including pathogen detection, genetic biomarker screening, and the diagnosis of disease-associated RNAs. Furthermore, the platform’s versatility can be extended to multiplex analysis. By designing CRISPR systems with multiple crRNAs and coupling them with GFET sensor arrays, the platform facilitates the simultaneous detection of multiple RNA targets, thereby enabling more comprehensive disease profiling. In addition, as one of the few molecular diagnostic technologies that does not require amplification, this platform significantly simplifies the process, reduces the risk of sample cross-contamination, and provides an efficient, reliable solution for POC testing. Looking ahead, further miniaturization of the GFET platform and integration with portable devices, such as smartphones, will enable remote, real-time testing. This innovative design not only allows patients to continuously monitor their health status but also facilitates real-time data transmission and online feedback, offering a more convenient and personalized approach to medicine and accurate diagnosis.

## Supplementary Information

Below is the link to the electronic supplementary material.

Supplementary file1 (DOCX 29088 kb)
