# Supplementary material for: Universal Amplification-Free RNA Detection by Integrating CRISPR-Cas10 with Aptameric Graphene Field-Effect Transistor
Source: Nanomicro Lett. 2025 Apr 30;17:242. doi: 10.1007/s40820-025-01730-3 (PMC12044126; doi:10.1007/s40820-025-01730-3)
Supplement: Supplementary file 1 — Supplementary file1 (DOCX 29088 kb) [file 40820_2025_1730_MOESM1_ESM.docx]

Supporting Information for

**Universal Amplification-Free RNA Detection by Integrating CRISPR-Cas10 with Aptameric Graphene Field-Effect Transistor**

Mingyuan Sun^1,#^, Zhenxiao Yu^2,#^, Shuai Wang^1^, Jiaoyan Qiu^1^, Yuzhen Huang^1^, Xiaoshuang Chen^1^, Yunhong Zhang^1^, Chao Wang^1^, Xue Zhang^1^, Yanbo Liang^1^, Hong Liu^3^, Qunxin She^2^, Yu Zhang^1,4,5,^* and Lin Han^1,4,5,^*

^1^ Institute of Marine Science and Technology, Shandong University, Qingdao, Shandong 266237, P. R. China

^2^ CRISPR and Archaea Biology Research Center, State Key Laboratory of Microbial Technology and Microbial Technology Institute, Shandong University, Qingdao, Shandong 266237, P. R. China

^3^ State Key Laboratory of Crystal Materials, Shandong University, Jinan, Shandong 250100, P. R. China

^4^ School of Integrated Circuits, Shandong University, Ji’nan, Shandong 250100, P. R. China

^5^Shandong Engineering Research Center of Biomarker and Artificial Intelligence Application, Jinan 250100, P. R. China

^#^Mingyuan Sun and Zhenxiao Yu contributed equally to this work.

*Corresponding authors. E-mail: [yuzhang@sdu.edu.cn](mailto:yuzhang@sdu.edu.cn) (Yu Zhang); [hanlin@sdu.edu.cn](mailto:hanlin@sdu.edu.cn) (Lin Han)

**S1 Experimental section**

**S1.1 Materials**

# Copper-based monolayer graphene sheets with a copper thickness of 25 μm were provided by ACS Materials. PI films (200 μm) for the fabrication of laser-induced graphene were supplied by Shenzhen Jinluye Electronic Materials Co., Ltd. PDMS monomer and curing agent were purchased from Momentive Performance Materials Inc. Poly(methyl methacrylate) (PMMA) was provided by Beijing Huidexin Technology Co. Glycerol, ferric chloride, acetone, ethanol, MgCl_2_, and KCl were obtained from Sinopharm Chemical Reagents (Shanghai, China). A 40% acrylic acid/double solution (37.5:1) was purchased from Coolaber. HAuCl_4_·3H_2_O was supplied by Aladdin Co. Ltd., while 6-mercapto-1-hexanol (MCH), tris(2-carboxyethyl)phosphine hydrochloride (TCEP), and RNaseZAP were purchased from Sigma-Aldrich. Tris-HCl (pH = 7) and phosphate-buffered saline (PBS) were provided by Corning Inc. Bovine serum albumin (BSA) was supplied by Thermo Fisher Scientific. For DNA reporter cleavage, the 10×cleavage buffer (500 nM Tris-HCl (PH=7),100 mM MgCl_2_, 500 mM KCl, and 1mg/ml BSA) was prepared and subsequently diluted in reaction solution to 1x cleavage buffer for use. Buffer A (20 mM Tris-HCl, 0.25 M NaCl, 20 mM imidazole, 10% glycerol, pH8.5), Buffer B (20 mM Tris-HCl, 0.25 M NaCl, 200 mM imidazole, 10% glycerol, and pH 8.5) and chromatography buffer (20 mM Tris-HCl, 0.25 M NaCl, 5% glycerol, and pH 8.5) were used for the purification of LdCsm effector complex. Sangon Biotech Co., Ltd. (Shanghai, China) supplied the miRNA First Strand cDNA Synthesis Kit (Stem-loop Method) and the MicroRNAs qPCR Kit (SYBR Green Method) for RT-qPCR experiments. Additionally, all DNA, RNA, and miRNA sequences used in this study were synthesized and purified by Sangon Biotech and are listed in Table S4. All chemical reagents were of analytical grade, and RNase-free water was used throughout the study.

**S1.2 Blood sample collection**

# To verify the reliability and performance of our CRISPR-GFET biosensor in detecting real samples, we collected serum samples from clinical breast cancer patients and healthy individuals for miRNA-155 detection. Specifically, blood was drawn from volunteers using a non-anticoagulant tube and left to stand at room temperature for 5-6 hours. Afterward, the tube was observed to ensure the separation of the serum (upper yellowish liquid) from the red precipitate (lower layer). Using a pipette with a disposable sterile tip, the upper yellowish liquid was carefully aspirated and transferred into a 1.5 mL microcentrifuge tube. The tube was then centrifuged at 4°C (5000 rpm, 10 minutes), and the supernatant was carefully transferred into a new tube. The serum was then stored at -80°C for further use.

**S1.3 Cleavage of fluorescent DNA reporter by LdCsm and LdCsm-dCsm3**

# To investigate the effectiveness and cleavage preference of LdCsm, LdCsm-dCsm3 (LdCsm-dCsm3-RNA, LdCsm-dCsm3-155, LdCsm-dCsm3-155-A-5U), as well as the effect of target sequence length on them, a fluorescence analysis was performed. A total of 20 μL of test solution was prepared, which included different target RNAs (50 nM), FAM-BHQ1-labeled ssDNA reporter (1 μM), 1× cleavage buffer, and effector complex (200 nM). After incubation at 37 °C for 30 min, the fluorescence spectrum of this test solution was measured with a fluorescence spectrophotometer. Notably, the relative fluorescence intensity (RFI) in the text is the fluorescence intensity value obtained for the sample mixture containing the target RNA/miRNA or the RNA/miRNA to be detected minus the fluorescence intensity of the reference sample (lacking the target RNA/miRNA).

# S1.4 Gel electrophoresis for evaluating in vitro hybridization feasibility

# To assess the feasibility of in vitro hybridization, non-denaturing polyacrylamide gel electrophoresis (PAGE) was performed. Specifically, LdCsm-dCsm3 ribonucleoprotein (RNP) complexes at concentrations of 50 nM and 200 nM were incubated with 50 nM of 5ʹ-FAM-labeled RNA samples, including both target and non-target RNAs, in lysis buffer at 37°C for 20 minutes to facilitate RNA binding. Following incubation, the samples were immediately placed on ice and an equal volume of 30% glycerol was added to enhance sample loading and prevent diffusion during electrophoresis. The mixtures were then subjected to electrophoresis on a 5% non-denaturing polyacrylamide gel using 1× TBE buffer at 4°C. Fluorescence imaging was subsequently performed to visualize and analyze the mobility shifts of the RNA-protein complexes.

**S1.5 RT-PCR for RNA detection**

# In this study, a two-step reverse transcription quantitative PCR (RT-qPCR) assay was employed to quantify RNA samples. First, complementary DNA (cDNA) was synthesized using a miRNA First Strand cDNA Synthesis Kit (Stem-loop Method). The reaction mixture consisted of 10 μL of 2× miRNA L-RT solution mix, 1.5 μL of miRNA L-RT enzyme mix, 2 μL of RNA template, 1 μL of stem-loop primer, and 5.5 μL of DNase/RNase-free water. The reaction conditions were as follows: incubation at 16°C for 30 min to extend the primers, followed by incubation at 37°C for 30 min to complete reverse transcription, and a final heating step at 85°C for 5 min to inactivate the enzyme. The resulting cDNA was used directly for subsequent qPCR amplification. Next, qPCR amplification was performed using a MicroRNAs qPCR Kit (SYBR Green Method). The reaction mixture comprised 10 μL of 2× miRNA qPCR Master Mix, 0.5 μL of forward primer, 0.5 μL of reverse primer, 2 μL of cDNA template, and 7 μL of DNase/RNase-free water. The qPCR program included an initial denaturation at 95°C for 30 s, followed by enzyme activation at 40°C for 5 min. This was followed by 40 cycles of 95°C denaturation for 5 s and 60°C annealing/extension for 30 s, during which fluorescence signals were acquired.

**S1.6 Characterizations and measurements**

# The fluorescence spectrum was taken by Spark microplate reader (TECAN, Switzerland), with a λex/λem at 490/520 nm. Gel electrophoresis experiments using the BIO-RAD PowerPac™ HC power supply and BIO-RAD Mini PROTEANR Tetra Cells. An environmental scanning electron microscope (Thermo Scientific, Quattro S) with a Bruker Bruker QUANTAX EDS X-ray energy spectrometer was used to measure scanning electron microscopy (SEM) and energy dispersive X-ray spectroscopy (EDS). Electrochemical impedance spectroscopy (EIS) was tested in 0.1 M KCl containing 5 mM Fe(CN)_6_^3-/4-^ using a CHI 660E electrochemical workstation (Shanghai Chenhua Instruments, China). Fluorescence images were scanned by GenePix 4400A scanner using a 488 nm laser, and analyzed using GenePix pro software. X-ray photoelectron spectroscopy (XPS) was conducted through a Thermo Scientific K-Alpha with an Al K alpha ray excitation source (*hv*=1486.6 eV). Reverse transcription and fluorescence quantitative PCR were performed on a Bio-Rad system (Bio-RAD CFX384 Touch, USA). The transfer characteristics (*V*_g_-*I*_ds_) of the CRISPR-GFETs were measured by a probe station with a Keithley 2636 source meter. For the transfer characteristics, *V*_g_ was scanned from −300 to 300 mV at a *V*_ds_ of 50 mV. The biosensor's specificity, sensitivity, anti-interference capability and potential for practical applications were assessed by monitoring the *V*_dirac_ displacement in the *I*_ds_-*V*_g_ curve of the biosensor before and after its reaction with different concentrations and species of RNA/miRNAs as well as real samples.

**S2 Supplementary Figures**


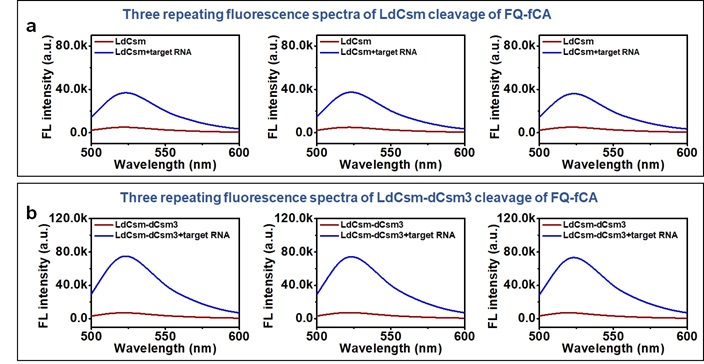


Fig. S1 Validation of the effectiveness of LdCsm / LdCsm-dCsm3 mediated cleavage. (**a**)Three repeating fluorescence spectra of LdCsm cleavage of FQ-fCA in the absence/presence of target RNA. (**b**)Three repeating fluorescence spectra of LdCsm-dCsm3 cleavage of FQ-fCA in the absence/presence of target RNA


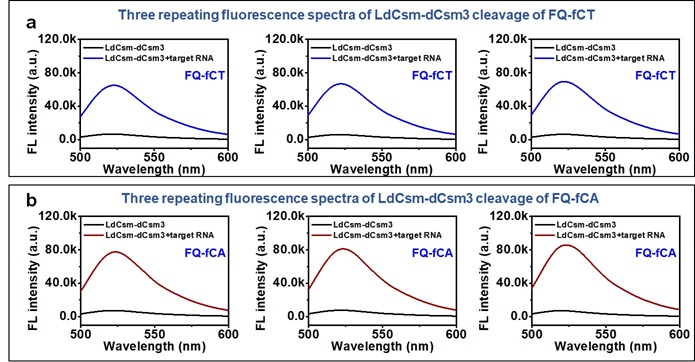


Fig. S2 The nucleotide cleavage preference of the LdCsm-dCsm3 system. (**a**) Three repeating fluorescence spectra obtained by cleavage of FQ-fCT by activated LdCsm-dCsm3 RNP. (**b**) Three repeating fluorescence spectra obtained by cleavage of FQ-fCA by activated LdCsm-dCsm3 RNP.

Target RNAs with 5′-repeat tags and protospacer sequences of different lengths are matched to LdCsm-dCsm3-crRNA as shown in **Fig. S3**. Among them, protospacer target RNAs (PTRs) are target RNAs without any3′-protospacer flanking sequence (3′-antitag). Cognate target RNA (CTRs) are RNAs carrying sequences complementary to the spacer region of LdCsm-dCsm3-crRNA and with a 3′-antitag that does not match the 5′-repeat tag of the crRNA.


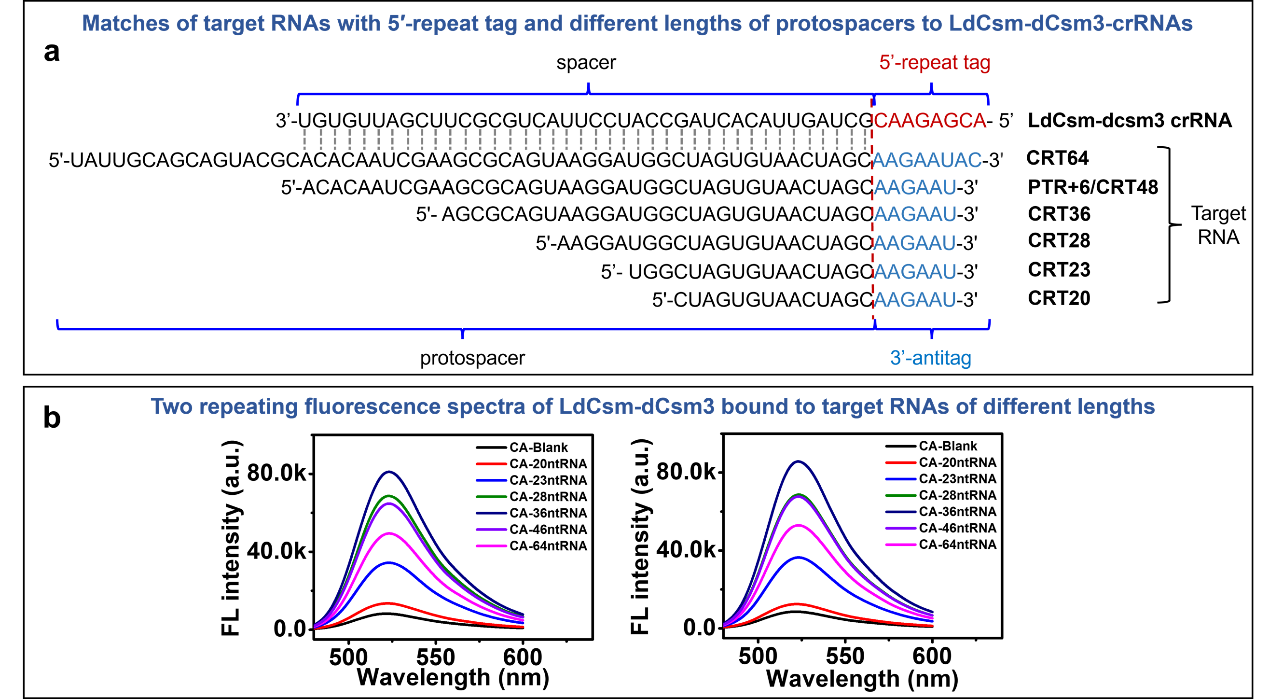


Fig. S3 The effect of target RNA length on the extent of LdCsm-dCsm3RPN activation. (**a**) Matches of target RNAs with 5′-repeat tag and different lengths of protospacers to LdCsm-dCsm3-crRNAs. (**b**) Two repeating fluorescence spectra obtained by cleavage of FQ-FCA after binding of LdCsm-dCsm3 to target RNAs of different lengths (20nt, 23nt, 28nt, 36nt, 46nt, 64nt)

# The conductivity and consistency of LIG electrodes are critical to the performance of FETs. To further assess the conductivity and uniformity of the transferred LIG, we conducted a series of tests, including Raman spectroscopy, optical imaging, and *I*-*V* curve measurements. As shown in Fig. S4a, the LIG transfer electrodes exhibited good conductivity, and optical imaging confirmed their uniformity. Raman spectroscopy was performed to gain deeper insight into the homogeneity of the LIG transfer electrode. As shown in Fig. S4b, the Raman spectrum reveals the characteristic peaks of LIG, with an *I*_D_/*I*_G_ ratio of 0.60, indicating a high degree of defect formation [S1]. The inset of Fig. S4b presents a spatial Raman map of LIG in a 250 × 250 μm² area, with a relative standard deviation of less than 1.1%, suggesting that the LIG electrode maintained its integrity and consistency after the transfer process. Therefore, the LIG-transferred electrodes demonstrate excellent uniformity and conductivity.

#
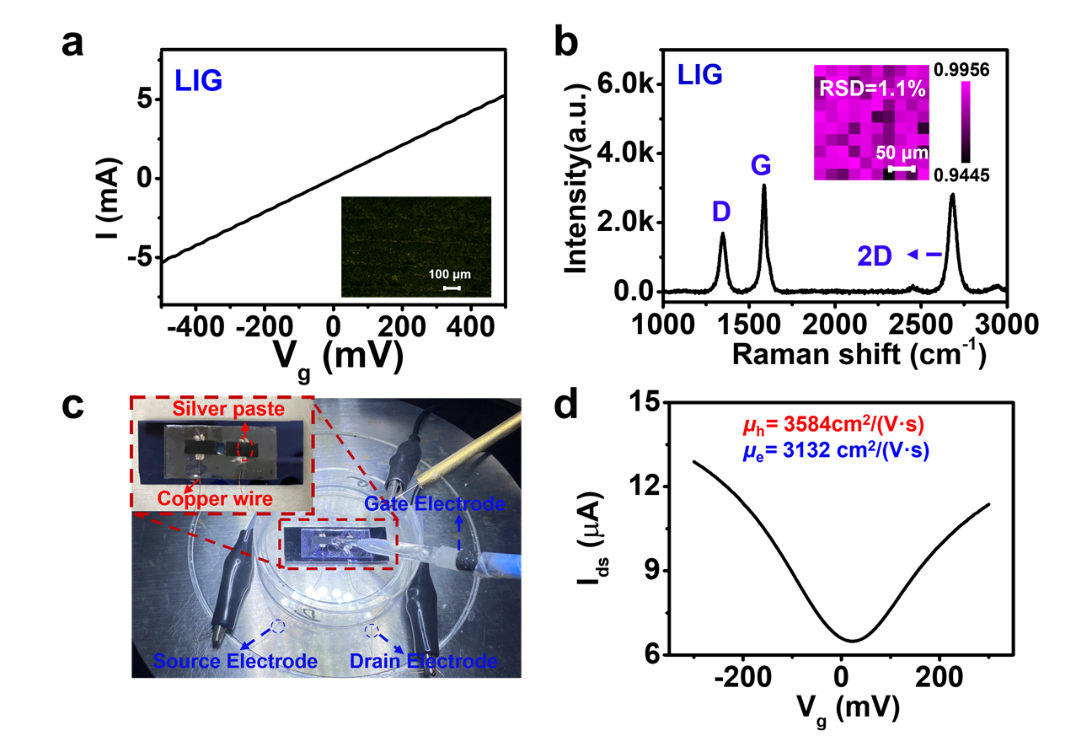


**Fig. S4** Preparation and electrical performance evaluation of GFETs. (**a**) *I*-*V* curves of LIG, inset is an optical image of LIG. (**b**) Raman spectrum and Raman mapping image (inset) of the LIG. (**c**) Physical and test picture of the device. (**d**) The transfer curves of GFET

# We first deposited gold nanoparticles on slides and subsequently immobilized FAM-labeled thiolated linear DNA reporters (re-FAM-lDNA). As shown in the first and second left panels of Fig. S5b, the fluorescence intensity in the fluorescence images and thermograms increased significantly after the immobilization of re-FAM-lDNA, which demonstrated that the DNA reporter could be immobilized by Au-S bonding. The complete LdCsm-dCsm3 mixture (including LdCsm-dCsm3 effector complex, lysis buffer, RNase inhibitor and target RNA) and the LdCsm-dCsm3 mixture without target RNA were introduced into the re-FAM-lDNA-modified chip, respectively. As shown in Fig. S5b, the control group (no target; thrid left panel) showed a distinct green fluorescence after incubation similar to that before incubation (second plate from left), while the green fluorescence of the experimental group (with target; fourth left panel) was significantly diminished. The results of this experiment indicate the feasibility of a trans-cleavage effect at the solid-liquid interface. Subsequently, we characterized graphene, graphene surface-modified gold nanoparticles (AuNPs), and samples after re-hpDNA immobilization using XPS. As shown in Fig. S5c, the successful modification of AuNPs on the graphene surface was confirmed by the appearance of distinct Au 4f characteristic peaks in the XPS spectrum, with Au 4f_7/2_ and Au 4f_5/2_ located at ~83.7 eV and ~87.4 eV, respectively. Further analysis revealed that the AuNP sample exhibited an almost negligible S 2p signal. However, after re-hpDNA immobilization, a prominent S 2p peak emerged at ~162.2 eV, indicating the successful attachment of sulfhydryl-modified DNA onto the AuNP surface via Au–S bonding. Additionally, compared to the AuNP sample, the re-hpDNA sample showed P 2p and N 1s signals at 134.2 eV and 400.0 eV, respectively (Fig. S5d–f), further confirming the successful immobilization of DNA molecules. In summary, the XPS results conclusively demonstrated the effective modification of AuNPs on the graphene surface and the subsequent immobilization of re-hpDNA *via* Au–S interactions.

#

#
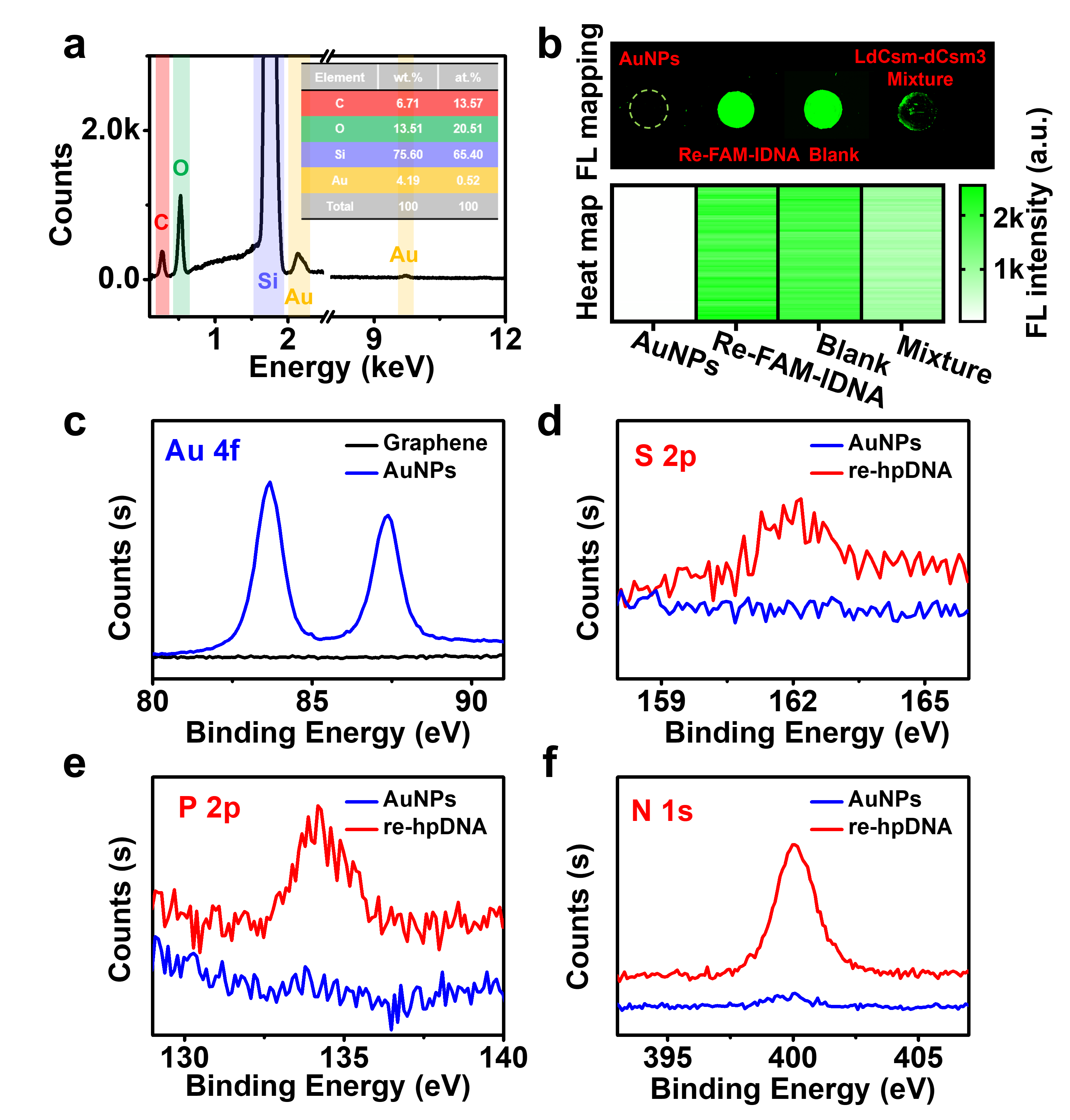
Fig. S5 Characterization of CRISPR-GFET biosensor. (a) EDS after deposition of AuNPs on the graphene surface. (d) Fluorescence scanning map and heat map of AuNP deposition (first left panel), re-FAM-lDNA modification (second left panel) and incubation of LdCsm-dCsm3 mixture without (thrid left panel) and with target RNA (fourth left panel). XPS spectra of (c) Au, (d) S, (e) P, and (f) N elements

#
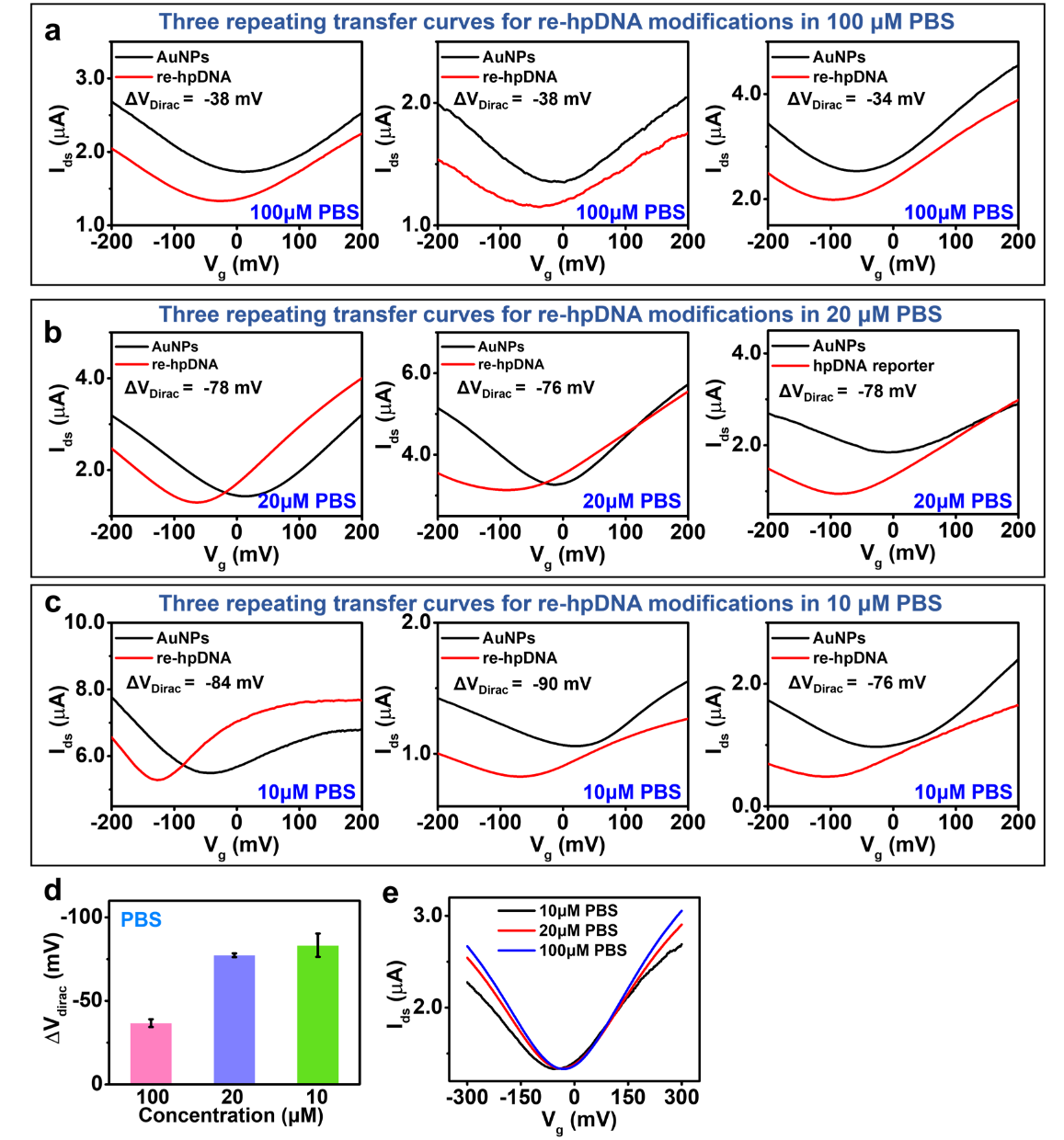


# Fig. S6 Validation of the optimal test concentration for re-hpDNA modified GFET. (a) Three repeating transfer curves of GFETs before and after re-hpDNA modification in 100 μM of PBS. (b) Three repeating transfer curves of GFETs before and after re-hpDNA modification in 20 μM of PBS. (c) Three repeating transfer curves of GFETs before and after re-hpDNA modification in 10 μM of PBS. (d) Optimization of the CRISPR-GFET biosensor for testing solution concentrations, evaluated by the transfer curve in Fig. S6a-c. (e) The transfer curves of re-hpDNA modified GFET operation in different concentrations (100, 20 and 10μM) of test solution (PBS)


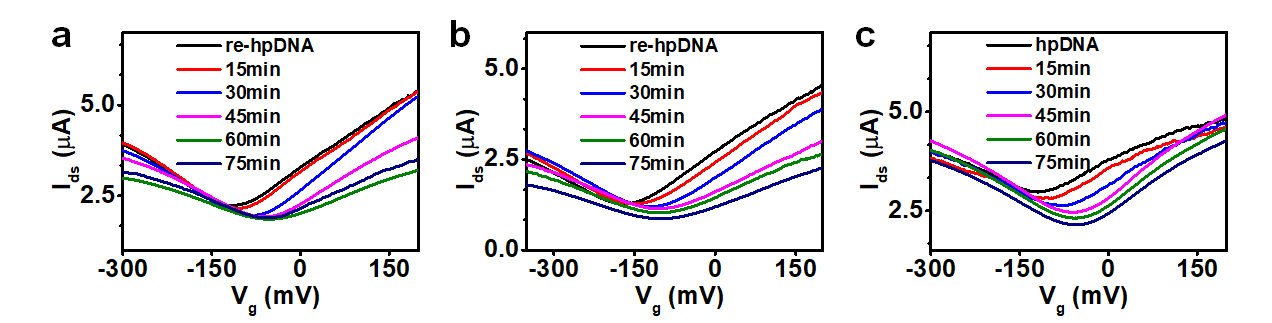


# Fig. S7 (a-c) Three repeating transfer curves of CRISPR-GFET biosensors incubated with LdCsm-dCsm3 mixture for different times (15, 30, 45, 60, 75 min)


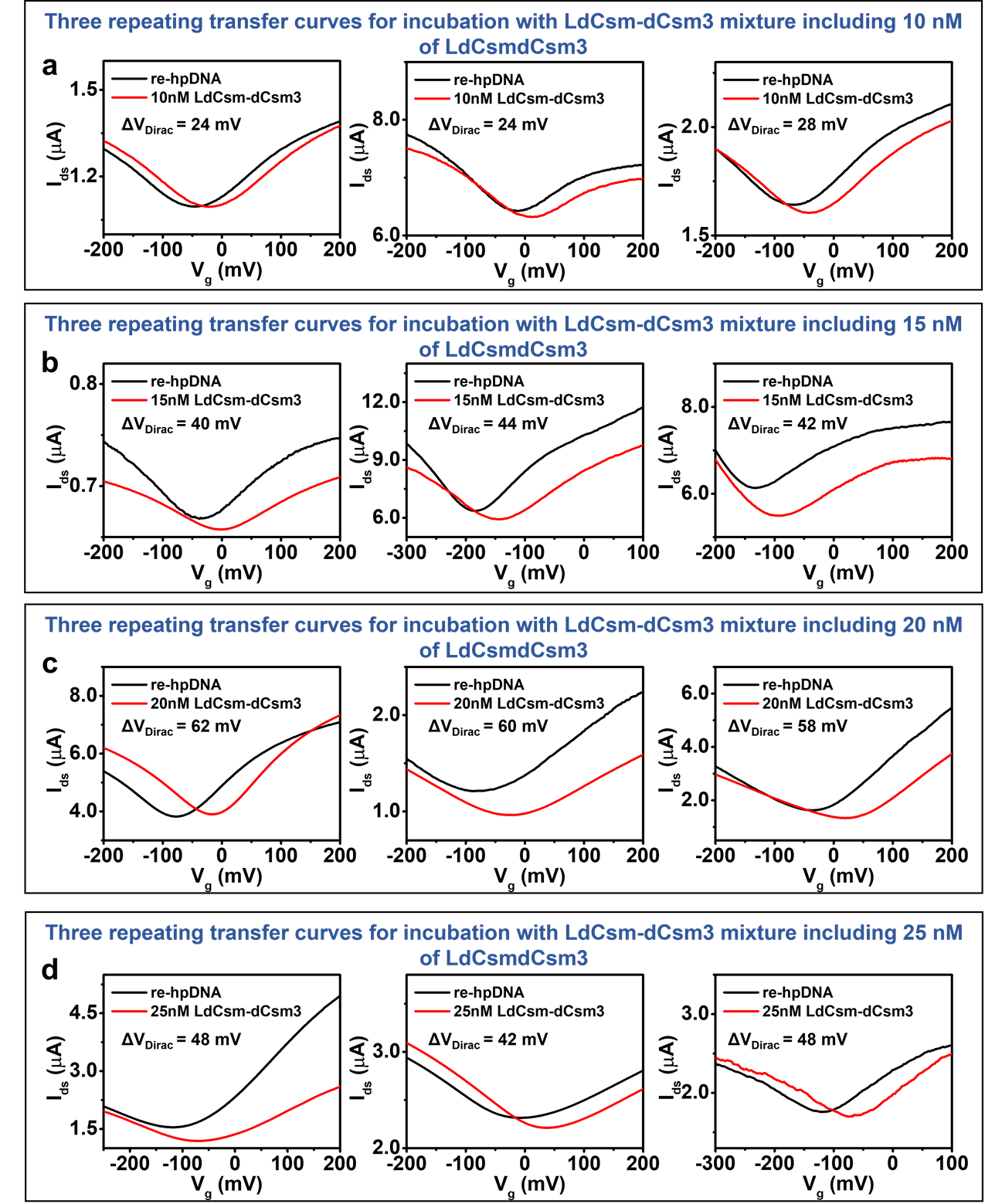


# Fig. S8 The transfer curves of CRISPR-GFET biosensors for incubation with LdCsm-dCsm3 mixture including different concentrations of LdCsm-dCsm3. (a) Three repeating transfer curves of CRISPR-GFET biosensors before and after incubation with LdCsm-dCsm3 mixture including 10 nM LdCsm-dCsm3. (b) Three repeating transfer curves of CRISPR-GFET biosensors before and after incubation with LdCsm-dCsm3 mixture including 15 nM LdCsm-dCsm3. (c) Three repeating transfer curves of CRISPR-GFET biosensors before and after incubation with LdCsm-dCsm3 mixture including 20 nM LdCsm-dCsm3. (d) Three repeating transfer curves of CRISPR-GFET biosensors before and after incubation with LdCsm-dCsm3 mixture including 25 nM LdCsm-dCsm3


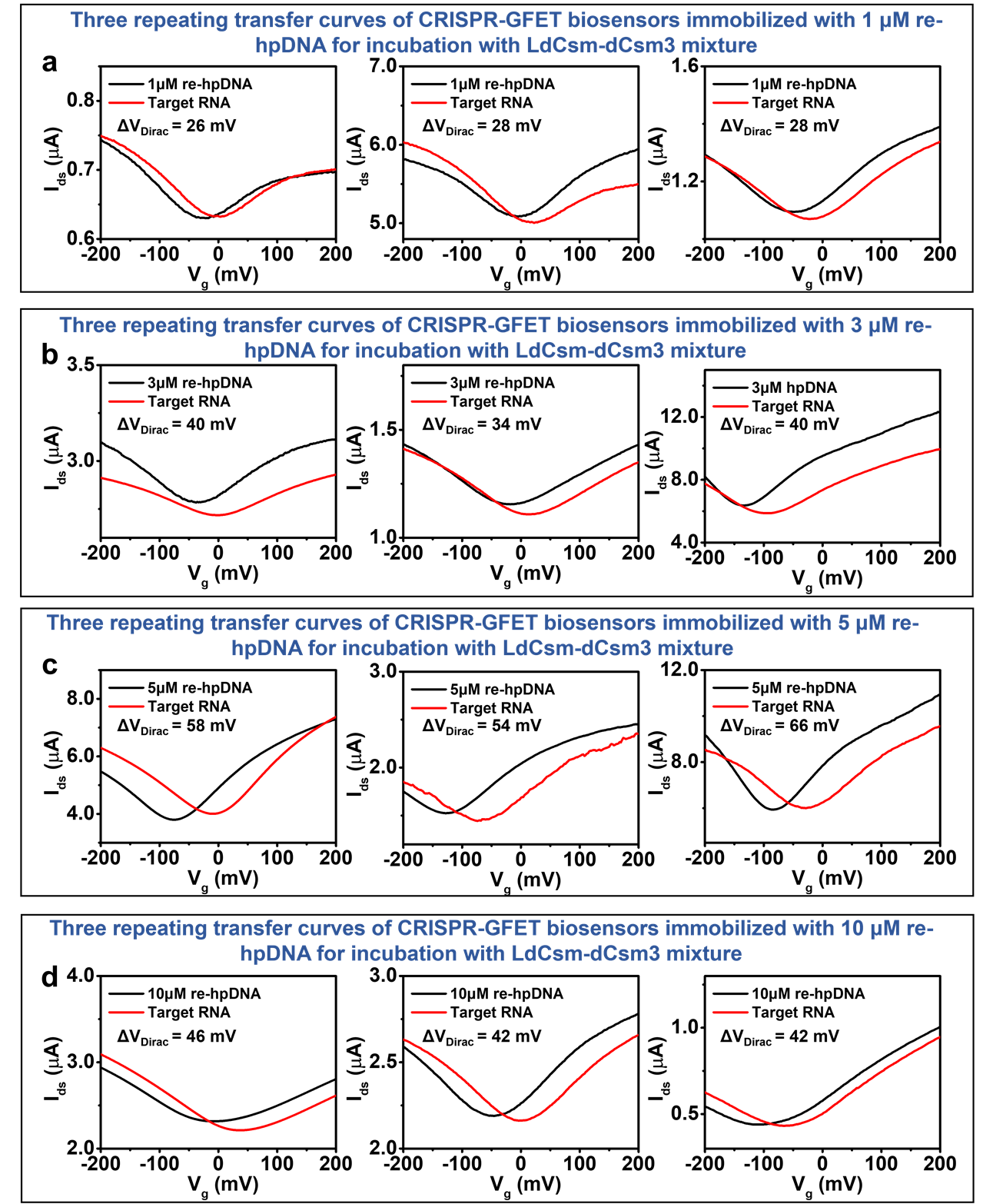


# Fig. S9 The transfer curves of CRISPR-GFET biosensors immobilized with different concentrations of re-hpDNA for incubation with LdCsm-dCsm3 mixture. (a) Three repeating transfer curves of CRISPR-GFET biosensors immobilized with 1 μM re-hpDNA before and after incubation with LdCsm-dCsm3 mixture. (b) Three repeating transfer curves of CRISPR-GFET biosensors immobilized with 3 μM re-hpDNA before and after incubation with LdCsm-dCsm3 mixture. (c) Three repeating transfer curves of CRISPR-GFET biosensors immobilized with 5 μM re-hpDNA before and after incubation with LdCsm-dCsm3 mixture. (d) Three repeating transfer curves of CRISPR-GFET biosensors immobilized with 10 μM re-hpDNA before and after incubation with LdCsm-dCsm3 mixture


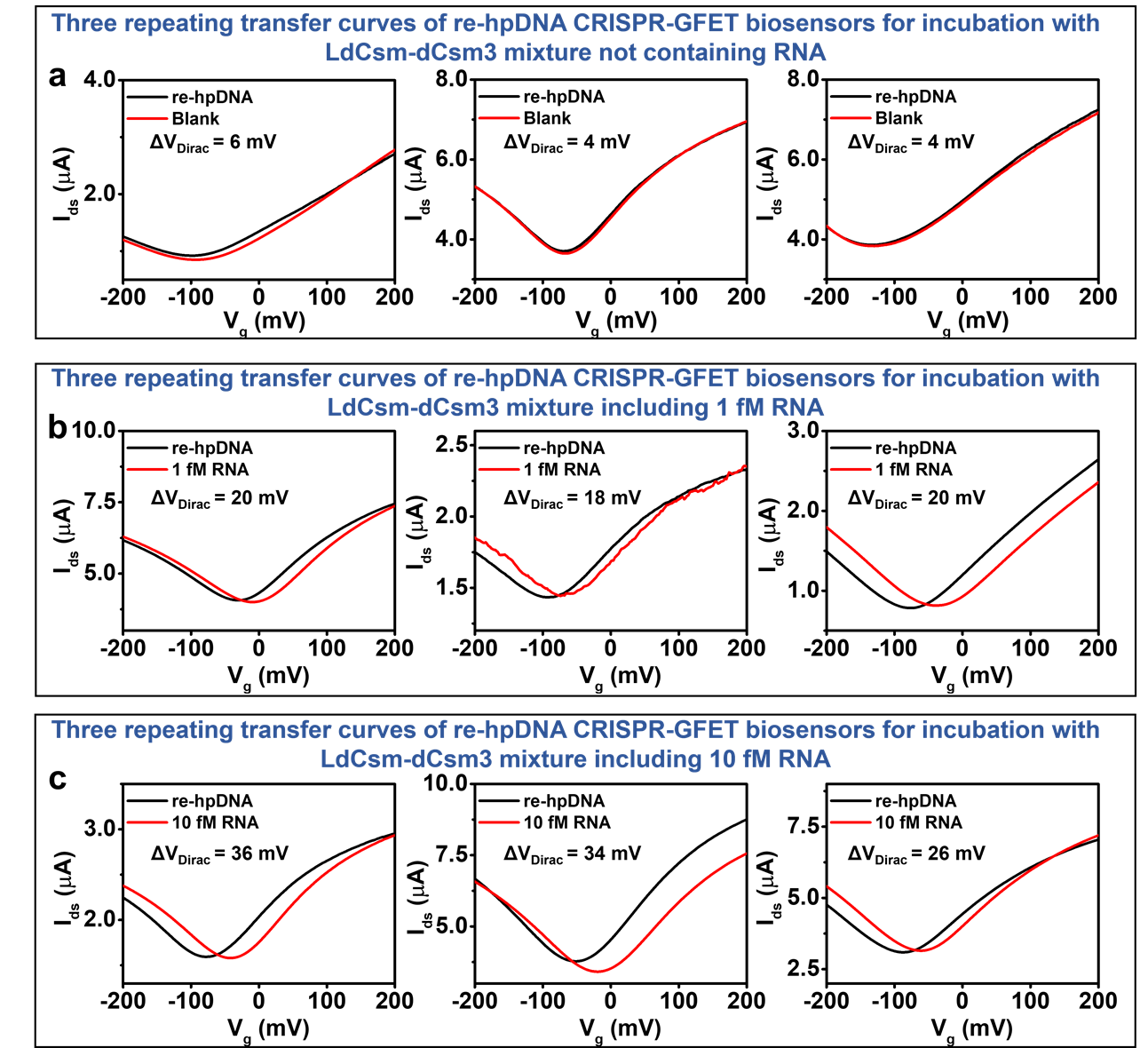


# Fig. S10 The transfer curves of re-hpDNA CRISPR-GFET biosensors for incubation with LdCsm-dCsm3 mixture including different concentrations of RNA. (a) Three repeating transfer curves of re-hpDNA CRISPR-GFET biosensors before and after incubation with LdCsm-dCsm3 mixture not containing RNA. (b) Three repeating transfer curves of re-hpDNA CRISPR-GFET biosensors before and after incubation with LdCsm-dCsm3 mixture including 1 fM RNA. (c) Three repeating transfer curves of re-hpDNA CRISPR-GFET biosensors before and after incubation with LdCsm-dCsm3 mixture including 10 fM RNA

#
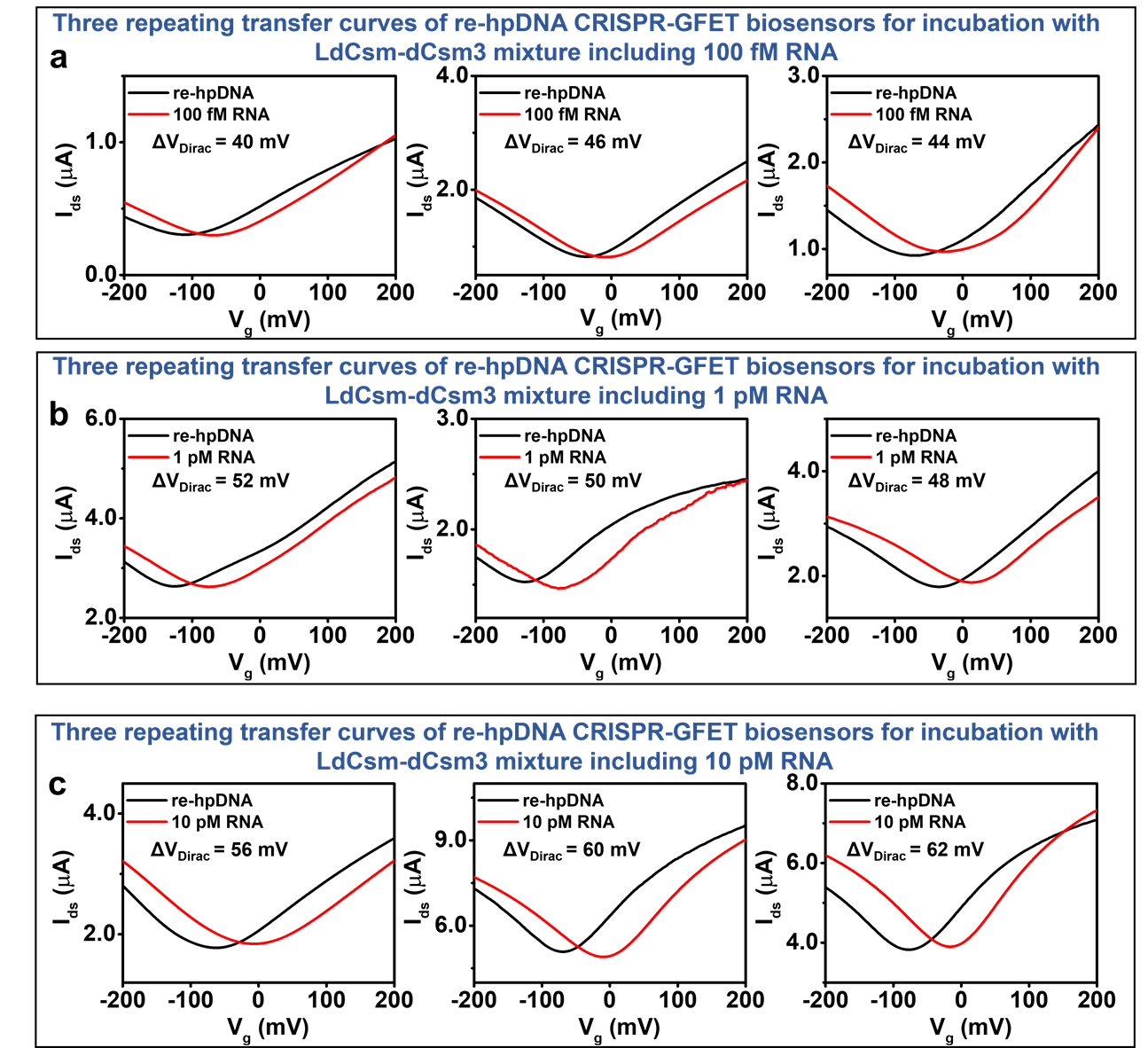


# Fig. S11 The transfer curves of re-hpDNA CRISPR-GFET biosensors for incubation with LdCsm-dCsm3 mixture including different concentrations of RNA. (a) Three repeating transfer curves of re-hpDNA CRISPR-GFET biosensors before and after incubation with LdCsm-dCsm3 mixture including 100 fM RNA. (b) Three repeating transfer curves of re-hpDNA CRISPR-GFET biosensors before and after incubation with LdCsm-dCsm3 mixture including 1 pM RNA. (c) Three repeating transfer curves of re-hpDNA CRISPR-GFET biosensors before and after incubation with LdCsm-dCsm3 mixture including 10 pM RNA


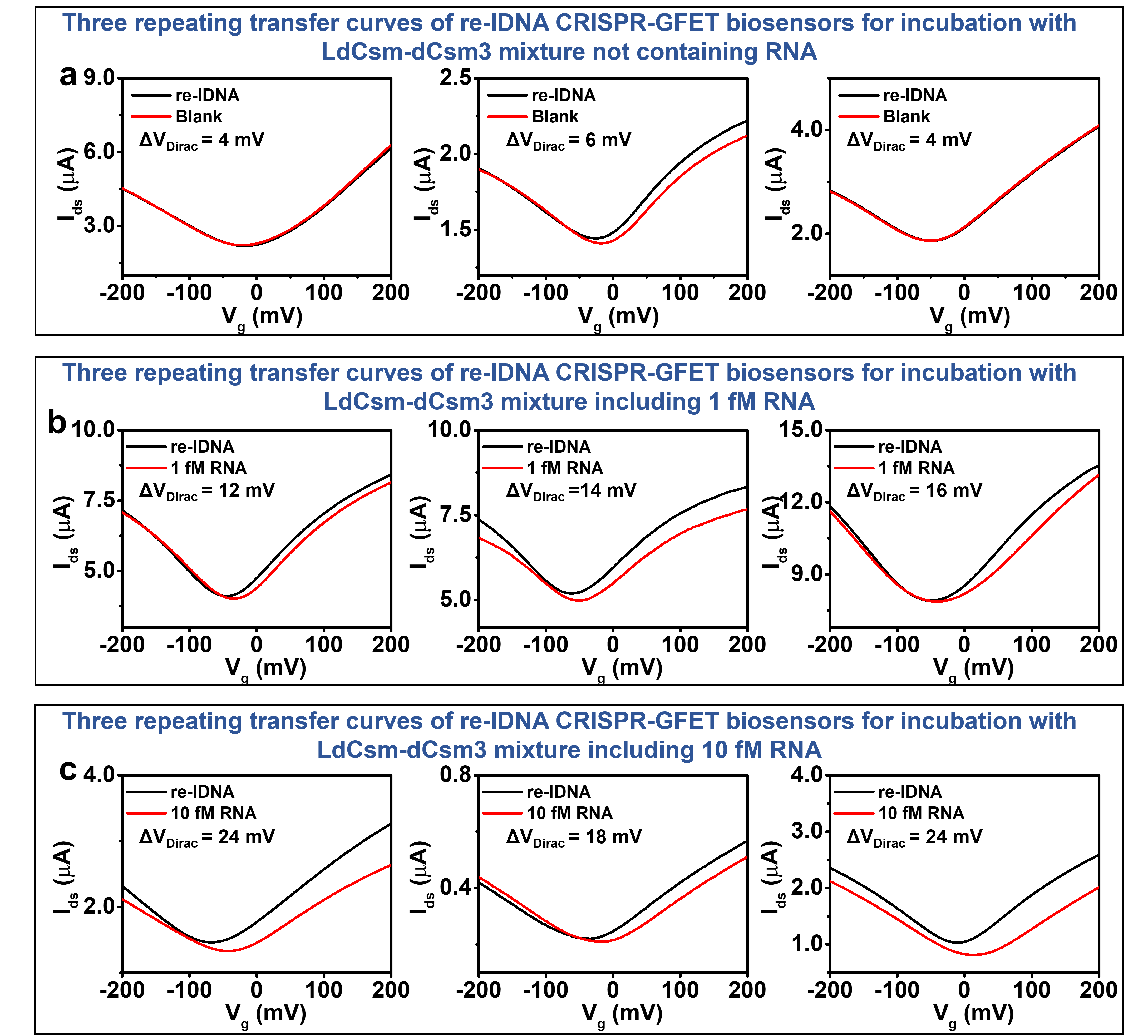


# Fig. S12 The transfer curves of re-lDNA CRISPR-GFET biosensors for incubation with LdCsm-dCsm3 mixture including different concentrations of RNA. (a) Three repeating transfer curves of re-lDNA CRISPR-GFET biosensors before and after incubation with LdCsm-dCsm3 mixture not containing RNA. (b) Three repeating transfer curves of re-lDNA CRISPR-GFET biosensors before and after incubation with LdCsm-dCsm3 mixture including 1 fM RNA. (c) Three repeating transfer curves of re-lDNA CRISPR-GFET biosensors before and after incubation with LdCsm-dCsm3 mixture including 10 fM RNA

#
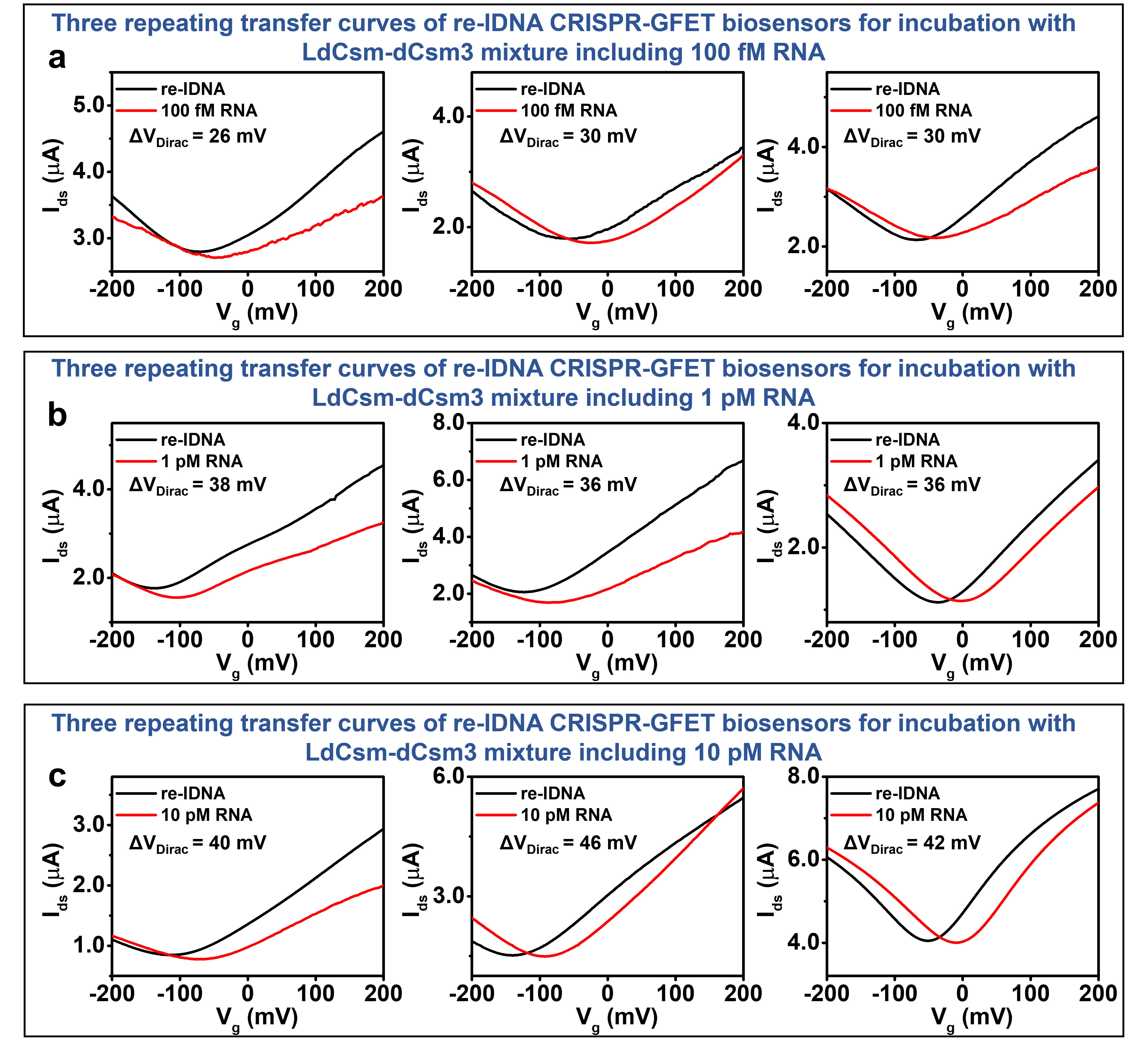


# Fig. S13 The transfer curves of re-lDNA CRISPR-GFET biosensors for incubation with LdCsm-dCsm3 mixture including different concentrations of RNA. (a) Three repeating transfer curves of re-lDNA CRISPR-GFET biosensors before and after incubation with LdCsm-dCsm3 mixture including 100 fM RNA. (b) Three repeating transfer curves of re-lDNA CRISPR-GFET biosensors before and after incubation with LdCsm-dCsm3 mixture including 1 pM RNA. (c) Three repeating transfer curves of re-lDNA CRISPR-GFET biosensors before and after incubation with LdCsm-dCsm3 mixture including 10 pM RNA


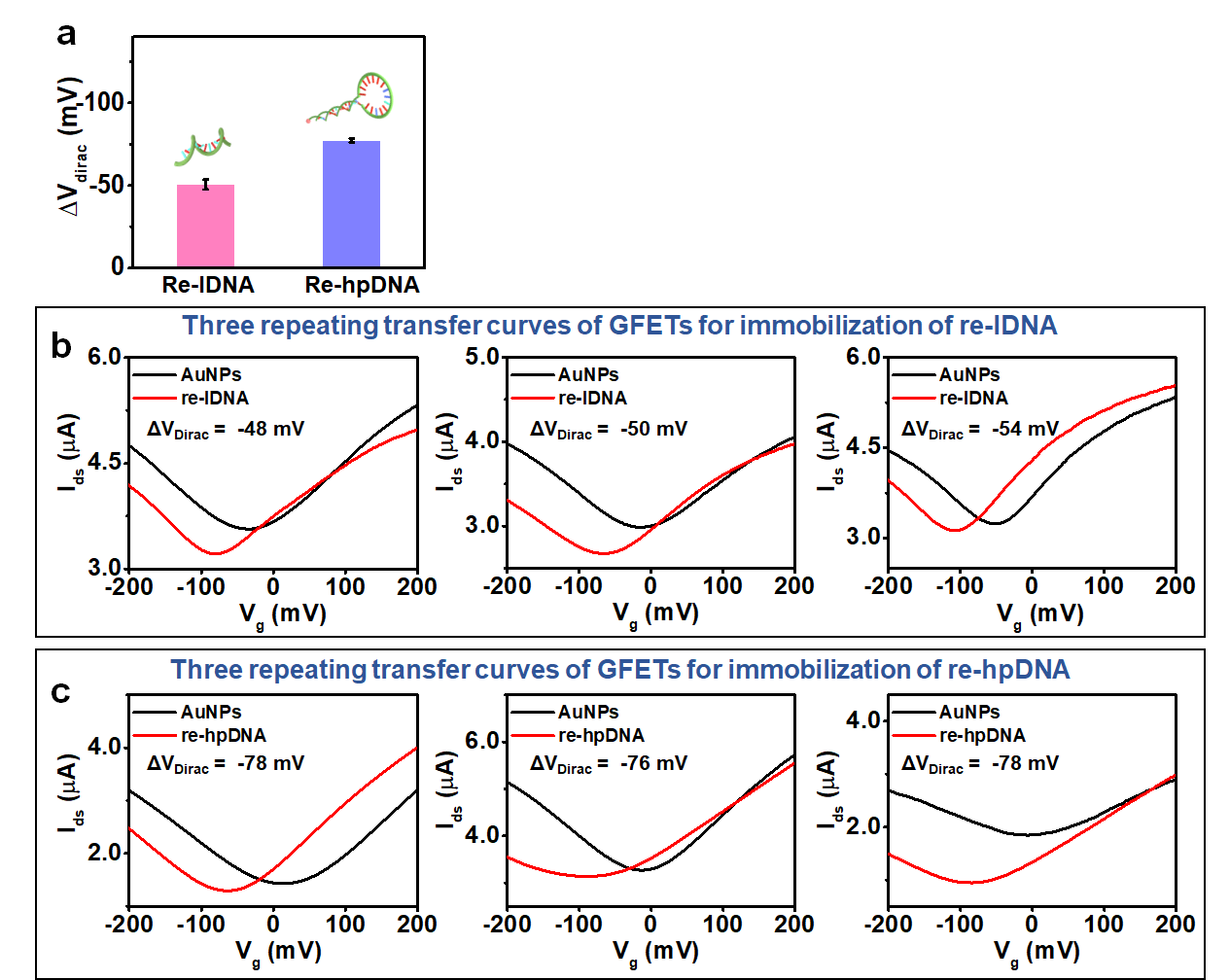


Fig. S14 Comparison of electrical properties of GFETs loading with linear DNA and hairpin DNA reporter. (**a**) Comparison of Δ*V_dirac_* after GFET loading with linear and hairpin DNA reporters. (**b**) Three repeating transfer curves of GFETs before and after immobilization of re-lDNA. (**c**) Three repeating transfer curves of GFETs before and after immobilization of re-hpDNA


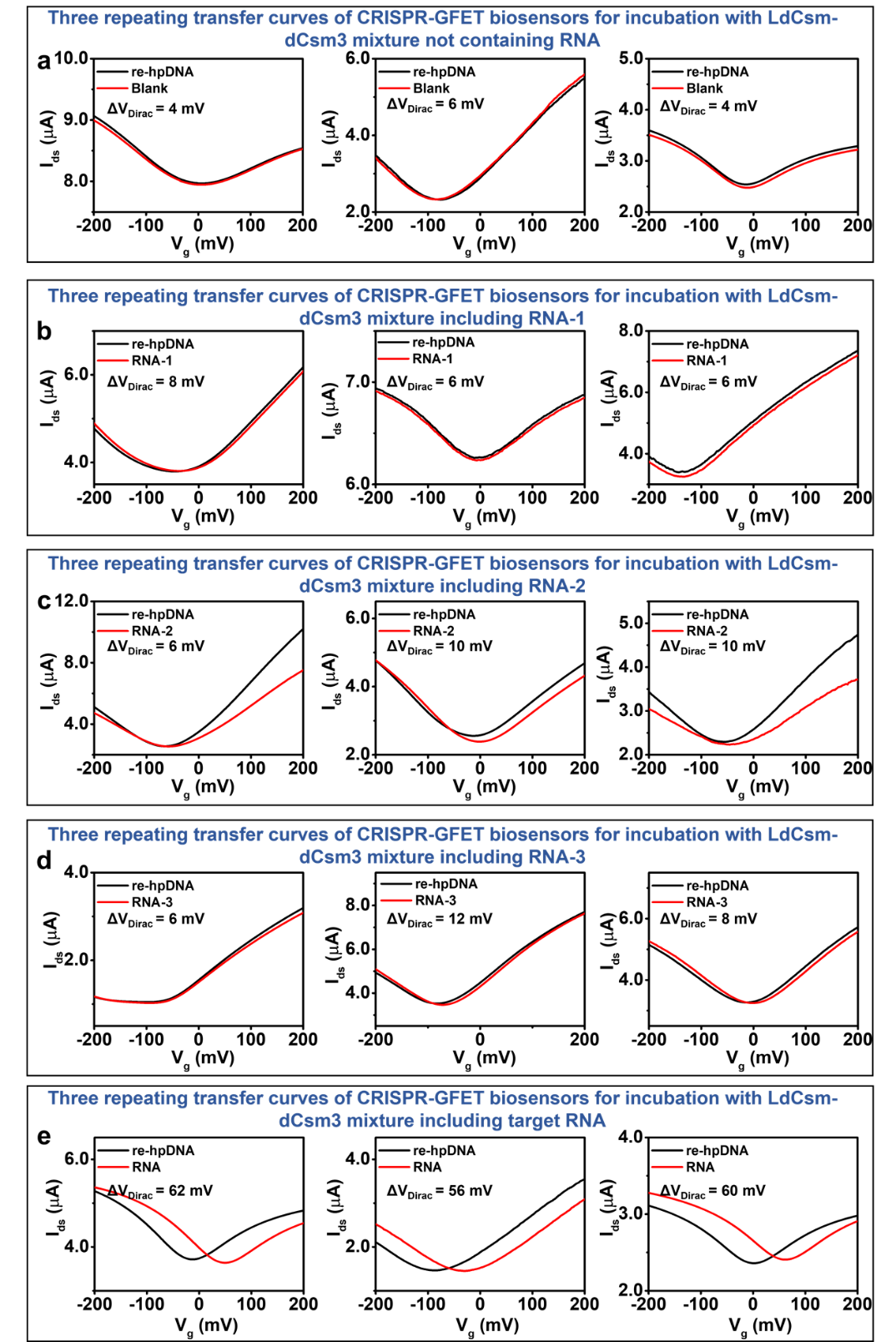


# Fig. S15 The transfer curves of CRISPR-GFET biosensors for incubation with LdCsm-dCsm3 mixture with RNA-free (blank), non-target, or target RNA. (a) Three repeating transfer curves of CRISPR-GFET biosensors before and after incubation with LdCsm-dCsm3 mixture not containing RNA. (b) Three repeating transfer curves of CRISPR-GFET biosensors before and after incubation with LdCsm-dCsm3 mixture including RNA-1. (c) Three repeating transfer curves of CRISPR-GFET biosensors before and after incubation with LdCsm-dCsm3 mixture including RNA-2. (d) Three repeating transfer curves of CRISPR-GFET biosensors before and after incubation with LdCsm-dCsm3 mixture including RNA-3. (e) Three repeating transfer curves of CRISPR-GFET biosensors before and after incubation with LdCsm-dCsm3 mixture including target RNA

**
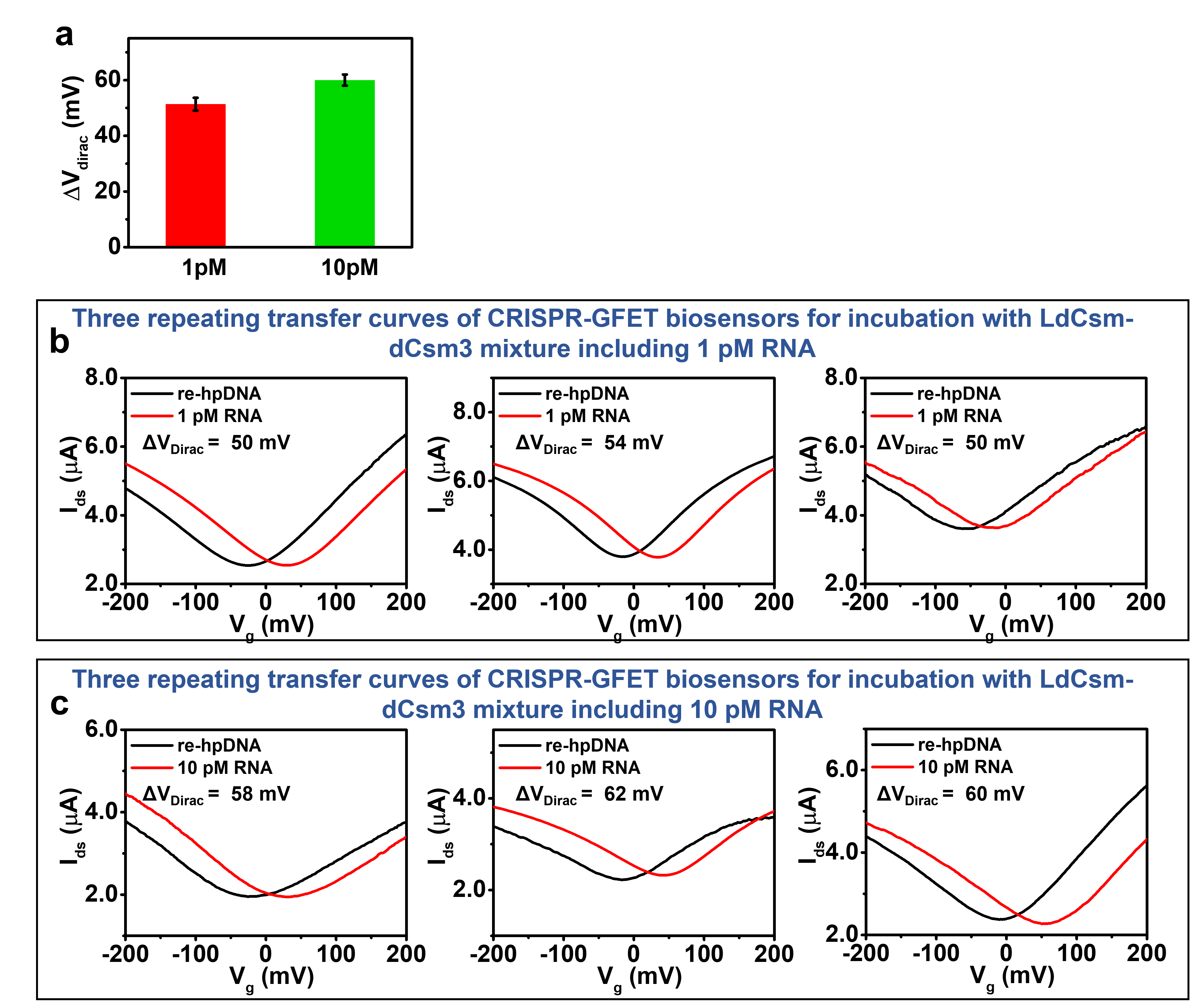
**Fig. S16 Reproducibility of CRISPR-GFET biosensors (**a**) Response of multiple CRISPR-GFET biosensors to 1pM and 10pM RNA, respectively. (**b**) Three repeating transfer curves of CRISPR-GFET biosensors before and after incubation with LdCsm-dCsm3 mixture including 1 pM RNA. (**c**) Three repeating transfer curves of CRISPR-GFET biosensors before and after incubation with LdCsm-dCsm3 mixture including 10 pM RNA


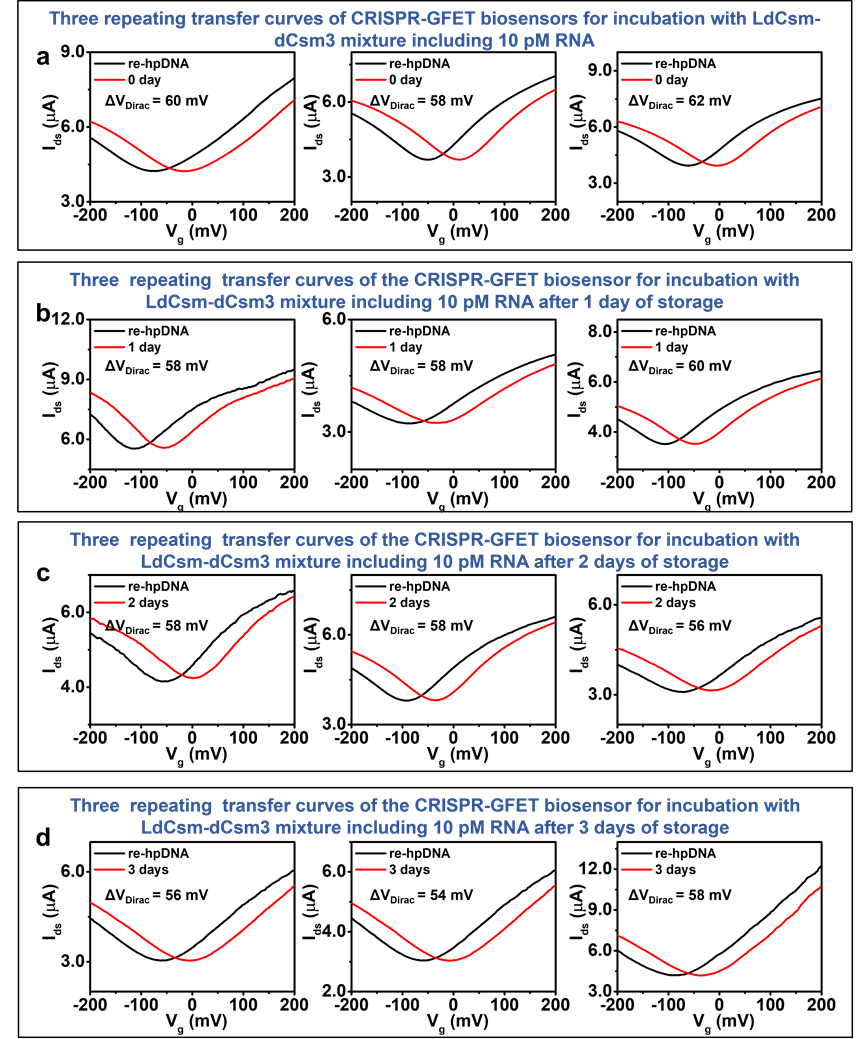


Fig. S17 Transfer curves of CRISPR-GFET after incubation with 10 pM RNA at different storage times. (**a**) Three repeating transfer curves of CRISPR-GFET biosensors before and after incubation with LdCsm-dCsm3 mixture including 10 pM RNA. (**b**) Three repeating transfer curves of CRISPR-GFET biosensors before and after incubation with LdCsm-dCsm3 mixture including 10 pM RNA after 1 day of storage. (**c**) Three repeating transfer curves of CRISPR-GFET biosensors before and after incubation with LdCsm-dCsm3 mixture including 10 pM RNA after 2 days of storage. (**d**) Three repeating transfer curves of CRISPR-GFET biosensors before and after incubation with LdCsm-dCsm3 mixture including 10 pM RNA after 3 days of storage

#
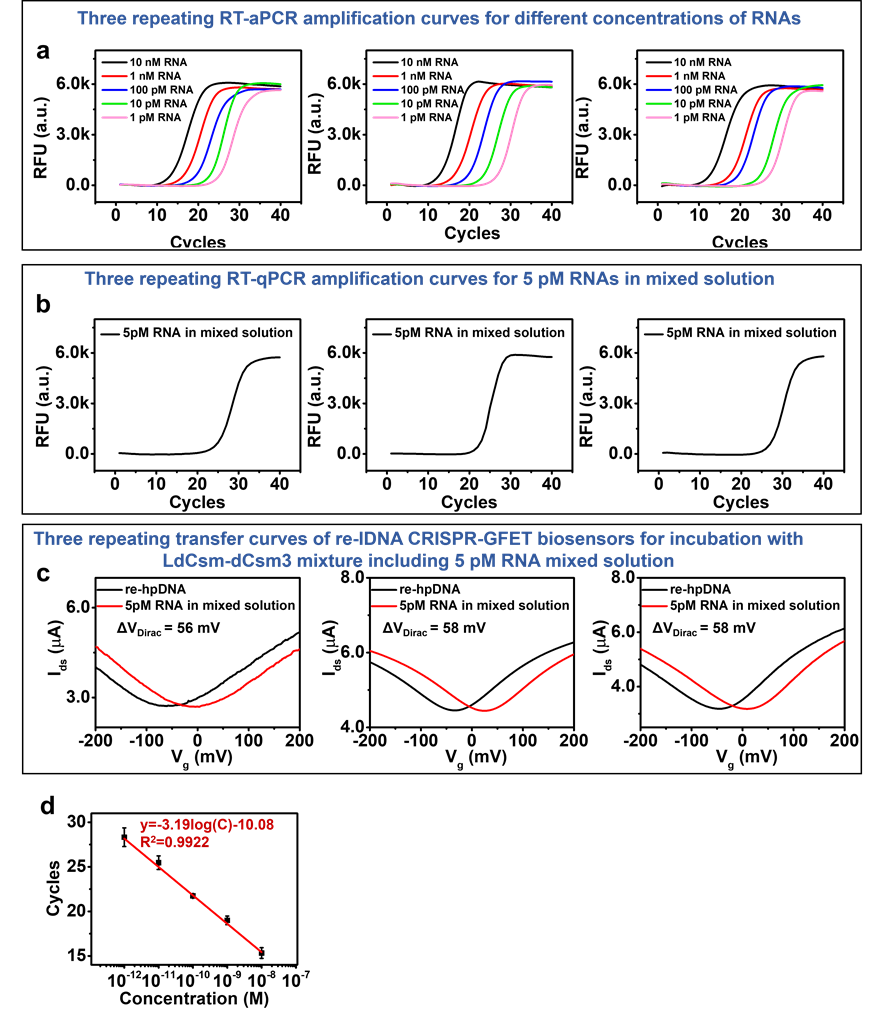


# Fig. S18 Comparison of the performance of the CRISPR-GFET biosensor and RT-qPCR assays. (a) Three repeating RT-qPCR amplification curves for different concentrations of RNAs. (b) Three repeating RT-qPCR amplification curves for 5 pM RNAs in mixed solution. (c) Three repeating transfer curves of CRISPR-GFET biosensors before and after incubation with LdCsm-dCsm3 mixture including 5 pM RNA mixed solution. (d) Calibration curves between the Ct and different levels of RNA, assessed according to Fig. S18a


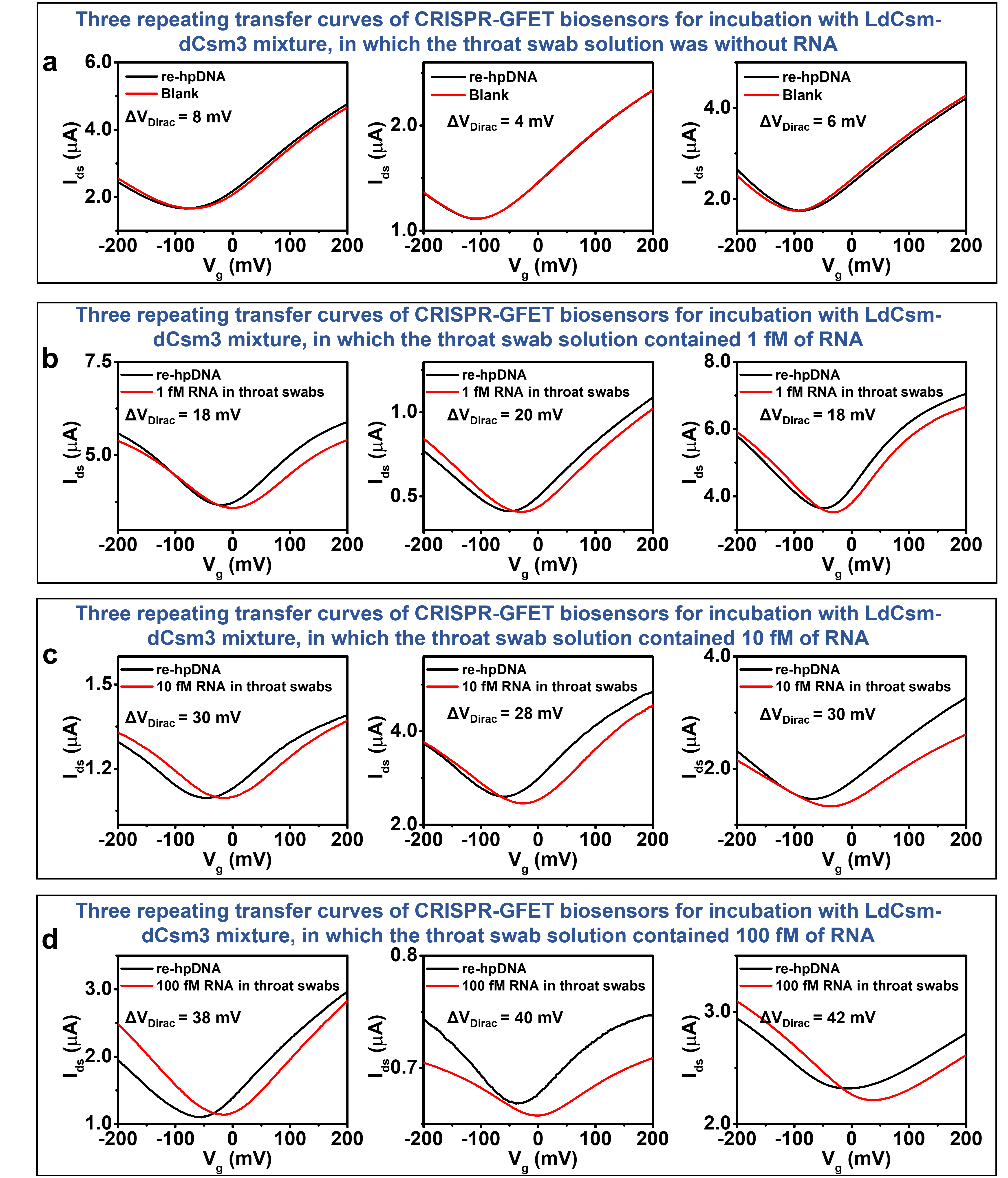


# Fig. S19 The transfer curves of CRISPR-GFET biosensors for incubation with LdCsm-dCsm3 mixture, in which RNA was added to the throat swab. (a)Three repeating transfer curves of CRISPR-GFET biosensors before and after incubation with LdCsm-dCsm3 mixture, in which the throat swab solution was without RNA. (b)Three repeating transfer curves of CRISPR-GFET biosensors before and after incubation with LdCsm-dCsm3 mixture, in which the throat swab solution contained 1 fM of RNA. (c) Three repeating transfer curves of CRISPR-GFET biosensors before and after incubation with LdCsm-dCsm3 mixture, in which the throat swab solution contained 10 fM of RNA. (d) Three repeating transfer curves of CRISPR-GFET biosensors before and after incubation with LdCsm-dCsm3 mixture, in which the throat swab solution contained 100 fM of RNA

#
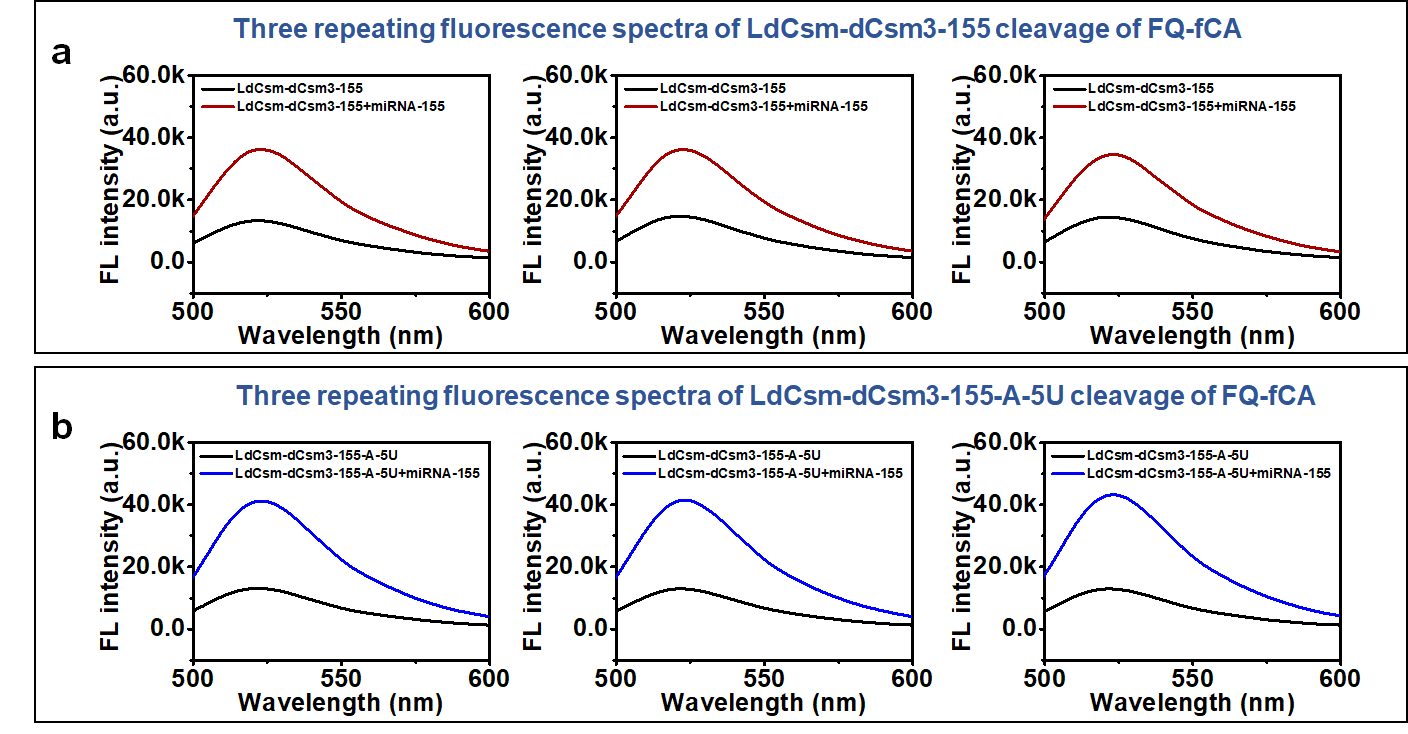


Fig. S20 Comparison of LdCsm-dCsm3-155/LdCsm-dCsm3-155-A-5U mediated cleavage. (**a**) Three repeating fluorescence spectra of LdCsm-dCsm3-155 cleavage of FQ-fCA in the absence/presence of target miRNA. (**b**) Three repeating fluorescence spectra of LdCsm-dCsm3-155-A-5U cleavage of FQ-fCA in the absence/presence of target miRNA


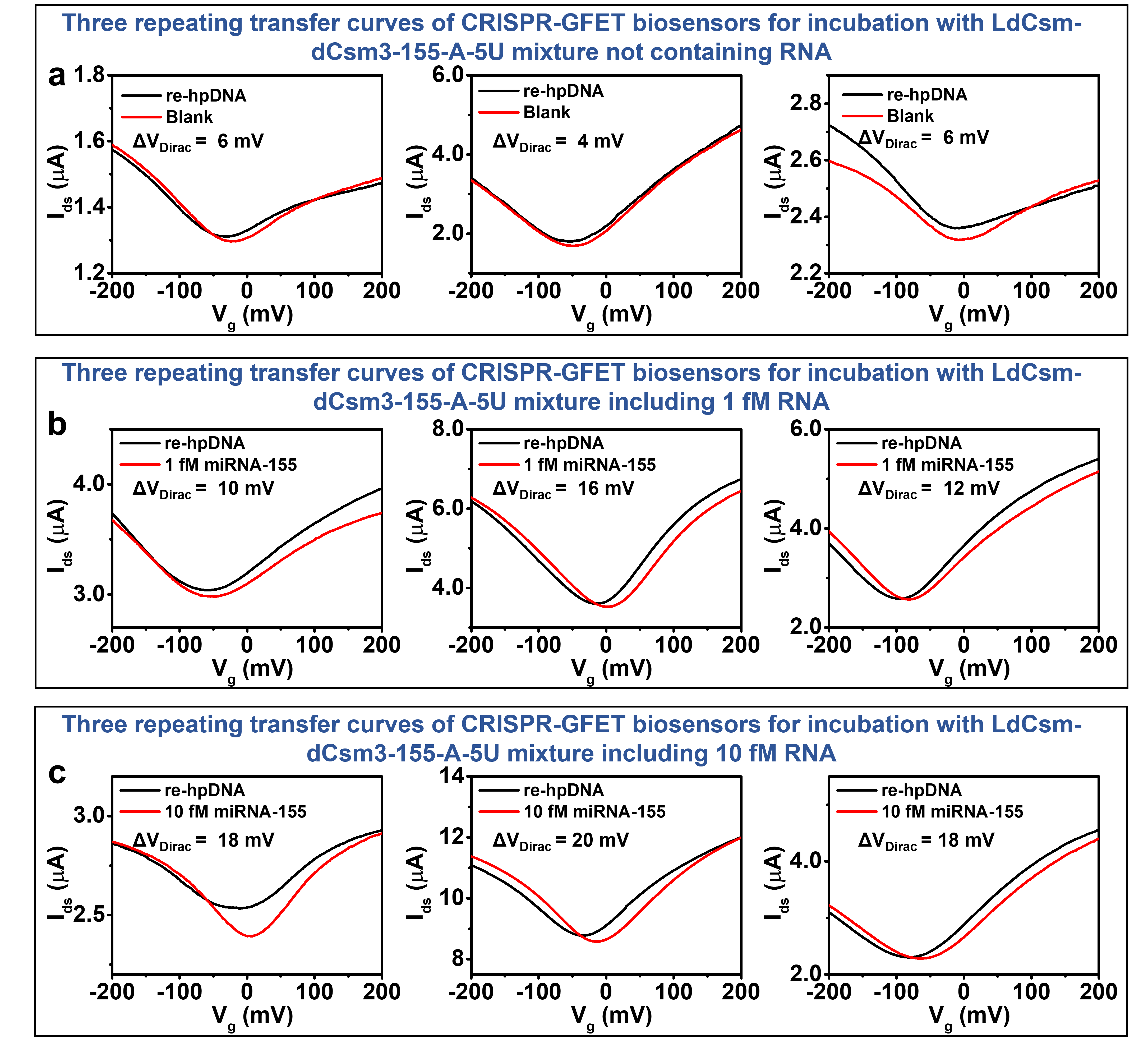


# Fig. S21 The transfer curves of CRISPR-GFET biosensors for incubation with LdCsm-dCsm3-155-A-5U mixture including different concentrations of miRNA. (a) Three repeating transfer curves of CRISPR-GFET biosensors before and after incubation with LdCsm-dCsm3-155-A-5U mixture not containing RNA. (b) Three repeating transfer curves of CRISPR-GFET biosensors before and after incubation with LdCsm-dCsm3-155-A-5U mixture including 1 fM RNA. (c) Three repeating transfer curves of CRISPR-GFET biosensors before and after incubation with LdCsm-dCsm3-155-A-5U mixture including 10 fM RNA.

#
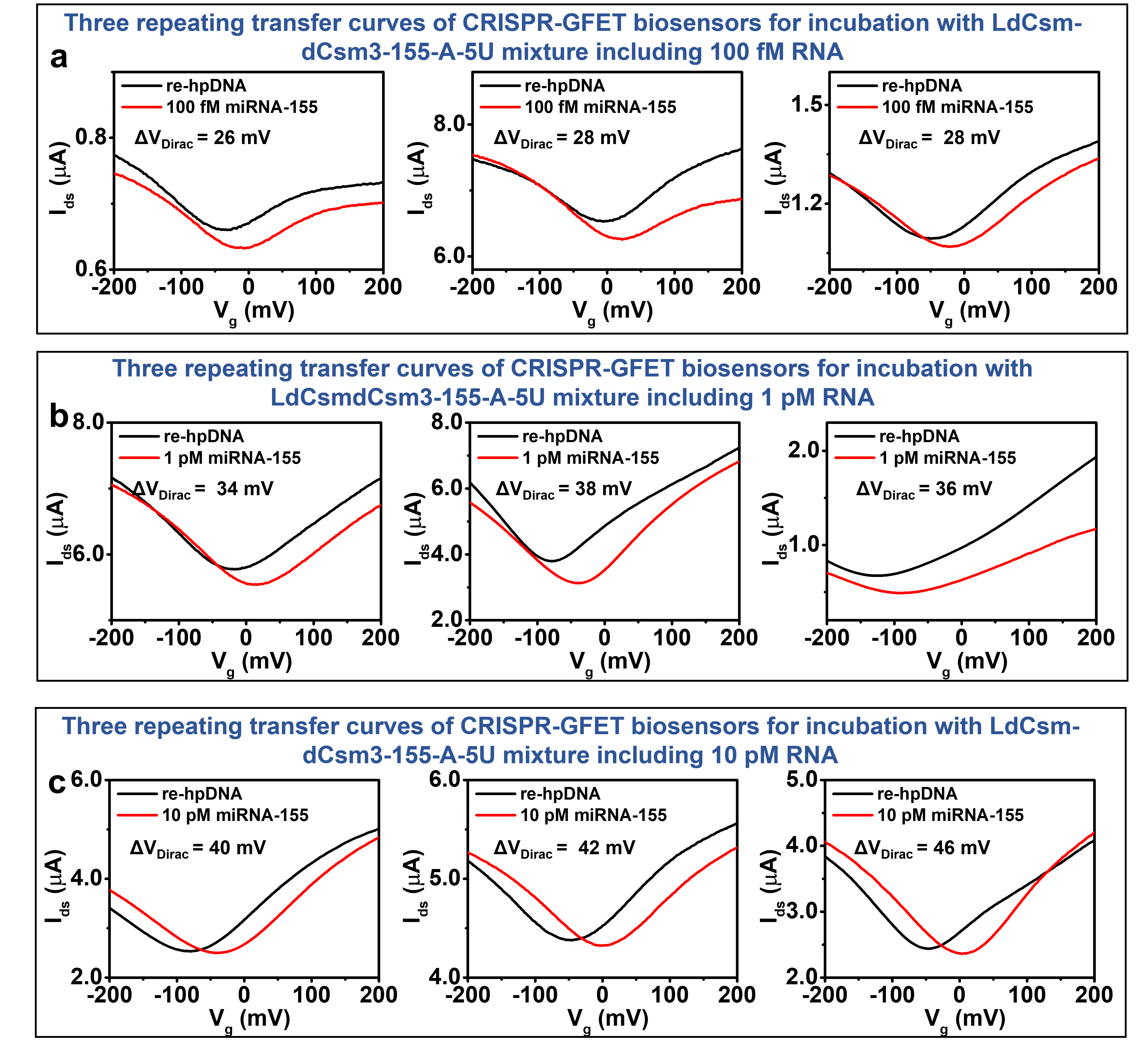


# Fig. S22 The transfer curves of CRISPR-GFET biosensors for incubation with LdCsm-dCsm3-155-A-5U mixture including different concentrations of miRNA. (a) Three repeating transfer curves of CRISPR-GFET biosensors before and after incubation with LdCsm-dCsm3-155-A-5U mixture including 100 fM RNA. (b) Three repeating transfer curves of CRISPR-GFET biosensors before and after incubation with LdCsm-dCsm3-155-A-5U mixture including 1 pM RNA. (c) Three repeating transfer curves of CRISPR-GFET biosensors before and after incubation with LdCsm-dCsm3-155-A-5U mixture including 10 pM RNA

#
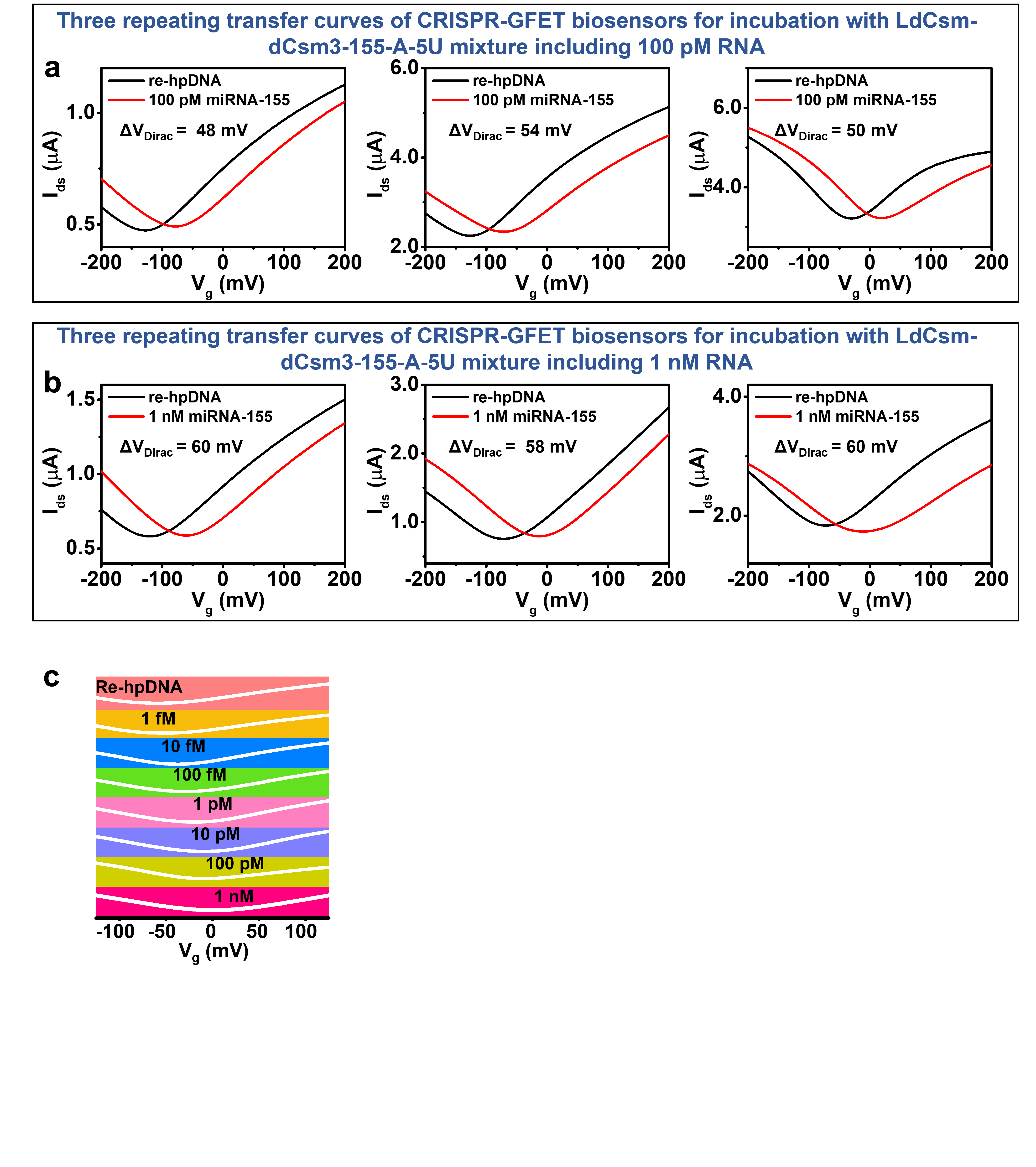


# Fig. S23 The transfer curves of CRISPR-GFET biosensors for incubation with LdCsm-dCsm3-155-A-5U mixture including different concentrations of miRNA. (a) Three repeating transfer curves of CRISPR-GFET biosensors before and after incubation with LdCsm-dCsm3-155-A-5U mixture including 100 pM RNA. (b) Three repeating transfer curves of CRISPR-GFET biosensors before and after incubation with LdCsm-dCsm3-155-A-5U mixture including 1 nM RNA. (c) Normalized transfer curves of the CRISPR-GFET biosensor after incubation with LdCsm-dCsm3-155-A-5U mixtures containing different concentrations of miRNA-155

#
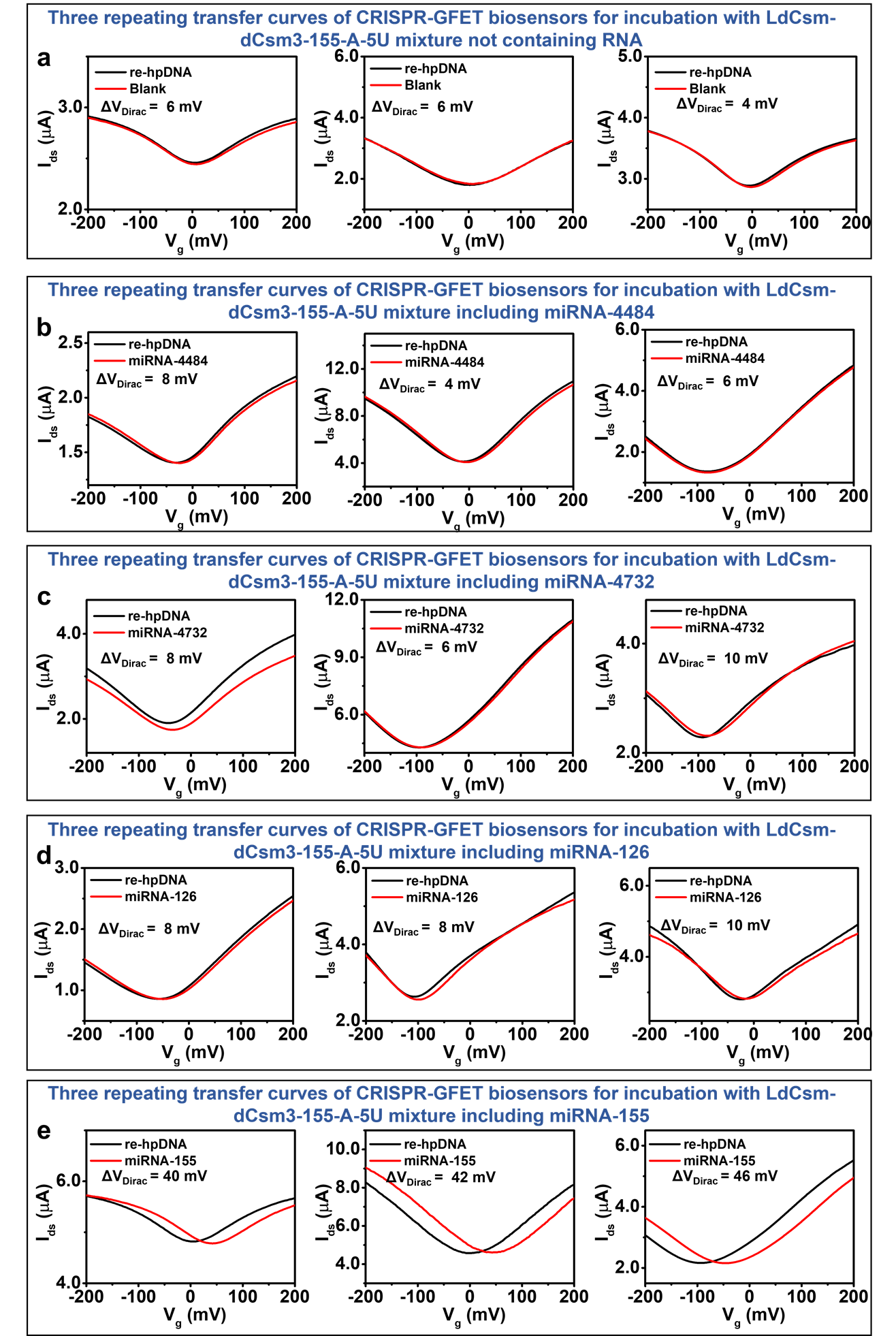


# Fig. S24 The transfer curves of CRISPR-GFET biosensors for incubation with LdCsm-dCsm3-155-A-5U mixture with miRNA-free (blank), non-target, or target miRNA. Three repeating transfer curves of CRISPR-GFET biosensors before and after incubation with LdCsm-dCsm3-155-A-5U mixture (a) not containing RNA, (b) including miRNA-4484, (c) including miRNA-4732, (d) including miRNA-126, (e) including target miRNA-155


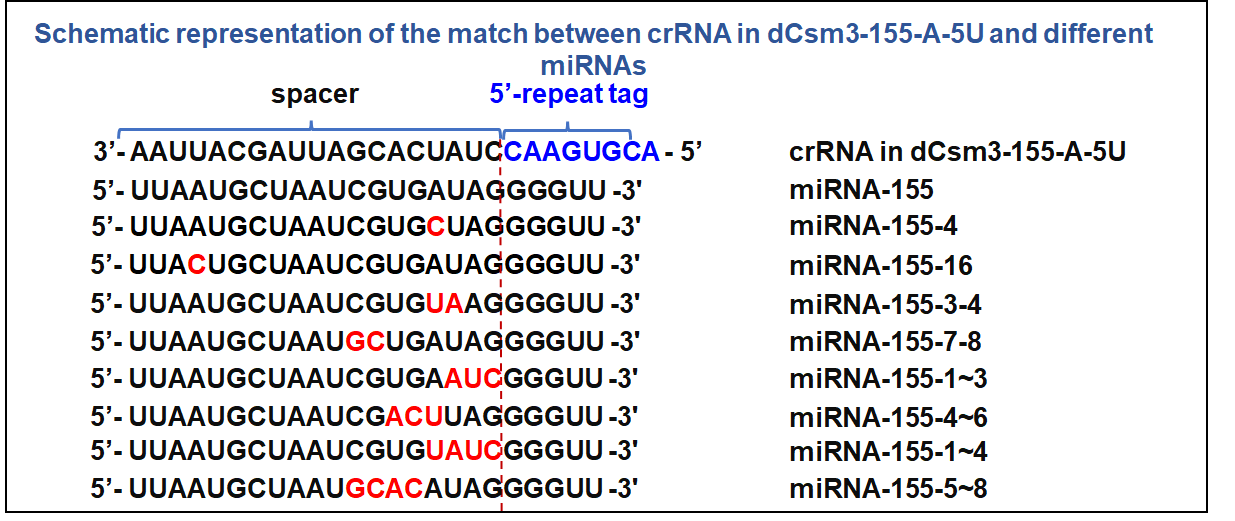


Fig. S25 Schematic representation of the match between crRNA in dCsm3-155-A-5U and different miRNAs. miRNA mismatched bases are shown in red


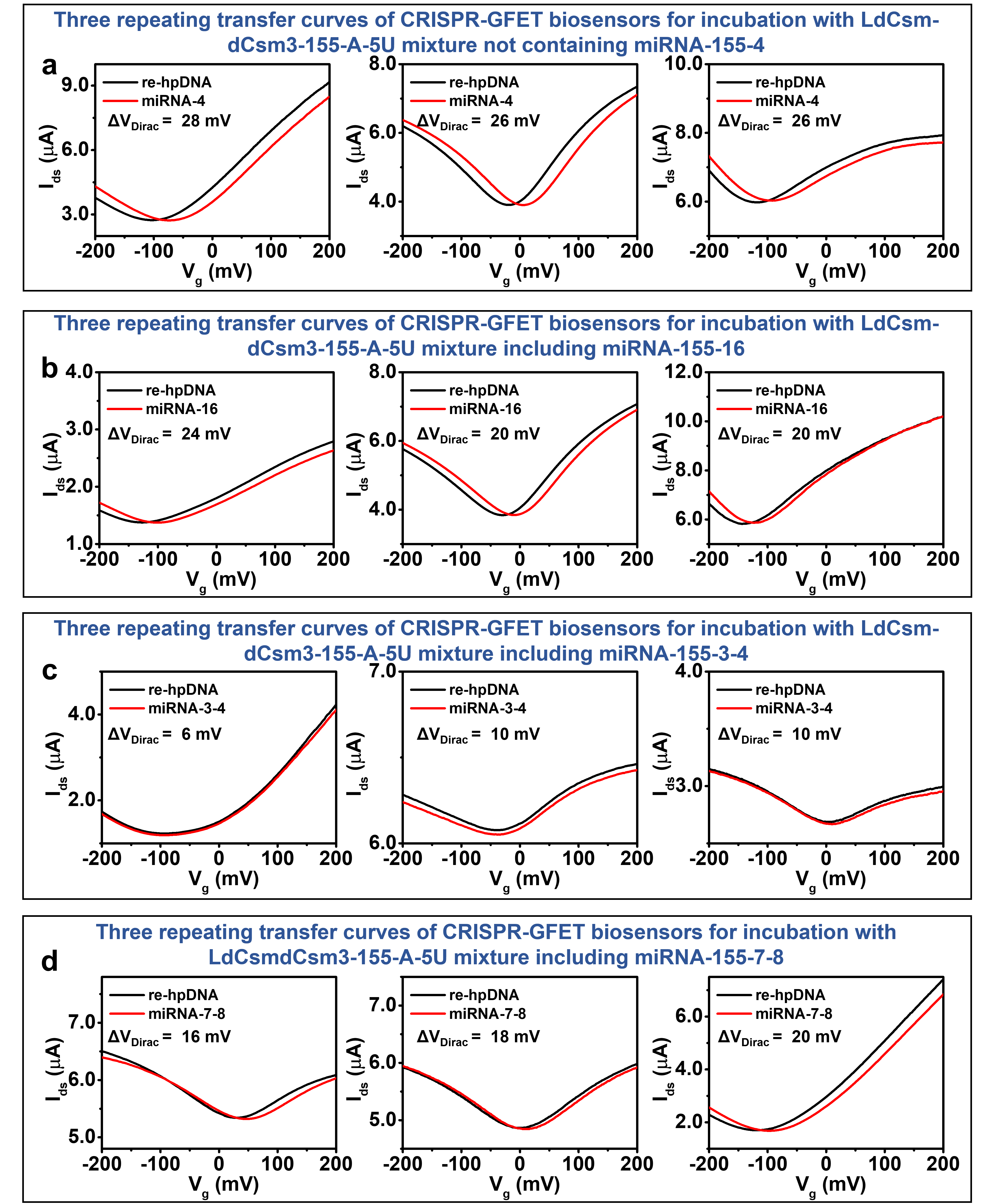


Fig. S26 CRISPR-GFET biosensor response to base mismatched miRNA. Three repeating transfer curves of CRISPR-GFET biosensors before and after incubation with LdCsm-dCsm3-155-A-5U mixture with (**a**) miRNA-155-4, (**b**) miRNA-155-16, (**c**) miRNA-155-3-4, (**d**) miRNA-155-7-8


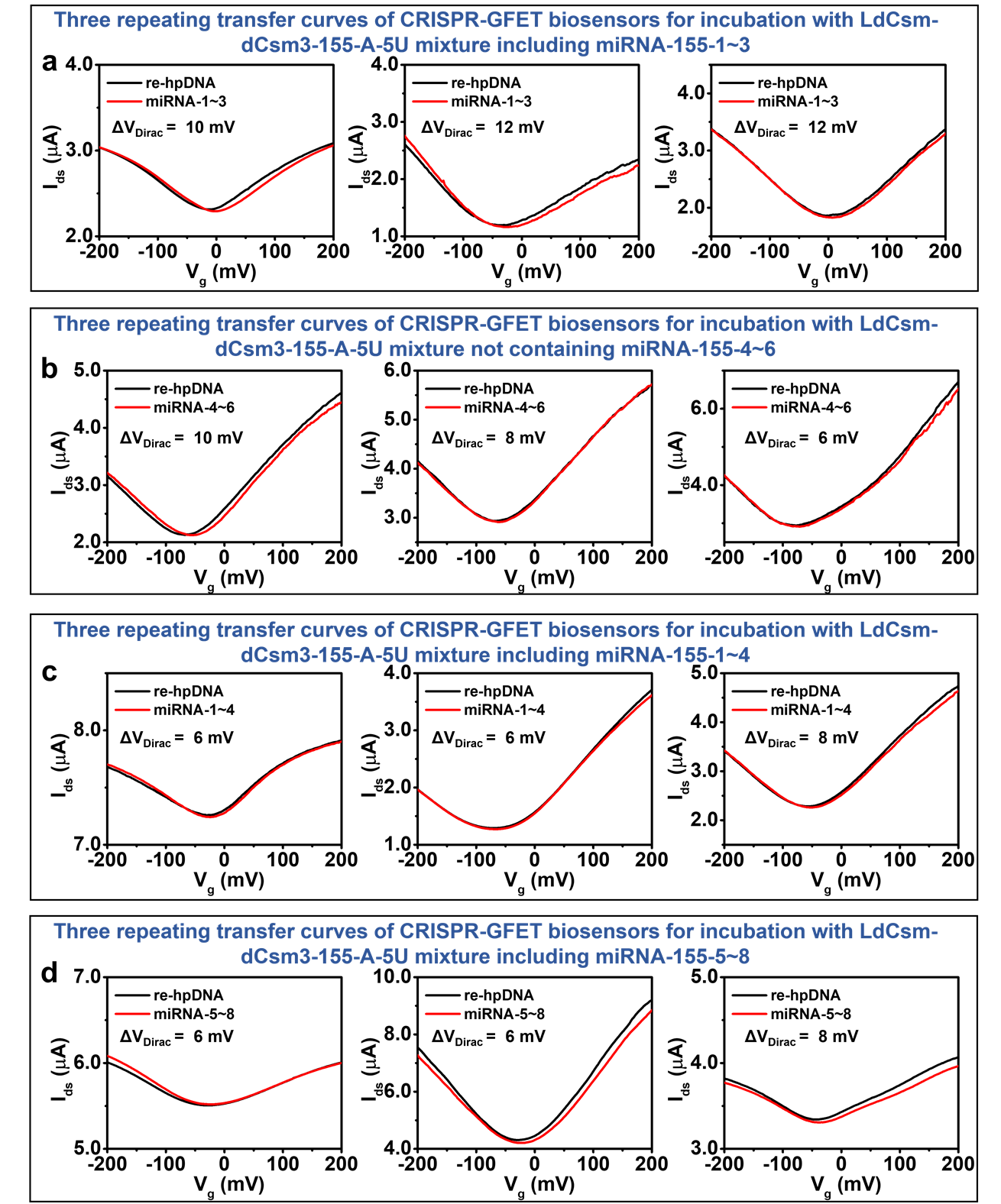


Fig. S27 CRISPR-GFET biosensor response to base mismatched miRNA. Three repeating transfer curves of CRISPR-GFET biosensors before and after incubation with LdCsm-dCsm3-155-A-5U mixture with (**a**) miRNA-155-1~3, (**b**) miRNA-155-4~6, (**c**) miRNA-155-1~4, (**d**) miRNA-155-5~8


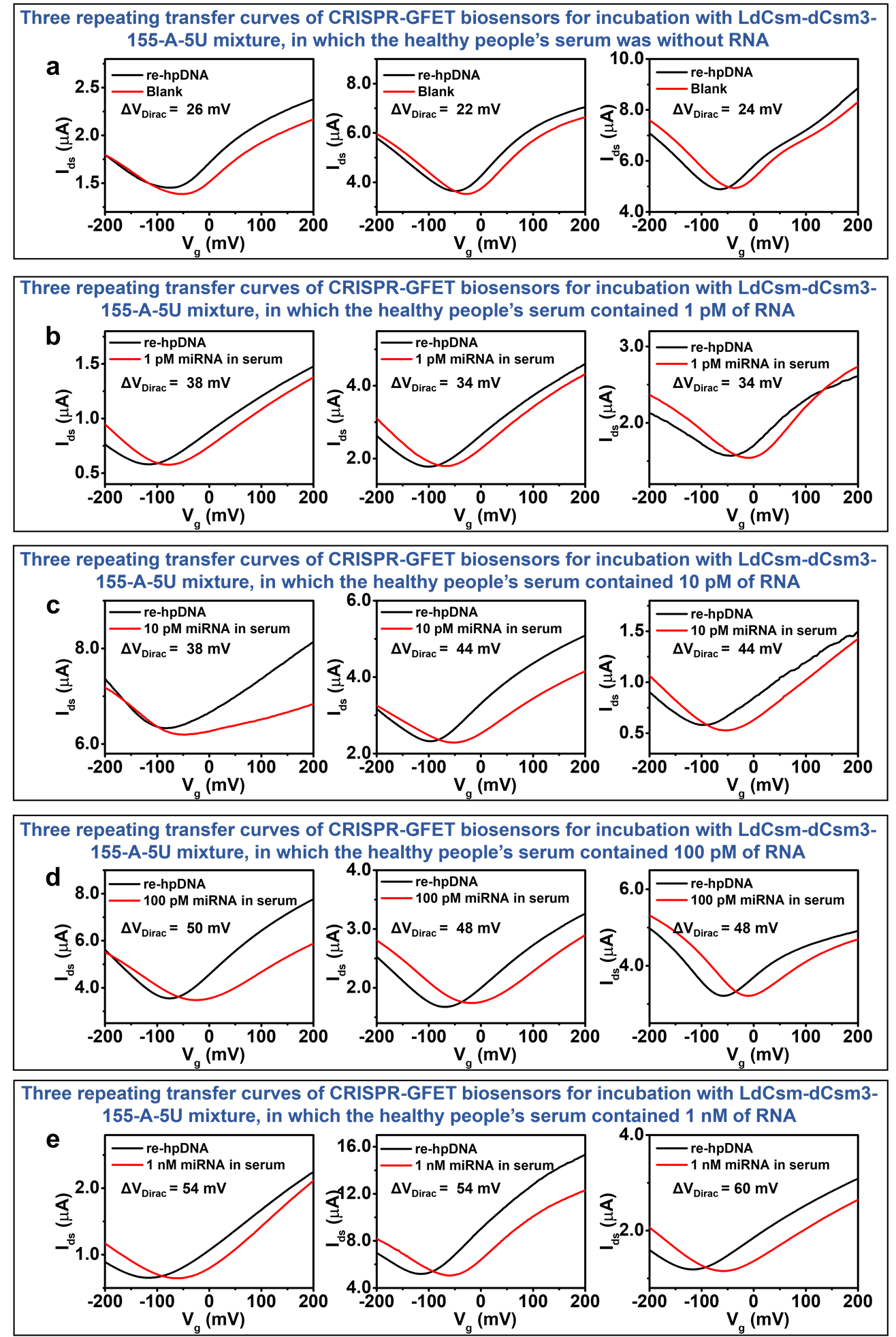


# Fig. S28 The transfer curves of CRISPR-GFET biosensors before and after incubation with LdCsm-dCsm3-155-A-5U mixture, in which miRNA was added to the healthy people’s serum. The transfer curves of CRISPR-GFET biosensors before and after incubation with LdCsm-dCsm3-155-A-5U mixture, in which the healthy people’s serum (a) was without RNA, (b) contained 1 pM of RNA, (c) contained 10 pM of RNA, (d) contained 100 pM of RNA, (e) contained 1 nM of RNA


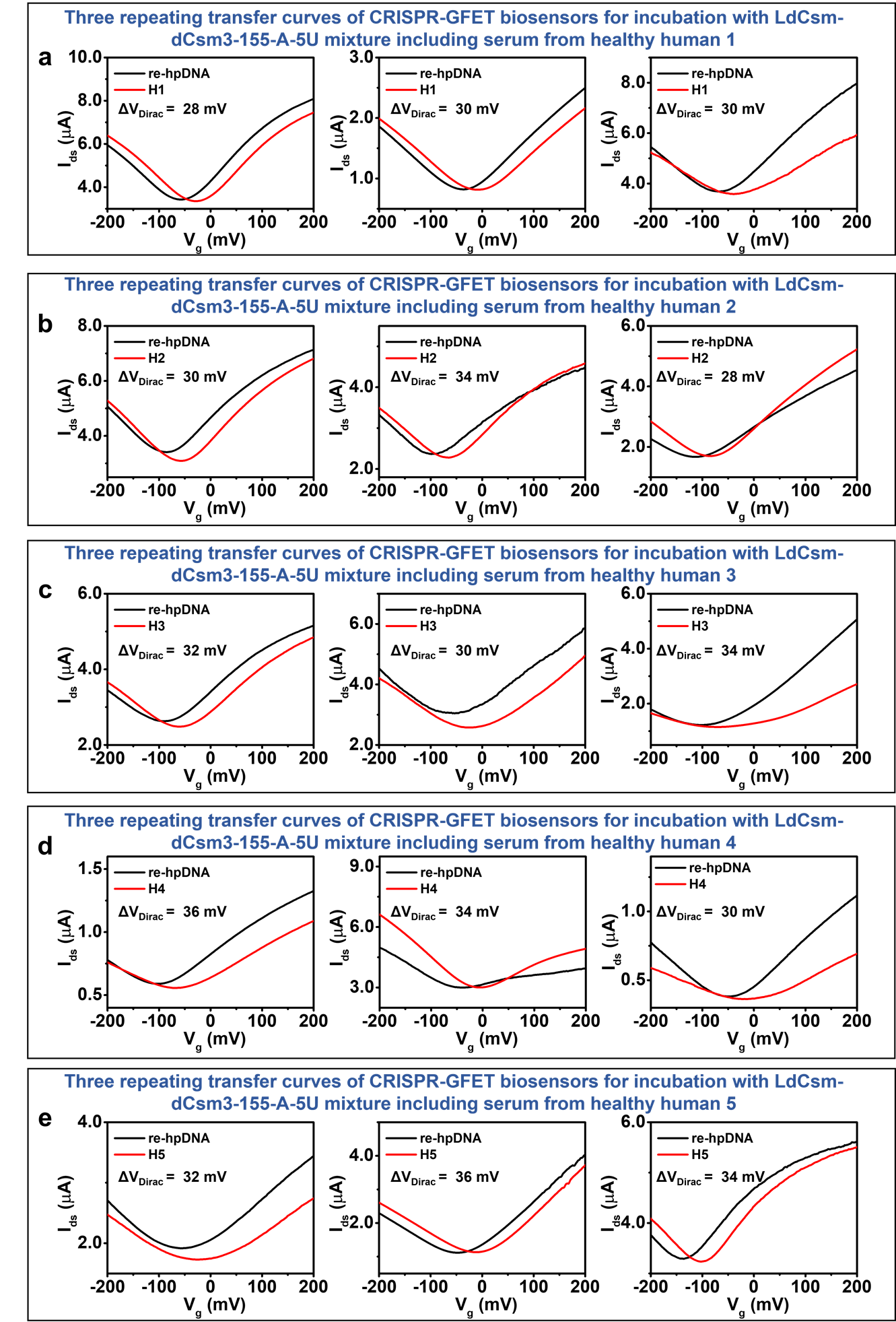


# Fig. S29 The transfer curves of CRISPR-GFET biosensors for incubation with LdCsm-dCsm3-155-A-5U mixture, where healthy human serum was used instead of miRNA solution. Three repeating transfer curves of CRISPR-GFET biosensors before and after incubation with LdCsm-dCsm3-155-A-5U mixture including serum from (a) healthy human 1, (b) healthy human 2, (c) healthy human 3, (d) healthy human 4, (e) healthy human 5


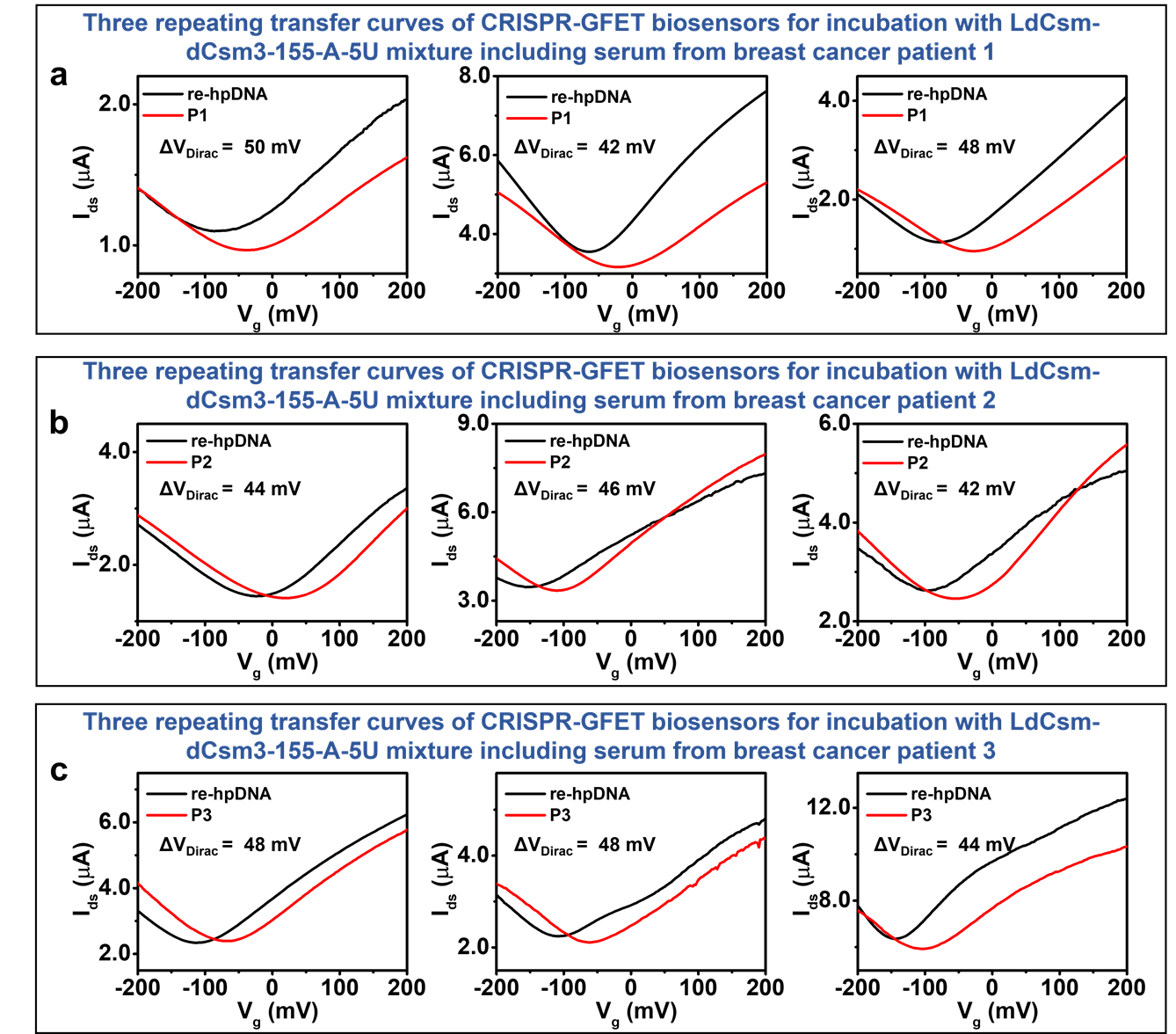


# Fig. S30 The transfer curves of CRISPR-GFET biosensors before and after incubation with LdCsm-dCsm3-155-A-5U mixture, where breast cancer patient serum was used instead of miRNA solution. Three repeating transfer curves of CRISPR-GFET biosensors before and after incubation with LdCsm-dCsm3-155-A-5U mixture including serum from (a) breast cancer patient 1, (b) breast cancer patient 2, (c) breast cancer patient 3

#
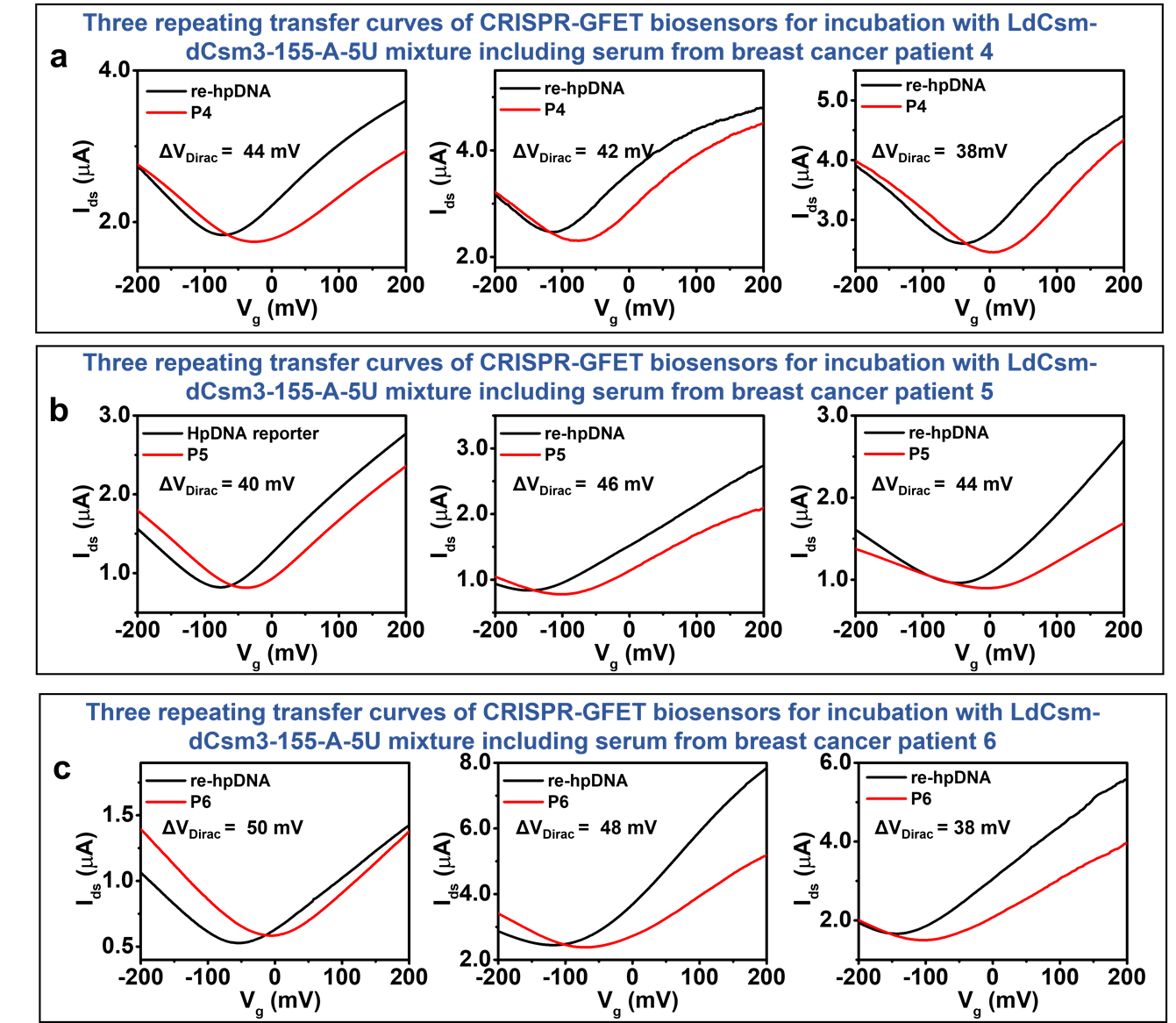


# Fig. S31 The transfer curves of CRISPR-GFET biosensors before and after incubation with LdCsm-dCsm3-155-A-5U mixture, where breast cancer patient serum was used instead of miRNA solution. Three repeating transfer curves of CRISPR-GFET biosensors before and after incubation with LdCsm-dCsm3-155-A-5U mixture including serum from (a) breast cancer patient 4, (b) breast cancer patient 5, (c) breast cancer patient 6

The levels of miRNA-155 in breast cancer serum samples were detected using the CRISPR-GFET biosensor to explore the potential for practical applications. Five healthy serum samples and six serum samples from breast cancer patients were used as the control and experimental groups, respectively. **Fig. S32a** summarizes the response of the biosensor to the electrical signals of healthy individuals versus breast cancer patients. Because miRNA-155 is an upregulated gene, Δ*V*_dirac_ is smaller in normal human serum samples than in serum samples from breast cancer patients. In the t-test, there is a significant difference between healthy individuals and breast cancer patients, with P<0.0001 (**Fig. S32b**). Thus, the results point to the potential of the developed CRISPR-GFET biosensor as a cancer diagnostic tool, as it can differentiate miRNA-155 levels in cancer patients and healthy individuals.


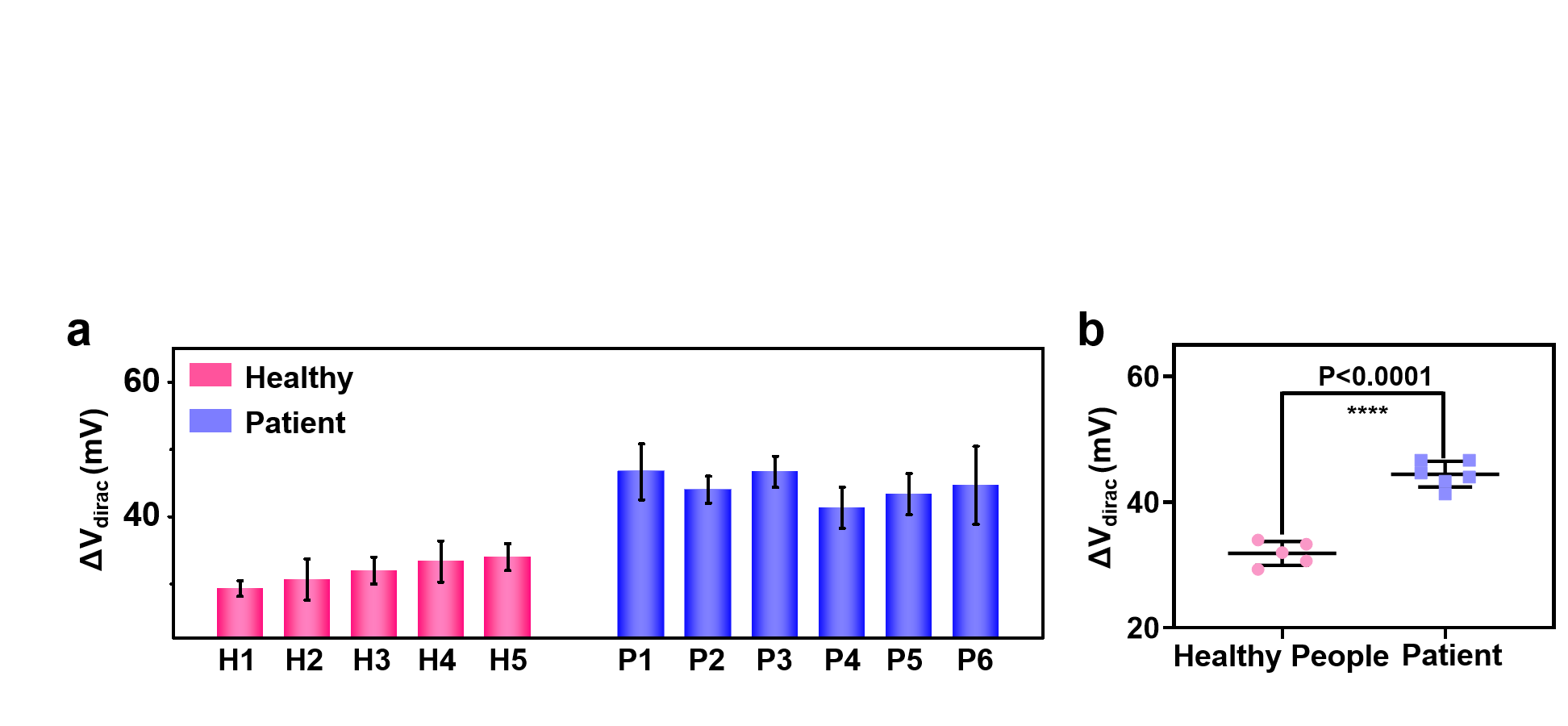


Fig. S32 Sensing signal of CRISPR-GFET biosensor for healthy and breast cancer patients. (**a**) The expression level of miRNA-155 in the serum was obtained from healthy and cancer patient samples. (**b**) Statistical comparison of real samples testing results in healthy individuals and breast cancer patients determined by an unpaired t-test. These were assessed by the transfer curves in **Figs. S29** and **S30-S31**

# Table S1 Comparison of CRISPR-GFET with state-of-the-art CRISPR biosensors

| Name | Amplification | Limit of detection | Detection time | CRISPR system | Receptor | Readout | Targets | Preparation cost |
| --- | --- | --- | --- | --- | --- | --- | --- | --- |
| DETECTR[S2] | RT-LAMP | 10 copies/μL | 50-60 min | CRISPR-Cas 12a | FB-DNA reporter | Fluorescence or lateral flow | RNA samples (SARS-CoV-2) | High |
| MiSHERLOCK[S3] | RT-RPA | 1000 copies/mL | 55 min (one pot) | CRISPR-Cas 12a | FQ-DNA reporter | Fluorescence | RNA samples (SARS-CoV-2) | High |
| SHINE[S4] | RT-RPA | 10 copies/μL | 60 min | CRISPR-Cas 13a | FQ-RNA reporter | Colorimetric /fluorescence | RNA samples (SARS-CoV-2) | High |
| Type III CRISPR-Cas systems[S5] | RT-LAMP | 200 copies/μL | <30 min | CRISPR-Cas 10 | FQ-RNA reporter | Fluorescence /colorimetric | RNA samples (SARS-CoV-2) | High |
| CRISPR-mediated platform[S6] | No need | 10 fM | 60 min | CRISPR-Cas12a/Cas13a | FQ-RNA/DNA reporter | Colorimetric, fluorometric, or luminescent | RNA samples (SARS-CoV-2) | Medium |
| CRISPR-mobile phone microscope[S7] | No need | 100 copies/μL | 30 min | CRISPR-Cas 13a | FQ-RNA reporter | Fluorescence | RNA samples (SARS-CoV-2) | Low |
| An RNA detection platform based on the LdCsm-dCsm3 system[S8] | No need | 2 nM | 30–60 min | CRISPR-Cas 10 | FQ-DNA reporter | Fluorescence | RNA sample (miRNA-155) | Low |
| Type III CRISPR-based method for RNA detection[S9] | No need | 15 fM | <20 min | CRISPR- Cas10 | FQ-RNA reporter | Fluorescence | RNA samples (SARS-CoV-2) | Low |
| SERS-CRISPR[S6] | No need | 1 fM | 30-40 min | CRISPR-Cas12a | - | SERE | RNA samples (SARS-CoV-2) | Medium |
|  |  |  |  |  |  |  |  |  |
| An ultrasensitive electrochemical biosensing platform[S10] | No need | 2.6 fM | 60 min | CRISPR-Cas13a | Hairpin DNA containing a secondary target DNA fragment | Electrochemical | RNA samples (miRNA-21) | Medium |
| CRISPR/Cas13a powered electrochemical biosensor[S11] | No need | 10 pM | < 4h | CRISPR-Cas 13a | FB-RNA reporter | Electrochemical | RNA samples (microRNA miR-19b and miR-20a) | Medium |
| E-CRISPR[S12] | No need | 50 pM | 30/60 min | CRISPR-Cas 12a | Mb-linear DNA reporter | Electrochemical | DNA samples (HPV-16 and parvovirus B19) and protein | Medium |
| CRISPR/Cas12a powered electrochemical DNA sensor[S13] | No need | 60 pM | 30 min | CRISPR-Cas 12a | Mb-hairpin DNA reporter | Electrochemical | DNA sample (HPV-18 and HPV-16) | Medium |
| CRISPR-E[S14] | No need | 10 pM | 180 min | CRISPR-Cas 13a | Single strand DNA-RNA reporter | Electrochemical | RNA samples miRNA-19b | Medium |
| Cas12a-based ECL biosensor[S15] | No need | 0.48 pM | 70 min | CRISPR-Cas 12a | SH-ssDNA-Fc | Electrochemiluminescence | DNA sample (HPV-16 DNA) | Medium |
| signal-switchable ECL biosensor[S16] | No need | 30 fM/0.32 pM | 60 min | CRISPR-Cas 12a | - | Electrochemiluminescence | DNA sample HIV/HPV DNAs | Medium |
| CRISPR-chip[S17] | No need | 1.7 fM | 15 min | CRISPR-Cas 9 | / | Electrical | gDNA from cell lines and Duchenne muscular dystrophy patients | Medium |
| Cas12a-GFET[S18] | No need | 2.42 aM | 30 min | CRISPR-Cas12a | / | Electrical | *Mycobacterium tuberculosis* | Medium |
| This work | No need | 200 aM | 45 min | CRISPR-Cas 10 | Hairpin DNA reporter | Electrical | RNA samples (RNA and miRNA-155) | Low (~$1.16/piece) |

**Table S2** Comparison of detection RNA performance of CRISPR-GFET biosensor and RT-qPCR assay

| Method | RT-qPCR | CRISPR-GFET |
| --- | --- | --- |
| Standard concentration | 5 pM | 5 pM |
| Response signal | Ct=26.18 | *ΔV*_dirac_=57.33 mV |
| Detection concentration | 4.329 pM | 4.870 pM |
| Recovery | 86.58% | 97.40% |
| RSD | 7.60% | 4.03% |

Table S3 **Determination of RNA in throat swab.**

| Sample | Standard concentration | Detection concentration | Recovery | RSD |
| --- | --- | --- | --- | --- |
| Sample 1 | 1 fM | 0.8168 fM | 81.68% | 6.19% |
| Sample 2 | 10 fM | 8.977 fM | 89.77% | 3.94% |
| Sample 3 | 100 fM | 98.81 fM | 98.81% | 5.00% |

# Table S4 DNA, RNA, and miRNA sequences used in this study

| Annotation | Name | Sequence (5'-3') |
| --- | --- | --- |
| Fluorescent reporters | FQ-fT | FAM-TTTTTTTTTTTTTTTT-BHQ1 |
|  | FQ-fCT | FAM-CTCTCTCTCTCTCTCT-BHQ1 |
|  | FQ-fCA | FAM-CACACACACACACACA-BHQ1 |
|  | re-FAM-lDNA | SH C6-CACACACACACACACA-FAM |
| DNA reporters | re-lDNA | SH C6-CACACACACACACACA |
|  | re-hpDNA | SH C6-TCAGCGTTCCTTTACCATTTTTTTAACTTATTTGGTTTTTTTTTT |
| DNA primer | Stem-loop primer | GTCGTATCCAGTGCAGGGTCCGAGGTATTCGCACTGGATACGACATTCTT |
|  | Forward primer | AACCTCCAGCGCAGTAAGGATG |
|  | Reverse primer | ATCCAGTGCAGGGTCCGAGG |
| RNAs | 64nt RNA | UAUUGCAGCAGUACGCACACAAUCGAAGCGCAGUAAGGAUGGCUAGUGUAACUAGCAAGAAUAC |
|  | 46nt RNA | ACACAAUCGAAGCGCAGUAAGGAUGGCUAGUGUAACUAGCAAGAAU |
|  | 36nt RNA (RNA) | AGCGCAGUAAGGAUGGCUAGUGUAACUAGCAAGAAU |
|  | 28nt RNA | AAGGAUGGCUAGUGUAACUAGCAAGAAU |
|  | 23nt RNA | UGGCUAGUGUAACUAGCAAGAAU |
|  | 20nt RNA | CUAGUGUAACUAGCAAGAAU |
|  | RNA-1 | UAUUGCAGCAGUACGCACACAAUCGAAGCGCAGUAAGGAUGGCUAGUGUAACUAGC |
|  | RNA-2 | UAUUGCAGCAGUACGCACACAAUCGAAGCGCAGUAAGGAUGGCUAGUGUAACUAGC |
|  | RNA-3 | AAAUGUUAAAAACACUAUUAGCAUAAGCAGUUGUGGCAUCUCCUGAUGAG |
|  | Unspecific RNA | FAM-UGUUAAGUCUGGUUUCCCUCCAGGGUAUCUAAGCUUUGAA |
|  | RNA | FAM-AGCGCAGUAAGGAUGGCUAGUGUAACUAGCAAGAAU |
| miRNAs | miRNA-155 | UUAAUGCUAAUCGUGAUAGGGGUU |
|  | miRNA-126 | UCGUACCGUGAGUAAUAAUGCG |
|  | miRNA-4732 | UGUAGAGCAGGGAGCAGGAAGCU |
|  | miRNA-4484 | AAAAUGAAAUGAGCCCAGCCCA |
|  | miRNA-155-4 | UUAAUGCUAAUCGUGCUAGGGGUU |
|  | miRNA-155-16 | UUACUGCUAAUCGUGAUAGGGGUU |
|  | miRNA-155-3-4 | UUAAUGCUAAUCGUGUAAGGGGUU |
|  | miRNA-155-7-8 | UUAAUGCUAAUGCUGAUAGGGGUU |
|  | miRNA-155-1~3 | UUAAUGCUAAUCGUGAAUCGGGUU |
|  | miRNA-155-4~6 | UUAAUGCUAAUCGACUUAGGGGUU |
|  | miRNA-155-1~4 | UUAAUGCUAAUCGUGUAUCGGGUU |
|  | miRNA-155-5~8 | UUAAUGCUAAUGCACAUAGGGGUU |

**Equation S1:**

$\lambda_{D}=\frac{1}{\sqrt{4\pi l_{B}\sum_{i} p_{i}z_{i}^{2}}}$ (S1)

In Equation S1, *l*_B_ is the Bjerrum length, Σ*_i_* is the sum over all ion species and *ρ_i_* and *z_i_* are the concentration and valence of ion species *i*, respectively [S19].

**Supplementary References**

1. F. Clerici, M. Fontana, S. Bianco, M. Serrapede, F. Perrucci et al., *In situ* MoS_2_ decoration of laser-induced graphene as flexible supercapacitor electrodes. ACS Appl. Mater. Interfaces **8**(16), 10459–10465 (2016). <https://doi.org/10.1021/acsami.6b00808>
2. J.P. Broughton, X. Deng, G. Yu, C.L. Fasching, V. Servellita et al., CRISPR–Cas12-based detection of SARS-CoV-2. Nat. Biotechnol. **38**(7), 870–874 (2020). <https://doi.org/10.1038/s41587-020-0513-4>
3. H. de Puig, R.A. Lee, D. Najjar, X. Tan, L.R. Soenksen et al., Minimally instrumented sherlock (misherlock) for crispr-based point-of-care diagnosis of sars-cov-2 and emerging variants. Sci. Adv. **7**(32), eabh2944 (2021). <https://doi.org/doi:10.1126/sciadv.abh2944>
4. J. Arizti-Sanz, C.A. Freije, A.C. Stanton, B.A. Petros, C.K. Boehm et al., Streamlined inactivation, amplification, and Cas13-based detection of SARS-CoV-2. Nat. Commun. **11**(1), 5921 (2020). <https://doi.org/10.1038/s41467-020-19097-x>
5. A. Santiago-Frangos, L.N. Hall, A. Nemudraia, A. Nemudryi, P. Krishna et al., Intrinsic signal amplification by type III CRISPR-Cas systems provides a sequence-specific SARS-CoV-2 diagnostic. Cell Rep. Med. **2**(6), 100319 (2021). <https://doi.org/10.1016/j.xcrm.2021.100319>
6. D. Samanta, S. B. Ebrahimi, N. Ramani, C. A. Mirkin, Enhancing crispr-cas-mediated detection of nucleic acid and non-nucleic acid targets using enzyme-labeled reporters. J. Am. Chem. Soc. **144**(36), 16310-16315 (2022). <https://doi.org/10.1021/jacs.2c07625>
7. P. Fozouni, S. Son, M. Díaz de León Derby, G.J. Knott, C.N. Gray et al., Amplification-free detection of SARS-CoV-2 with CRISPR-Cas13a and mobile phone microscopy. Cell **184**(2), 323–333.e9 (2021). <https://doi.org/10.1016/j.cell.2020.12.001>
8. Z. Yu, J. Xu, Q. She, Harnessing the ldcsm rna detection platform for efficient microrna detection. Int. J. Mol. Sci. **24**(3), 2857 (2023). <https://doi.org/10.3390/ijms24032857>
9. A. Nemudraia, A. Nemudryi, M. Buyukyoruk, A.M. Scherffius, T. Zahl et al., Sequence-specific capture and concentration of viral RNA by type III CRISPR system enhances diagnostic. Nat. Commun. **13**(1), 7762 (2022). <https://doi.org/10.1038/s41467-022-35445-5>
10. Y. Cui, S. Fan, Z. Yuan, M. Song, J. Hu et al., Ultrasensitive electrochemical assay for microRNA-21 based on CRISPR/Cas13a-assisted catalytic hairpin assembly. Talanta **224**, 121878 (2021). <https://doi.org/10.1016/j.talanta.2020.121878>
11. R. Bruch, J. Baaske, C. Chatelle, M. Meirich, S. Madlener et al., Crispr/cas13a-powered electrochemical microfluidic biosensor for nucleic acid amplification-free mirna diagnostics. Adv. Mater. **31**(51), 1905311 (2019). <https://doi.org/10.1002/adma.201905311>
12. Y. Dai, R.A. Somoza, L. Wang, J.F. Welter, Y. Li et al., Exploring the trans-cleavage activity of CRISPR-Cas12a (cpf1) for the development of a universal electrochemical biosensor. Angew. Chem. Int. Ed. **58**(48), 17399–17405 (2019). <https://doi.org/10.1002/anie.201910772>
13. D. Zhang, Y. Yan, H. Que, T. Yang, X. Cheng et al., CRISPR/Cas12a-mediated interfacial cleaving of hairpin DNA reporter for electrochemical nucleic acid sensing. ACS Sens. **5**(2), 557–562 (2020). <https://doi.org/10.1021/acssensors.9b02461>
14. Y. Xu, C. Wang, G. Liu, X. Zhao, Q. Qian et al., Tetrahedral DNA framework based CRISPR electrochemical biosensor for amplification-free miRNA detection. Biosens. Bioelectron. **217**, 114671 (2022). <https://doi.org/10.1016/j.bios.2022.114671>
15. P.-F. Liu, K.-R. Zhao, Z.-J. Liu, L. Wang, S.-Y. Ye et al., Cas12a-based electrochemiluminescence biosensor for target amplification-free DNA detection. Biosens. Bioelectron. **176**, 112954 (2021). <https://doi.org/10.1016/j.bios.2020.112954>
16. K.-R. Zhao, L. Wang, P.-F. Liu, X.-M. Hang, H.-Y. Wang et al., A signal-switchable electrochemiluminescence biosensor based on the integration of spherical nucleic acid and CRISPR/Cas12a for multiplex detection of HIV/HPV DNAs. Sens. Actuat. B Chem. **346**, 130485 (2021). <https://doi.org/10.1016/j.snb.2021.130485>
17. R. Hajian, S. Balderston, T. Tran, T. deBoer, J. Etienne et al., Detection of unamplified target genes via crispr–cas9 immobilized on a graphene field-effect transistor. Nat. Biomed. Eng. **3**(6), 427-437 (2019). <https://doi.org/10.1038/s41551-019-0371-x>
18. W. Wang, H. Du, C. Dai, H. Ma, S. Luo et al., Amplification-free detection of *Mycobacterium tuberculosis* using CRISPR-Cas12a and graphene field-effect transistors. Nanoscale **17**(8), 4603–4609 (2025). <https://doi.org/10.1039/d4nr03852e>
19. Y. Jiang, B. Tian, Inorganic semiconductor biointerfaces. Nat. Rev. Mater. **3**(12), 473–490 (2018). <https://doi.org/10.1038/s41578-018-0062-3>
